# A phylogenomic profile of hemerythrins, the nonheme diiron binding respiratory proteins

**DOI:** 10.1186/1471-2148-8-244

**Published:** 2008-09-02

**Authors:** Xavier Bailly, Stefano Vanin, Christine Chabasse, Kenji Mizuguchi, Serge N Vinogradov

**Affiliations:** 1 Station Biologique de Roscoff, 29680, Roscoff, France; 2 Department of Biology, University of Padova, 35131, Padova, Italy; 3 Division of Vascular Surgery, UCSF VA Medical Center, San Francisco, CA 94121, USA; 4 National Institute of Biomedical Innovation, Ibaraki, Osaka, 567-0085, Japan; 5 Department of Biochemistry and Molecular Biology, Wayne State University School of Medicine, Detroit, MI 48201, USA

## Abstract

**Background:**

Hemerythrins, are the non-heme, diiron binding respiratory proteins of brachiopods, priapulids and sipunculans; they are also found in annelids and bacteria, where their functions have not been fully elucidated.

**Results:**

A search for putative Hrs in the genomes of 43 archaea, 444 bacteria and 135 eukaryotes, revealed their presence in 3 archaea, 118 bacteria, several fungi, one apicomplexan, a heterolobosan, a cnidarian and several annelids. About a fourth of the Hr sequences were identified as N- or C-terminal domains of chimeric, chemotactic gene regulators. The function of the remaining single domain bacterial Hrs remains to be determined. In addition to oxygen transport, the possible functions in annelids have been proposed to include cadmium-binding, antibacterial action and immunoprotection. A Bayesian phylogenetic tree revealed a split into two clades, one encompassing archaea, bacteria and fungi, and the other comprising the remaining eukaryotes. The annelid and sipunculan Hrs share the same intron-exon structure, different from that of the cnidarian Hr.

**Conclusion:**

The phylogenomic profile of Hrs demonstrated a limited occurrence in bacteria and archaea and a marked absence in the vast majority of multicellular organisms. Among the metazoa, Hrs have survived in a cnidarian and in a few protostome groups; hence, it appears that in metazoans the Hr gene was lost in deuterostome ancestor(s) after the radiata/bilateria split. Signal peptide sequences in several Hirudinea Hrs suggest for the first time, the possibility of extracellular localization. Since the α-helical bundle is likely to have been among the earliest protein folds, Hrs represent an ancient family of iron-binding proteins, whose primary function in bacteria may have been that of an oxygen sensor, enabling aerophilic or aerophobic responses. Although Hrs evolved to function as O_2 _transporters in brachiopods, priapulids and sipunculans, their function in annelids remains to be elucidated. Overall Hrs exhibit a considerable lack of evolutionary success in metazoans.

## Background

Three types of respiratory proteins occur in present day metazoans: hemoglobin, ubiquitous among vertebrates and found in most prokaryotes and eukaryotes [1,2], hemocyanin, present mostly in arthropods and molluscs [3], and hemerythrin (Hr) [4]. The latter occurs in coelomocytes in circulating coelomic fluid and in muscle tissue as MHr, and was originally thought to be limited to three minor protostome phyla, the Sipuncula, Brachiopoda and Priapulida, and one annelid species [4-6]. Over the last twenty years, cytoplasmic Hrs have been reported in all three annelid groups, polychaetes [7-9], oligochaetes [10], and hirudinae [11-13]. A recent molecular phylogenetic study of sipunculan Hrs has shown them to have a close relationship to annelid Hrs [14]. A Hr sharing > 43% identity with annelid Hrs, was found in a search for antigen-related genes expressed in the heterolobosan *Naegleria fowleri*, the causative agent of primary amoebic meningoencephalitis [15,16]. In the last few years, Hrs have been found in bacteria, as a single domain protein in the γ-proteobacterium *Methylococcus capsulatus *[17], and as a C-terminal domain of a chimeric, methyl accepting chemotaxis protein in the sulfate-reducing δ-proteobacterium *Desulfovibrio vulgaris *[18].

The crystal structures of metazoan Hrs and MHrs are very similar [19,20], a four helix bundle of antiparallel α-helices (A through D) formed by polypeptide chains of 113aa and 118aa, respectively. The active site consists of two oxo-/hydroxo-bridged Fe atoms (Fig. s1 in Additional file 1). Fe1 is coordinated to three His side-chain groups in helices C and D, and Fe2 is coordinated to two His side-chain groups in helices A and B; the carboxylate side-chain groups of a Glu in helix C and an Asp in helix D, bridge both irons. Although the *D. vulgaris *Hr domain is somewhat longer than metazoan Hrs, 130aa, it has a very similar structure [21].

We report below the results of an exhaustive search for putative Hrs within the available genomes from the three kingdoms of life and the isolation of Hr genes in several annelids. Furthermore, we describe for the first time the intron-exon structure of metazoan Hr genes, provide evidence for an extracellular occurrence of leech Hr, and discuss the implications of the phylogenomic distribution of Hrs.

## Results

### Eukaryote Hrs

The previously known and the newly identified Hr sequences are listed in Additional file 1 in Table s1, together with their manual alignments shown in Fig. s2. In addition to the metazoan Hrs identified earlier [14], we have sequenced putative Hr genes from the sipunculan *S. nudus *(Hr: AM886444 and MHr AM886445), the deep-sea hydrothermal vent vestimentiferan *R. pachyptila *(AM886446) and the polychaete *S. armiger *(AM886447). Blastp searches revealed putative Hrs in the apicomplexan *Plasmodium yoelii*, the heterolobosan *Naegleria gruberi*, the cnidarian *Nematostella vectensis *(Radiata), the oligochaete *Lumbricus rubellus*, the polychaete *Periserrula leucophryna*, and the hirudineans *Haementeria depressa *and *Helobdella robusta*. Although most eukaryotes have one or two Hrs, the genomes of *N. gruberi *and *H. robusta *have 5 and 13 Hrs, respectively. No Hrs were found in the genome of the polychaete *Capitella sp.I *http://www.jgi-psf.org/Capca1/Capca1.info.html. Putative Hrs were also found in 10 Ascomycota and 3 Basidiomycota, out of a total of > 50 fungal genomes: all have very similar sequences, substantially different from other Hrs. We have used FUGUE, which recognizes sequence-structure homology using environment-specific substitution tables and structure-dependent gap penalties [22] to define whether they should be considered to be Hrs. Although their FUGUE Z scores range from 6 to 8, interpreted as a certain assignment [22], they all share the following alterations in the Hr motif (Fig. s2 in Additional file 1): absence of the conserved Trp in the pre-helix A and of the Asp in helix A, substitution of Asp for His in helix C, and of Glu for Asp in helix D. Of the WLV triplet in helix D, only the Leu residue (corresponding to L103 in the eukaryote sequences), which is known to play an important role in Hr function [23], is conserved. It remains to be determined whether the foregoing alterations compromise the structural or functional integrity of the fungal Hrs.

### Intron-exon structure of metazoan genes

**Figure 1:**
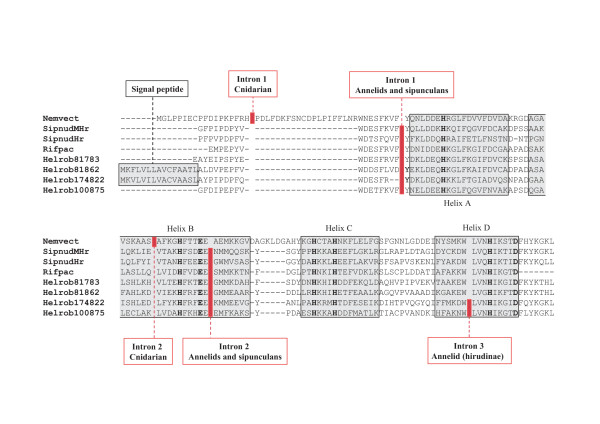
**The location of introns in the aligned Hr sequences from the cnidarian *N. vectensis *(jgi|Nemve1| 220584|fgenesh1_pg.scaffold_543000007; 136aa), the sipunculan *S. nudus *(Hr, CAG14943.1|GI:57282922; 119aa) and (MHr, CAG14944.1|GI:57282924; 119aa), the deep sea, hydrothermal vent vestimentiferan *R. pachyptila *(AM886446), and the leech *H. robusta *(jgi|Helro1|81783, 81862, 174822, 100875).** Eight of the 13 *H. robusta *sequences have atypical N-terminals, which appear to be signal peptide sequences. Note that the four *H. robusta *sequences shown represent all the observed combinations: no signal peptide and 2 introns (81783), a signal peptide and 2 introns (81862), a signal peptide and 3 introns (174822), and no signal peptide and 3 introns (100875). The seven residues involved in coordination with the two Fe are starred.

Since the intron-exon structure of Hr genes was unknown, we determined the locations of introns in Hr genes from the cnidarian *N. vectensis *(XP_001622541.1|GI:156351502), *R. pachyptila*, and *S. nudus*. An alignment of the sequences showing the different intron locations is given in Fig. 1. There are 2 introns in *S. nudus *Hr and MHr, the first one located just prior to helix A and the other at the end of helix B, both in phase 0. The annelid Hr genes have 2 or 3 introns: the locations of the first two introns are identical in the polychaete vestimentiferan *R. pachyptila *and the hirudinae *H. robusta*, and correspond to the locations in *S. nudus *Hr. A third intron (in phase 2) occurs in the middle of helix D in some members of the multigenic Hr family of *H. robusta *(Fig. 1). Although two introns are also found in the *N. vectensis *Hr gene, they occur at different locations (Fig. 1). No introns were found in the apicomplexan and protozoan Hrs.

### Signal peptide identification

SignalP 3.0 http://www.cbs.dtu.dk/services/SignalP was employed to locate probable signal peptide cleavage sites [24]. Of the 13 putative Hrs found in the genome of the leech *H. robusta*, 8 appear to have atypical N-terminals with a clearly identifiable signal peptide cleavage site (Fig. 1). All four possible combinations of 2 or 3 introns with and without signal peptides are observed: no signal peptide and 2 introns (jgi|Helro1|81783), a signal peptide and 2 introns (jgi|Helro1|81862, 81728, 81835, 174825, 100575), a signal peptide and 3 introns (jgi|Helro1|174822, 81819, 86578), and no signal peptide and 3 introns (jgi|Helro1|100875, 185740, 111854, 157306). The four possibilities are shown in Fig. 1.

### Prokaryote Hrs

Tables s2 and s3 and Fig. s2 in Additional file 1, list the putative archaeal and bacterial Hrs and show their alignments, respectively. A salient feature of prokaryotic Hrs is the presence of both single-domain Hrs and of chimeric proteins with N- and C-terminal domains. Of the 43 archaeal genomes, only 4 euryarchaeote genomes have 6 Hrs, one of them an N-terminal domain of a methyl accepting chemotaxis protein. Of the 444 bacterial genomes 118 (27%) have a total of 326 Hrs. Table 1 shows the distribution of single-domain and chimeric Hrs in the main bacterial groups that have Hrs: 242 (74%) are single-domain Hrs and 84 (26%) are domains in chimeric proteins. No Hrs were found in the genomes of Bacteroidetes/Chlorobi, Chlamydiae/Verrumicrobia, Chloroflexi, Deinococcus/Thermus, Fusobacteria, Nitrospirae and Thermotogales. The number of Hrs per genome varies widely, from 1 to as many as 31 in *Magnetospirillum magnetotacticum*. One of the ChHrs from *Magnetospirillum gryphiswaldense *(529aa, 197–329; CAJ30107|GI:78033490) has a central Hr domain. The remaining ChHrs vary in length from about 250 to over 1100aa: of these 30 (36%) have N-terminal, and 53 (64%) have C-terminal Hr domains. The alignments of the foregoing sequences in Fig. s2 of Additional file 1, show that 164 position are sufficient for the alignment of all Hr sequences, except for a couple with interhelical inserts. The 262aa Hr from the α-proteobacterium *Rhodospirillum rubrum *(YP_426610|GI:83592858) is unique in having two covalently linked Hr domains.

**Table 1:** Distribution of single domain and chimeric Hrs (> 250aa) in the main bacterial groups.

Taxon	Single domain Hrs	Total chimeric Hrs	Chimeric N-terminal	Chimeric C-terminal
Acidobacteria	8	-	-	-
Actinobacteria	2	-	-	-
Aquificiaea	2	-	-	-
Cyanobacteria	2	-	-	-
Firmicutes	25	-	-	-
Planctomycetes	1	1	1	-
α-Proteobacteria	61	26^1^	11	15
β-Proteobacteria	46	5	1	4
γ-Proteobacteria	23	22^2^	8	14
δ-Proteobacteria	43	16	4	12
ε-Proteobacteria	21	4	4	-
Unclassified proteobacteria	6	10^3^	2	8
Spirochaetes	2	-	-	-

Total	242	84	31	53

^1 ^*Magnetospirillum magneticum *and *M. magnetotacticum *account for 19.

^2 ^The Alteromonadales account for 16.

^3 ^*Magnetococcus sp. MC-1 *accounts for 9.

The nonHr domains of the ChHs are very variable, with about 20 still unidentified. Of the rest, GenBank identifies 32 as methyl accepting chemotactic proteins, followed by 16 GGDEF (metal-binding diguanylate cyclase) domains, 4 histidine kinase domains, 4 FOG:CheY-like domains, and 7 combinations of GGDEF domain, 6 with a PAS and one with an EAL domain. Examination of the O_2 _requirements of 97 Hr-containing bacteria in Table s3 (Additional file 1) did not reveal any correlation with Hr presence: only 9 were host associated.

### Altered Hr sequences

Table s4 in Additional file 1 lists the Hr sequences found to deviate from the canonical Hr sequence, either through alteration of one or more residues involved in iron coordination or loss of a helical segment: 58 out of the 327 bacterial sequences (18%) in 34 genomes, and one annelid. The alterations are listed in Table s5 in Additional file 1. Of the 59 deviant sequences, 11 have alterations in two helices and 4 lack a helical segment. The number of alterations in each of the four helices A, B, C and D, is 10, 11, 45 and 6, respectively. The overwhelming majority are substitutions of one of the 5 His residues whose side-chain groups coordinate the Fe atoms; only 5 alterations in the two acidic residues are evident. Most are found in helix C (45/71 = 63%), with several co-occurring with alterations in one other helix. The most common His substitutions are by Gln (24/71 = 34%), by a hydrophobic residue (A/V/L/I/M/Y) (19/71 = 27%), by Asn (7/71 = 10%) and by Glu/Asp (7/71 = 10%).

### Molecular phylogeny

**Figure 2:**
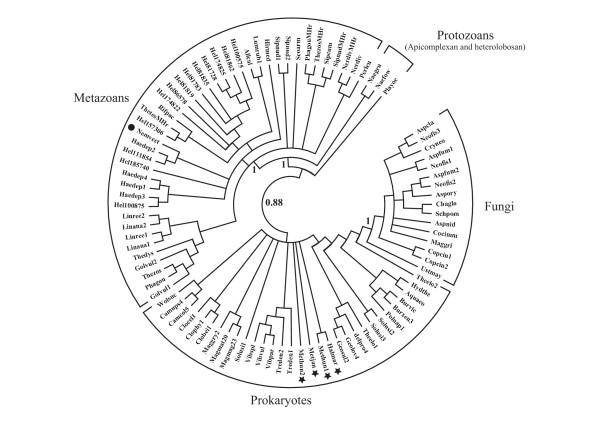
**A Bayesian phylogenetic tree of reduced set of Hr sequences, representing 31 bacterial, 16 fungal, 3 protozoan and 42 metazoan sequences.** The Hr sequences are identified by the fist three letter of the genus name and the first three letters of the species name (see Fig. s2 in Additional file 1). The archaeal Hrs are marked by a star and the *N. vectensis *Hr is marked with a dot.

A global Bayesian phylogenetic tree of 92 Hr sequences, comprising 42 metazoan, 3 protozoan, 16 fungal and 31 prokaryote Hrs, is shown in Fig. 2. Independent clusters are formed by the prokaryote and fungal Hrs on one hand, and the apicomplexan, heterolobosan and metazoan Hrs on the other, supported by a posterior probability of 0.88. In the prokaryote clade there is extensive polytomy which does not allow discrimination between archaea and bacteria. Furthermore, the putative fungal Hrs are closely clustered with the prokaryote Hrs with a posterior probability of 1. In the eukaryote branch, the apicomplexan (*Plasmodium yoelii*) and the heterolobosan (*N. fowleri *and *N. gruberi*) Hrs are basal to the protostome phyla, also with high posterior probabilities. The annelid, sipunculan, brachiopod and cnidarian (*N. vectensis*) Hrs are not resolved into individual clades. Furthermore, there is also a polytomy at the base of the metazoan clade, including the cnidarian Hr, expected to occur at the base of the Bilateria, together with several annelid Hrs. It should be pointed out that Bayesian phylogenetic trees constructed using subsets of the total number of Hr sequences, also gave topologies identical to that obtained above (see Figs. s3 and s4 in Additional file 1).

## Discussion

### Distribution and function in eukaryotes

The distribution of Hrs in eukaryotes is limited to fungi, the apicomplexan *Plasmodium yoelii*, the heterolobosan *Naegleria *and five metazoan phyla- the cnidarian *N. vectensis*, annelids and three minor phyla, the sipunculans, brachiopods and priapulids. The presence of Hrs in all three major annelid groups, the hirudinae, oligochaetes and polychaetes, suggests that they may be ubiquitous in Annelida. However, given their absence in the genome of the polychaete *Capitella sp.I*, the extent of Hr occurrence in annelids remains to be determined.

The intron-exon structures of the MHr and Hr genes of *S. nudus *suggest that they emerged via a duplication event. Although no oligochaete Hr gene structure is known to date, the identical polychaete and hirudinean intron locations supports the notion of a common Hr ancestor to the sipunculans and annelids [14]. The presence of a third intron in some members of the *H. robusta *multigenic Hr family suggests an intron gain during the emergence of this species. The presence of two introns in *N. vectensis *Hr, inserted in positions different from the other metazoan Hrs (Fig. 1), indicates a different evolution of Hr genes in the Radiata relative to the Bilateria. Overall, it appears that the Hr gene was lost in the ancestor to the deuterostomes and conserved only in a few protostomes after the Radiata-Bilateria transition and the protostome-deuterostome split. The unexpected identification of signal peptide cleavage sites in some Hrs from the leech *H. robusta *(Fig. 1), implies that these Hrs are directly released into coelomic or vascular compartments, similar to the extracellular annelid hemoglobins [25]: to our knowledge this is the first known instance of possible extracellular Hr location.

The Hrs in circulating, nucleated coelomocytes within the coelomic and tentacular fluid compartments and the cytoplasmic MHrs in Sipuncula, Brachiopoda, Priapulida and the polychaete *Magelona papillicornis*, have O_2 _binding properties consonant with physiological roles of O_2 _transport and storage [4,26]. Since annelids generally have intracellular or extracellular Hbs or both [27], their Hrs are likely to have functions other than O_2 _transport. The Hrs of the polychaete *N. diversicolor and the oligochaete A. caliginosa *have been proposed to function as scavengers of heavy metals, such as Cd [8,9,28] and an antibacterial function has been proposed for the former [29]. In the leech *Hirudo medicinalis*, Hr occurs in neural and other tissues and is upregulated in response to septic injury [12]. A Hr was identified as a major component of mature oocytes in the leech *T. tessulatum *[30]: its presence throughout oogenesis suggests a more complex function than just a nutrient for the embryo, perhaps in iron storage and detoxification. In the leech *H. medicinalis*, Hr plays a role in the innate immune response of the nervous system to bacterial invasion [11]. The binding of sulfide by the Hr in the hemolymph of the priapulid *Halicryptus spinulosus *[31], suggests a possible role in sulfide detoxification. Hrs are also antigenic [32]: the Hr in the amoeba *N. fowlerii *was discovered in a search for the antigen-related activity of this parasite [12].

### Distribution and function in prokaryotes

Our survey demonstrates the presence of putative Hrs in < 10% of archaeal genomes (4 out of 43) and in < 30% of bacterial genomes (118 out of 444). In Archaea, Hrs occurs only in one of the two major groups, the Euryarchaea, and only in the Halobacteria, Methanococci and Methanomicrobia. In Bacteria, about 80% of the genomes containing Hrs belong to the Proteobacteria. Furthermore, we find that one of 6 archaeal and about one fifth (18%) of the putative bacterial Hr sequences have one or more alterations potentially affecting the integrity of the diiron binding site. Although we do not know how many of the altered sequences listed in Table s4 in Additional file 1 retain their function, we are left with a very sparse and episodic distribution of Hrs among the prokaryotes, of which one fifth appear to have mutated away from the canonical Hr motif. The overwhelming majority of the altered sequences are single domain Hrs, implying that their function may be less important to the survival of the organism than the chimeric Hrs.

Karlsen et al. [17] have cloned the gene for a 131aa Hr from the methanotrophic γ-proteobacterium *M. capsulatus*, and found that its *in vivo *expression increased with increase in the copper content of the growth medium, implying a possible function as O_2_-provider to the O_2_-requiring, membrane-associated methane monooxygenase, the enzyme responsible for oxidizing methane in *M. capsulatus *grown at high copper concentrations. Although nothing is known about the role of other SDHrs in bacteria, the 959aa ChHr from the sulfate-reducing δ-ptoteobacterium *D. vulgaris*, has been shown to be a chemotactic protein with a C-terminal Hr domain [34]. Chemotactic proteins generally comprise a periplasmic N-terminal sensor domain, linked via a trans-membrane domain to a C-terminal cytoplasmic transmitter domain. A phosphorylation/methylation cascade triggered by an environmental stimulus is transduced from the sensor to the transmitter domain, resulting in an alteration of the flagellar motion, allowing movement up or down a concentration gradient of the stimulus [35,36]. *D. vulgaris *is microaerobic and prefers to swim to a specific O_2 _concentration range [37]. On the basis of a crystal structure of the expressed Hr domain of DcrH, and consistent with its cytoplasmic localization, Kurtz et al. [18] proposed that DcrH functions as an anaerotactic O_2 _sensor. There appear to be at least three more putative chimeric proteins with C-terminal Hr domains as well as two SDHrs in *D. vulgaris *(Table s3 in Additional file 1).

One final interesting observation resulting from our survey, is the presence of multiple SDHrs and ChHrs in the genomes of several magnetotactic bacteria, e.g. *Magnetococcus sp., Magnetospirillum magneticum *and *M. magnetotacticum*, with 14 (6SDHrs, 8ChHrs), 37 (27SDHrs, 10ChHrs) and 31 (22SDHrs, 9ChHrs) Hrs, respectively (Table s3 in Additional file 1), also observed earlier [33]. There are however, many magnetotactic bacteria which apparently do not have Hrs. Magnetotaxis, the ability to align and move along geomagnetic field lines, enables bacteria to be more efficient in locating a desired position in the vertical O_2_concentration gradient in their aquatic environments: it depends on the presence of specialized organelles, magnetosomes, comprised of Fe_3_O_4_/Fe_3_S_4 _crystals enclosed in a lipid bilayer membrane derived from the cytoplasmic membrane [38,39]. It remains to be determined whether Hrs have any role in magnetosome formation or function.

Overall our results are in agreement with the results of a very recent review of bacterial Hrs by French et al. [33], published while this manuscript was in preparation. These authors suggest that single domain Hrs may function in the delivery of O_2 _to oxygenases and respiratory oxidases, implied by the findings of Karlsen et al. [17] and consonant with the retention by the bacterial Hrs of the complete molecular signature of the O_2 _binfing Hrs in sipunculans and brachiopods.

### Molecular phylogeny and evolution of Hrs

**Figure 3:**
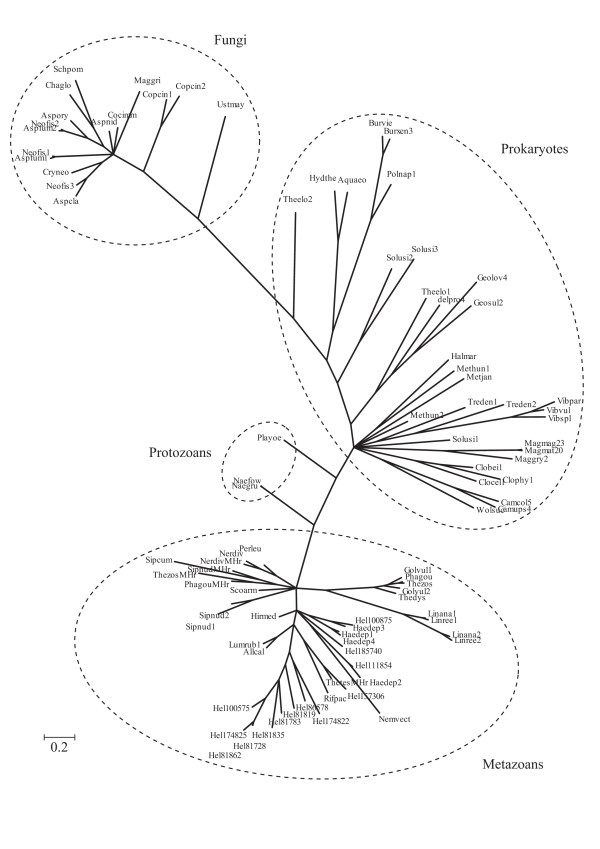
**Radial representation of the Bayesian phylogenetic tree of the reduced set of Hr sequences, comprising 31 bacterial, 16 fungal, 3 protozoan and 42 metazoan Hrs.** The Hr sequences are identified by the fist three letter of the genus name and the first three letters of the species name (see Fig. s2 in Additional file 1). The archaeal Hrs are marked by a star and the *N. vectensis *Hr is marked with a dot.

The global Bayesian phylogenetic tree shown in Fig. 2, shows that the Opisthokont (animal and fungal) Hrs do not cluster together, as would be expected according to the consensus phylogeny of Baldauf [40]. Furthermore, the metazoan Hrs group together with two evolutionarily distant groups, the Alveolates (Apicomplexa) and the Discicristates (Heterolobosa) [41]. The clustering of fungal Hrs with the bacterial sequences suggests the possibility of horizontal gene transfer from bacteria to fungi. Alternatively, the Long Branch attraction effect during the molecular phylogeny reconstruction process could have resulted in an artefactual clustering with bacteria [42]. The radial phylogenetic tree representation with distances provided in Fig. 3, clearly shows the long distance separating the fungal and prokaryote clusters.

It is plausible to assume that α-helical bundles were among the earliest protein folds to emerge since the beginning of life, well-adapted to the binding of metal ions and small organic molecules. Consequently, both Hrs and globins are two very ancient protein families, which emerged as adaptations to possible environmental challenges to the last universal common ancestor (LUCA) or populations of microbial organisms representing LUCA. These adaptations would include the need to sequester reduced iron, which was probably abundant on early Earth, the ability to control locally excessive O_2 _concentrations_, _which would have been lethal to anaerobic life, and the need to detoxify nitric oxide produced in O_2_-rich environments [43]. Another, equally plausible early function, would have been chemotactic sensing, enabling anaerobic organisms to avoid high O_2 _concentrations; both aerophilic and aerophobic responses would have survival value throughout bacterial evolution (K. Van Holde, personal communication). This alternative is supported by the presence of chemotactic Hr-containing proteins and of globin-coupled sensors capable of eliciting either an aerophilic or aerophobic response [44]. However, only 39 of 118 (33%) Hr-containing bacterial genomes have ChHrs (Table s4 in Additional file 1) and 93 of 264 (35%) globin-containing bacterial genomes have globin-coupled sensors [43]. Thus, in extant prokaryotes, chemotactic sensing appears not to be a major function in the two protein families; what then is the function of the single domain Hrs in prokaryotes? The similarity of the amino acid sequences of the prokaryote and metazoan Hrs indicates that O_2 _binding is likely to be involved in the function of the former, mentioned earlier [33].

Comparison of the phylogenomic profile of Hrs and globins (2), underscores the contrast in the evolutionary fates of the two protein families: presence in < 10% versus 25% of archaeal genomes, < 20% versus ~60% of bacterial genomes and ~13% versus > 80% of eukaryote genomes, respectively. In particular, the ~13% Hr presence in eukaryotes is greatly exaggerated because of the overrepresentation of fungi in the sequenced eukaryote genomes. Furthermore, unlike Hrs, globins are found in every major bacterial group, occur widely in eukaryotes and are ubiquitous among plants and vertebrates. Compared to globins, Hrs have barely maintained a foothold in living organisms, particularly multicellular ones. The apparent lack of evolutionary success of Hrs versus globins could be due to the greater probability of potentially damaging mutations in the former relative to the latter: seven residues binding the two Fe versus only the proximal His binding to the heme group. Alterations affecting one or more of the Fe-coordinating amino acid residues as well as the structure of the O_2_-binding cavity can be expected to have a direct deleterious effect on Hr function [45].

## Conclusion

A survey of putative Hrs demonstrated a limited occurrence in bacteria and archaea and a marked absence in the vast majority of multicellular organisms. Among the metazoa, Hrs have survived in a cnidarian and in a few protostome groups; hence, it appears that in metazoans the Hr gene was lost in deuterostome ancestor(s) after the radiata/bilateria split. Signal peptide sequences in several Hirudinea Hrs suggest for the first time, the possibility of extracellular localization. Since the α-helical bundle is likely to have been among the earliest protein folds, Hrs represent an ancient family of iron-binding proteins, whose primary function in bacteria may have been that of an oxygen sensor, enabling aerophilic or aerophobic responses. Although Hrs evolved to function as O_2 _transporters in brachiopods, priapulids and sipunculans, their function in annelids remains to be elucidated. Overall Hrs exhibit a considerable lack of evolutionary success in metazoans.

## Methods

### Identification of Hr squences

Two approaches were used to identify putative Hrs in the genomes of 37 archaea, 440 bacteria and 135 eukaryotes. In one, we examined the gene assignments based on a library of hidden Markov models [46], listed on the SUPERFAMILY site http://supfam.mrc-lmb.cam.ac.uk, discarding sequences shorter than 100aa. In the other, we performed blastp and tblastn (version 9.2.2) and psiblast searches, using the improved version with composition based statistics [47], of completed and unfinished genomes in the GenBank http://www.ncbi.nlm.nih.gov/BLAST/. In cases of borderline sequences, searches employing PFAM [48]http://pfam.sanger.ac.uk and FUGUE [22]http://tardis.nibio.go.jp/fugue were used to determine whether they should be accepted as a Hr.

### Alignment of Hr sequences

The sequences were aligned using MUSCLE [49] and MAFFT [50], with an iterative refinement option incorporating local pairwise alignment information http://www.biophys.kyoto-u.ac.jp/, and manually, using the conserved Hr motif generated by the structural alignment employing MUSTANG [51] and shown in Fig. s1 in Additional file 1: -W-12X-D-2X-H-K-X-L-F/V-<variable>-L-6X-H-F-2X-E-2X-L-M-<variable>-HK-2X-H-F-I/L/V-<variable>-WLV-X-H-I-3X-D-2X-Y-3X-L/V.

### Biological Material

Specimens of the hydrothermal vent tube worm, *R. pachyptila*, were collected on the EPR (9_50¡N at the Riftia Field site) at a depth of about 2500 m, during the French oceanographic cruise HOT 96 and the American cruise LARVE'99. The worms were sampled using the telemanipulated arms of the submersibles Nautile and Alvin, brought back alive to the surface inside a temperature-insulated basket, and immediately frozen and stored in liquid nitrogen after their recovery on board. Live specimens of the polychaete *Scoloplos armiger *were collected at the Station Biologique de Roscoff (France) and stored in liquid nitrogen. Coelomic erythrocytes from *Sipunculus nudus *were isolated from living worms provided from the Station Biologique de Roscoff (France).

### Total RNA Extraction and cDNA Synthesis

Erythrocytes from coelomic fluids of *S. nudus *were separated by centrifugation for 5 min at 2000 g and homogenized in liquid nitrogen. Total RNA was extracted using Trizol Reagent (Gibco). Reverse transcription was initiated directly on total RNA, without further purification, with the oligo dT CTC CTC TCC TCT CCT CTT recommended by the Promega reverse transcriptase kit protocol. Moreover, a pool of total RNA was extracted from the intestinal tube tissue of *S. nudus *to synthesize a second cDNA template.

### Hr Primer Design

Degenerate forward and reverse Hr-specific primers were designed according to an amino acid sequence multiple alignment obtained from the Hr sequences available in the Swiss-Prot database: *Phascolopsis gouldii *(P02244), *Themiste zostericola *(P02245), *T. dyscriptum *(P02246), and *Siphonosoma cumanense *(P22766). The following two primers–HR3A, 5'-DAT YTT NCC YTT RTA YTT RAA RTC-3' (forward), and HR5A, 5'-GGN TTY CCN ATD CCN GAY CC-3' (reverse) (MGW Biotech)–were then used for PCRs using a cDNA template.

### Hr Amplification and Sequencing

Each partial myoHr or Hr cDNA was amplified by PCR using a Perkin-Elmer GenAmp PCR System 2400. PCR were carried out as follows: initial denaturation at 96°C for 5 min, then 35 cycles consisting of 96°C for 50s, 50°C for 50s, and 72°C for 50s. The reaction was completed by an elongation step of 10 min at 72°C.

Amplifications were carried out in 25 μl reaction mixtures containing 10–50 ng of cDNA target, 50–100 ng of each degenerate primer, 200 μM dNTPs, 2.5 mM MgCl_2_, and 1 unit of TaqDNA polymerase (Promega). PCR products were visualized on a 1% agarose (Eurobio) gel under UV radiation. Gel slices containing DNA fragments of the expected size (~200 bp) were collected and subsequently purified onto Ultrafree-DA (Millipore). PCR products were then cloned using a TOPO-TA Cloning Kit (Invitrogen). Purified plasmids containing the Hr insert were sent to the Biotechnology Center CRIBI (University of Padua, Italy) for sequencing. The 3' and 5' end coding sequences were obtained by RACE 5'/3' (Roche) following the protocols provided with the kit.

### Molecular Phylogenetic Analysis

Bayesian phylogenetic trees were obtained using MrBayes Version 3.1.2 (52); four chains were run simultaneously for 3 × 10^6 ^generations and trees were sampled every 100 generations. The Jones transition matrix (53) was selected and used as the model of amino acid substitution. The final average standard deviation of split frequencies was 0.013.

## Abbreviations

Hr: hemerythrin; MHr: Hr present in muscle tissue; SDHr: single domain Hr: < 250aa; ChHr: chimeric protein with an N-terminal or C-terminal Hr domain; Hb: hemoglobin.

## Authors' contributions

XB constructed the phylogenetic trees. SNV searched for Hr sequences and SNV and KM performed the alignments. SV, CC and KM participated in the analysis and interpretation of the data. XB and SNV drafted the manuscripts and SV, CC and KM revised it critically. All the authors read and approved the version to be published

## Supplementary Material

Additional File 1 **Supplementary material**. Fig. s1. Structural alignment of the four known Hr crystal structures (with the four alpha-helical segments A-D in red) obtained using MUSTANG (31), the derived Hr fold highlighting the conservation of the Fe-coordinating and several hydrophobic residues (numbered using the *Themiste dyscritum *Hr). Fig. s2. Alignment of Hr sequences listed in Tables s1–s3 in Additional file 1, in the following order: eukaryotes, archaea and bacteria. Note that the last two bacterial sequences have uncommon interhelical inserts. Fig. s3. A Bayesian phylogenetic tree of 3 protozoan and 42 metazoan sequences. The Hr sequences are identified by the fist three letter of the genus name and the first three letters of the species name (see Fig. s2 in Additional file 1). The cnidarian *N. vectensis *Hr is marked with a dot. Fig. s4. A Bayesian phylogenetic tree of 16 fungal, 3 protozoan and 42 metazoan sequences. The Hr sequences are identified by the fist three letter of the genus name and the first three letters of the species name (see Fig. s2 in Additional file 1). The cnidarian *N. vectensis *Hr is marked with a dot. Table s1. Putative hemerythrins in eukaryote genomes. Table s2. Putative hemerythrins in archaeal genomes. Table s3. Identified and putative hemerythrins in bacteria. Table s4. Hemerythrin sequences with alterations at the seven iron-coordinating positions. Table s5. Numbers of alterations to the His resIdues coordinating the two Fe atoms in the four helices of bacterial Hrs. Click here for file
