# Supplementary material for: A phylogenomic profile of hemerythrins, the nonheme diiron binding respiratory proteins
Source: BMC Evol Biol. 2008 Sep 2;8:244. doi: 10.1186/1471-2148-8-244 (PMC2556348; doi:10.1186/1471-2148-8-244)
Supplement: Additional File 1 — Supplementary material. Fig. s1. Structural alignment of the four known Hr crystal structures (with the four alpha-helical segments A-D in red) obtained using MUSTANG (31), the derived Hr fold highlighting the conservation of the Fe-coordinating and several hydrophobic residues (numbered using the Themiste dyscritum Hr). Fig. s2. Alignment of Hr sequences listed in Tables s1–s3 in Additional file 1, in the following order: eukaryotes, archaea and bacteria. Note that the last two bacterial sequences have uncommon interhelical inserts. Fig. s3. A Bayesian phylogenetic tree of 3 protozoan and 42 metazoan sequences. The Hr sequences are identified by the fist three letter of the genus name and the first three letters of the species name (see Fig. s2 in Additional file 1). The cnidarian N. vectensis Hr is marked with a dot. Fig. s4. A Bayesian phylogenetic tree of 16 fungal, 3 protozoan and 42 metazoan sequences. The Hr sequences are identified by the fist three letter of the genus name and the first three letters of the species name (see Fig. s2 in Additional file 1). The cnidarian N. vectensis Hr is marked with a dot. Table s1. Putative hemerythrins in eukaryote genomes. Table s2. Putative hemerythrins in archaeal genomes. Table s3. Identified and putative hemerythrins in bacteria. Table s4. Hemerythrin sequences with alterations at the seven iron-coordinating positions. Table s5. Numbers of alterations to the His resIdues coordinating the two Fe atoms in the four helices of bacterial Hrs. [file 1471-2148-8-244-S1.pdf]

ADDITIONAL MATERIAL

Fig. 1. Structural alignment of the four known Hr crystal structures (with the four alpha-helical segments A-D in red) obtained using MUSTANG (31), the derived Hr fold highlighting the conservation of the Fe-coordinating and several hydrophobic residues (numbered using the *Themiste dyscritum* Hr).

```
2awc Desulfovibrio vulgaris DcrH
-------VKWSEDLAN-LPSI-DTQHKRLVDYINDLYRAARRR------DMDKAREVFDALKNYAVEHFGYEERLFADYA--Y----------PEATRHKEIHRRFVETVLKWEKQLAAG---------DPEVVMTTLRGLVDWLVNHIMKEDKKYEAYLRERGVS
1i4y Phascolopsis gouldii Hr
GFPIPDPYVWDPSFRTFYSII-DDEHKTLFNGIFHLAID-------------DNADNLGELRRCTGKHFLNEQVLMQASQ--Y----------QFYDEHKKEHETFIHALDNWK--------------------G-DVKWAKSWLVNHIKTIDFKYKGKI
2mhr Themiste zostericola MHr
GWEIPEPYVWDESFRVFYEQL-DEEHKKIFKGIFDCIRD-------------NSAPNLATLVKVTTNHFTHEEAMMDAAK--Y----------SEVVPHKKMHKDFLEKIGGLS---APV--------------DAKNVDYCKEWLVNHIKGTDFKYKGKL
1hmd Themiste dyscritum Hr
GFPIPDPYCWDISFRTFYTIVIDDEHKTLFNGILLLSQA-------------DNADHLNELRRCTGKHFLNEQQLMQASQ--Y----------AGYAEHKKAHDDFIHKLDTWD--------------------G-DVTYAKNWLVNHIKTIDFKYRGKI
Hr fold--W------------D--HK-LV------------------------------L------HF--E--LF----------------------HK--H--FI------------------------------------WLV-HI---D--Y---L
----------------------════ HELIX A ════---------------════════ HELIX B ════════------------------════ HELIX C ════---------------------------═══ HELIX D ═══--------
```

Iron coordination: Fe1 - H74, H78, H102; E58, D106; Fe2 (O2 binding site) - H26, H55; E59, D107.

Fig. 2. Alignment of Hr sequences listed in Tables 1-3 in Supplementary Material, in the following order: eukaryotes, archaea and bacteria. Note that the last two bacterial sequences have uncommon interhelical inserts.

```
ALVEOLATA
>Plasmodium yoelii yoelii (Alveolata; Apicomplexa) gb|AABL01004457.1 107aa
----------------------DDQHKTIFDMVNKLHDT------AATGNRSEIGKQLDALIDYVVMHFKSEETEMQKKG--Y----------ADFAAHKAEHDKLVGVCADLQKKFHAG---------EAEVNQDTTRFVRDWLVNHIPKVDKLY----
---------W------------D--HK-LV------------------------------L------HF--E--LF----------------------HK--H--FI------------------------------------WLV-HI---D--Y---L

ANNELIDA
>Haementeria depressa (Annelida; Hirudinae) gi|47833904|gb|CN807658.1 119aa
GFEIPEPFCWDESFKV-FYEQIDQEHKGLFDAIFKLAKS------------PSDCGALSHLAKLIDEHFAFEEGMMKKAN--Y----------DDFENHHKAHVDFVGTLKTVS---CPV-------------AADKLHWAKEWLVNHIKTTDFKYKGKL
---------W------------D--HK-LV------------------------------L------HF--E--LF----------------------HK--H--FI------------------------------------WLV-HI---D--Y---L
>Helobdella robusta (Annelida; Hirudinae) jgi|Helro1|157306 120aa
GFDIPEPFQWDESFLVSYDKL-DDEHKGLFQGIKSVSES------------PSCSETLEKLVQLIKQHFLDEEEMMKAKG--F----------DDIEAHSKAHTDFVETLKGVQ---TPV-------------TAENIKMAKEWLVNHIKGTDFKYKGKL
>Helobdella robusta (Annelida; Hirudinae) jgi|Helro1|100875 120aa
GFDIPEPFVWDETFKVFYNEL-DEEHKGLFQGVFNV------------AKAPSDQGALECLAKLVDAHFKHEEEMFKAKS--Y----------PDAESHKKAHDDFMATLKTIA---CPV-------------ANDKIHFAKNWLVNHIKGTDFLYKGKL
>Helobdella robusta (Annelida; Hirudinae) jgi|Helro1|81783 121aa
AYEIPSPYEWDESFRVFYDKL-DEQHKGLFKGIFDV------------CKSPSDASALSHLKHVLTEHFKTEEGMMKDAH--F----------SDYDNHKHIHDDFEKQLDAQH---VPI-------------PVEKVTAAKEWLVNHIKGIDFKYKTHL
>Helobdella robusta (Annelida; Hirudinae) jgi|Helro1|185740 123aa
GFAIPEPYIWDESFRVFYDTL-DEQHKNLFHVIFSL------------AKSPDDPIVLKHLVKTIDEHFDHEEGLLRKAN--Y----------ADYDVHHQEHLNFQKALKKVR---TPV-------------SDEQITWAKDWLVNHIKGTDFQYKGKL
>Helobdella robusta (Annelida; Hirudinae) jgi|Helro1|111854 132aa
SHPIPEPFVWDESFKVFYDKL-DGEHQGLFQAIFDV------------GKTPNDSGVLAKLVELMDSHFAHEEREMKKAA--F----------DGHDEHKDNHDEFLASLKKLK---TPV-----------AQESVQWAKNWLVHHIKVTDSKYSGKLHL
>Helobdella robusta (Annelida; Hirudinae) jgi|Helro1|86578 136aa
ASDIPSPFQWNESFRVSYDTL-DEQHKGLFKGIYDV------------EQSPGSSQALTNLRNVIETHFRTEEKMMKDAG--Y----------SEYDSHRKIHTDFEKDLSGYT---TTV-----------SSNQIQTAKEWLVNHIKGIDFKYKGKLSN
>Helobdella robusta (Annelida; Hirudinae) jgi|Helro1|100575 134aa
AFDIPEPYKWDESFKVDYEKL-DEQHKGLFQAIFDV------------CAAPADAAALAHLKDVIEKHFRFEEGMMTAAD--Y----------AGYADHKKAHDGFEARLKEGQ---VPV-------------AADDVKAAKEWLVNHIKGVDFKYKTLL
>Helobdella robusta (Annelida; Hirudinae) jgi|Helro1|81819 134aa
AFDIPRPFKWDESFKVFYEKI-DEQHQGLFEAIYQV------------CRHPEDAEKLKHLDDVIEKHFRDEEKMMEDAK--Y----------EEYDEHHKIHADFEAKLDAVH---VPV-------------TRDQVKYAKEWLVNHIKGIDFKYKGKL
>Helobdella robusta (Annelida; Hirudinae) jgi|Helro1|174825 134aa
ALDVPEPFVWDDSFLVDYEKL-DEQHKGLFKAIFDV------------CAAPADASAFAHLKDVIEKHFRFEEGMMEAAH--Y----------GDLPLHKQIHESFEAALKAGQ---VPV-------------AAADIKGAKEWLVGHIKTVDFKYKTHL
>Helobdella robusta (Annelida; Hirudinae) jgi|Helro1|174822 134aa
AYPIPDPFQWDESF--RYDKL-DEQHKKLFTGIADV------------QSNPADGAAISHLEDLFYKHFRFEEKMMEEVG--Y----------ANLPAHKRMHTDFESEIKDIH---TPV-------------QGYQIFFMKDWLVNHIKGIDFQYKGKL
>Helobdella robusta (Annelida; Hirudinae) jgi|Helro1|81862 134aa
ALDVPEPFVWDDSFLVDYEKL-DEQHKGLFKAIFDV------------CADPADASAFAHLKDVIEKHFRFEEGMMEAAR--Y----------DDLLRHKHIHETFEAALKAGQ---VPV-------------AAADIKGAKEWLVGHIKFTDFKYKTHL
>Helobdella robusta (Annelida; Hirudinae) jgi|Helro1|81835 136aa
AEPIPAPFKWNESFRVFYDKL-DEQHKGLFKAIADV------------EQHPSQAVAYAHLKSVMGKHFRFEEDMMKEAQ--F----------ADLATHKGIHEDFEATLRASQ---VPI-------------SSEKVTGAKQWLVDHIKLVDFKYKGKL
>Helobdella robusta (Annelida; Hirudinae) jgi|Helro1|175368 165aa
RSHKVTDYTYEQICSNRYPKL-DDQHQQVFSEVDKL-----------SENTGDEAQIKYTI-SIYKMHFDYEESQMRKAN--Y----------ENYDDHKLLHESFLEEFRNHP---IPI-------------SIPNMEKIKSWLIAHIKTYDRKFNGKM
>Hirudo medicinalis (Annelida; Hirudinae) 120aa AAS49167.1  GI:44921619
GFEIPEPYVWDESFKV-FYENLDEEHKGLFQAIFNLSKS------------PADNGALKHLSKVIDEHFSHEEDMMKKAS--Y----------SDFDNHKKAHVDFQASLGGLS---SPV-------------ANDKVHWAKDWLVNHIKGTDFLYKGKL
>Theromyzon tessulatum MHr (Annelida; Hirudinae) 120aa Q9GYZ9  GI:21263690
VFEIPEPYQWDETFEV-FYEKLDEEHKGLFKGIKDLS-D-----------SPACSETLEKLVKLIEDHFTDEEEMMKSKS--Y----------EDLDSHKKIHSDFVETLKGVK---APV-------------SEENIKMAKEWLVNHIKGTDFKYKGKL
---------W------------D--HK-LV------------------------------L------HF--E--LF----------------------HK--H--FI------------------------------------WLV-HI---D--Y---L
>Allolobophora caliginosa (Annelida; Oligochaeta) 119aa
GFDIPEPFVWDESFKV-FYDLLDDEHKGLFKGIFDVAKA------------PGDGGALDHLRQVVSKHFSDEEGMMEKAN--Y----------AEVGPHKQMHTDFVAKLKGLS---TPV-------------SNDTIHFAKDDLVNHIKTTDFKYKGKL
>Neanthes (Nereis) diversicolor Hr (Annelida; Polychaeta) 119aa P80255  GI:417311
GFEIPEPYKWDESFQV-FYEKLDEEHKQIFNAIFALCGG-------------NNAGNLKSLVDVTANHFADEEAMLKASAS-Y----------GDFDSHKKKHEDFLAVIRGLG---APV-------------PQDKINYAKEWLVNHIKGTDFGYKGKL
>Neanthes (Nereis) diversicolor MHr (Annelida; Polychaeta) 120aa P22761  GI:123030
GFEVPEPFKWDESFQV-FYDKLDEEHKQIFNAIFALGGG-------------NNADNLKKMIDVTANHFADEEAMMLASAA-Y---------KSEHPGHKKKHEDFLAVIRGLS---APV-------------PNDKLLYAKDWLVNHIKGTDFTYKGKL
>Periserrula leucophryna (Metazoa; Annelida; Polychaeta) 119aa AAQ63634.1  GI:34327785
GFDIPEPFKWDASFEV-FYKKLDDEHKAIFDSIFAVCNG-------------NNADNLKKMVDVTANHFSDEEGMMKSAN--Y----------ADFDSHKKKHEDFLVVIRGLS---APV-------------GDDKLHYAKDWLVNHIKGIDFQYKGKL
>Riftia pachyptila (Annelida; Vestimentifera) 110aa
--EMPEPYVWDESFRV-FYDNIDDEHKGLFKGIFDCGAA------------PADSAKLASLLQLVIDHFVDEESMMKKTN--F----------DGLPTHNKIHTEFVDKLKSLS---CPL-------------DDATIAFAKKWLVNHIKGTD-------
>Scoloplos armiger (Annelida; Polychaeta) 110aa
--DVPEPYVWDESFKV-FYANLDEEHKGLFQGVFNVAGD------------KGSADKLSALVAAVDKHFKNEEGMMQAKS--Y----------ADFPAHKQAHEAFLGKIKGLK---APV-------------DDATVHFAKDWLVNHMKGTD-------
---------W------------D--HK-LV------------------------------L------HF--E--LF----------------------HK--H--FI------------------------------------WLV-HI---D--Y---L

BRACHIOPODA
>Lingula anatina HrA (Brachiopoda) 117aa P22764  GI:123038
-VKVPEPFAWNESFAT-SYKNIDLEHRTLFNGLFALSEF-------------NTRDQLLACKEVFVMHFRDEQGQMEKAN--Y----------EHFEEHRGIHEGFLEKMGHWK---APV-------------AQKDIKFGMEWLVNHIPTEDFKYKGKL
>Lingula anatina HrB (Brachiopoda) 117aa P22765  GI:123044
-MKIPVPYAWTPDFKT-TYENIDSEHRTLFNGLFALSEF-------------NTQHQLNAAIEVFTLHFHDEQGQMIRSN--Y----------VNTKEHTDIHNGFMDTMRGWQ---SPV-------------PQKALKDGMEWLANHIPTEDFKYKGKL
>Lingula reevii HrA (Brachiopoda) 117aa P23543  GI:46397821
-VKVPAPFAWNEDFAT-SYKFIDLEHRTLFNGLFALSEF-------------NTRDQLLACKEVFVMHFRDEQGQMEKAN--Y----------EHFEEHKGIHEGFLEKMGHWK---APV-------------AQKDIRFGMEWLVNHIPAEDFKYKGKL
>Lingula reevii HrB (Brachiopoda) 117aa P23544  GI:46397822
-MKVPAPYAWNSDFAT-TYENIDSEHRTLFNGLFALAEF-------------NTLTQLNAAIEVFTLHFHDEQGQMIRSN--Y----------VNTKEHTDIHNGFMDVMRGWR---SPV-------------PQQDLLAGMAWLANHIPTEDFKYKGKL
---------W------------D--HK-LV------------------------------L------HF--E--LF----------------------HK--H--FI------------------------------------WLV-HI---D--Y---L

CNIDARIA
>Nematostella vectensis XP_001634535.1  GI:156388091 119aa
--------RWNESFKVFYQNLDD-EHRGLFDVVFDV------------DAKRGDAGAVSKAASAFKGHFTTEEAEMKKGVDAGKLDGAHYKGHCTAHNKFLELF---GSFGNNLGDDEINYSMKWLVNHIKSTDFHYKGKLIQ
>Nematostella vectensis XP_001622541.1  GI:156351502 156aa
PLPIFFLNRWNESFKVFYQNLDD-EHRGLFDVVFDV------------DAKRGDAGAVSKAASAFKGHFTTEEAEMKKGVDAGKLDGAHYKGHCTAHNKFLELF---GSFGNNLGDDEINYSMKWLVNHIKSTDFHYKGKLIQ
---------W------------D--HK-LV------------------------------L------HF--E--LF----------------------HK--H--FI------------------------------------WLV-HI---D--Y---L

FUNGI
>Ajellomyces capsulatus (Ascomycota; Pezizomycotina) 205aa (12-146) XP_001539786.1  GI:154277908
LQFIYLSHTTPVAMVSISDSVTK-DHKELEAFHNQIVNA---------DNDIIKTRFQNQFVWELARHSAAEELLLYPA---L---EKHLADGKERVEKDRERHQKTKEQLMEFQSMKPTDA----------NFEPTVNSLMDNLSAHMRDEEGDNLPAL
---------W------------D--HK-LV------------------------------L------HF--E--LF----------------------HK--H--FI------------------------------------WLV-HI---D--Y---L
>Aspergillus clavatus (Ascomycota; Pezizomycotina) 220aa (20-154) XP_001270510.1  GI:121704493
SLPIARAVSTTPIMTGRITDTVKQDHRQLEACYDKIMHA---------TDRDDQIRFQNLFTWELARHSIGEELVVYPA---F---EKHLPDGVSMADKDRREHQTVKEKLKKFQNLDPSNP----------EFMSTIKSLMVDLAQHIKEEETNDLVKL
>Aspergillus fumigatus (Ascomycota; Pezizomycotina ) 221aa (26-160)  XP_754309.1  GI:70999175
SHQVQAFTDWSQARRFRILDTIKHDHSEIKSFYELIVSS---------PGPEEQTKYQNQFTWELARHTVGEELVIYPA---L---EKYLDDGKELARKDRAEHQTVKEKLKAFQDMKSTDP----------RFIPTLQSLWDDLQEHIRHEETEDIQLL
>Aspergillus fumigatus (Ascomycota; Pezizomycotina; Eurotiomycetes) 227aa (25-159) XP_747053.1  GI:70983051
TLIQNRTFAITSPTMTRLADAIKDDHRELEEAYNKILSA---------KTSDEKTRWQNQFTWELARHSIGEELIVYPL---M---EKNLPNGKEMADKDRAEHQIVKEHLYKFQSLSSEDA----------EFEPTLKSLWSNLGQHIKEEESDDLPSL
>Aspergillus nidulans (Ascomycota; Pezizomycotina; Eurotiomycetes) 228aa (24-158) XP_681907.1  GI:67903302
TPLQQRFISQSPIAMTRIIDAIKQDHREIEDYYNKILSA---------TTEKEKIQWQNQFTWELARHSIGEELVVYPV---F---EKNLPDGRAMADKDRHEHNTVKEKLKQFQNMKPSDP----------NFEPTLKSLMSTLSEHIQEEESQDLPKL
>Aspergillus oryzae (Ascomycota; Pezizomycotina) 221aa (23-157) BAE63202.1  GI:83773074
TLGASRGFRTTAPAATRVSEVIKNDHRELEDQYNRILSA---------KTKDEKEQWQNQFTWELARHSIGEELVVYPR---M---EKVLDNGKTMADHDRHEHQIVKEDLYKFQGLQPDDP----------EFIPTLKTLWANLAQHIKEEETQDLPAL
>Chaetomium globosum (Ascomycota; Pezizomycotina) 197aa (1-126) XP_001219252.1  GI:116178806
---------MAASAATRVSERIKHDHDELREYYNNIKNA---------QRDDDKVKWQNQFVWELARHSVAEEIVVYPA---F---EKHIPNGMVMAEKDRSEHQQVKEKLHTFQSLAPTDP----------TFHSTIDSLWEALAQHMQEEERDDLPAL
>Coccidioides immitis (Ascomycota; Pezizomycotina) 181aa (1-121) XP_001245291.1  GI:119189369
---------------MGLSDSVKKDHRELEDYYNQIINA---------TDDDTKIRYRNQFTWELARHSIGEELLVYPA---F--EKHLHLDGKKKADRDRQEHQVVKEQLKQFQNMEPTEP----------NFIPTIKSLMSNLSEHIKEEEAEDLPAL
>Coprinopsis cinerea okayama (Basidiomycota; Hymenomycetes) 208aa (1-130) EAU86662.1  GI:116503767
-------MTSNPSAQHTLLQIITSDHRDIEAFYNEYLKNA--------GNKDGQTRWANQLTWTIARHSISEELVVYPQ---F--EKHLGDKGRAMAEQDRQDHQFVKDRLKLLEDMEVGSS----------QYDATLKEIMTELKKHMASEETEDLPLL
>Coprinopsis cinerea okayama (Basidiomycota; Hymenomycetes) 228aa (17-153) EAU89470.1  GI:116506575
RYPLARTLATTAPGHVRLLQAITDDHREIEAFYDQYLKNG--------GNTDAQQRWANQLTWEIARHSISEELVVYPL---L--EKVMADQGHVLAESDRDDHQFVKDRLKKLESIEVGTT----------AYNAILKDIMDHLKEHMRKEETEQFPQL
>Cryptococcus neoformans ( Basidiomycota; Hymenomycetes) 220aa (15-159)  XP_566714.1  GI:58258603
TRFFASPTTSSMAGNTSVADAVKHDHRELEEYYDQIIKA---------TDADTKTRYQNLFTWELARHSVGEELVVYPA---L---EKYVDGGKALADRDREEHQKVKEMLYKFQQLSPSDS----------EFEPTIKKLMSDLAEHIKEEETEDLVKL
>Magnaporthe grisea (Ascomycota; Pezizomycotina) 239aa (34-169) XP_360008.1  GI:39940942
QRQFASASASAGSGFVKISDAIKKDHQELEQYYNEVINS---------TDQEHQQKFGNQFTWELARHSVAEELLVYPA---F---ETHLGEEGRQMAEDDRKQHHEVKILLKEFQNMRSNDS---------EYVPKLKELWAKLSLHIQEEESHDMPAL
>Neosartorya fischeri (Ascomycota; Pezizomycotina) 221aa (25-160) XP_001263153.1 GI:119490985
SHQVQAFTDWSQARRFRILDTIKHDHREIKSFYELIVSS---------TDPEEQTKYQNQFTWELARHAVGEELVVYPA---L---EKYLDDGKELARKDRAEHQTVKEKLKAFQDMKSTDP----------RFIPTLQSLWDDLQEHIHHEETEDIQLL
>Neosartorya fischeri (Ascomycota; Pezizomycotina; Eurotiomycetes) 227aa (25-159) XP_001261781.1  GI:119483756
KLIQNRTFAITSPTMTRLADAIKDDHRELEEAYNKILSA---------KTSDEKTRWQNQFTWELARHSIGEELIVYPL---M---EKNLPNGKEMADKDRAEHQIVKEHLYKFQGLSPEDP----------EFEPTLKSLWNNLGQHIKEEESEDLPSL
>Neosartorya fischeri (Ascomycota; Pezizomycotina) 233aa (24-158)  XP_001267572.1  GI:119501631
PIAARSVSTSPIQMIGRITDTVKQDHREIEACYQRIINA---------KDRDEQTRYQNLFTWELARHSIGEELVVYPA---F----EKHLPDGISMAEKDRREHQTVKEKLKKFQNLDPSNA---------DFIPTLKSLMGDLAEHIREEETNDLVKL
>Schizosaccharomyces pombe (Ascomycota; Schizosaccharomycetes) 203aa (9-144)  NP_595013.1  GI:19115925
VSLNFFPVRLLSYKMTRISDAIFKDHRKLQSDYQNIKSA---------NDYDTATRWQNQFVWELARHSVGEEIVVYPK---F--EKYLGEEGKEMAEKDRHEHQLVKEMLYKFQSMKANQS----------NFIPALDELMESLQKHIDEEEQHDIPFL
>Ustilago maydis (Basidiomycota; Ustilaginomycetes) 244aa (32-170) XP_757573.1  GI:71005814
ISTNFAAAAFAMTGKD-LIKEIKVDHDNIRDLLKRFNDA------HKAGDQDLCEALVNTIIHEAAVHSDAEELSVYKL---M-----EKLHMTQVAEKDRQDHQNVKQAMAELDTNQISDA-------GLDTFAHKVDKACKLFLEHAEEEENDQLPQL
---------W------------D--HK-LV------------------------------L------HF--E---F----------------------HK--H--FI------------------------------------WLV-HI---D--Y---L

HETEROLOBOSEA
>Naegleria fowleri (Heterolobosea; Schizopyrenida) 119aa Q9NH76  GI:21263696
```

```
ATTIPSPFNWDSSFCVGNNELNE-QHKKLFALINAL-------------DANRSSASALKELLDFVVMHFKAEEDLFAKVN-F-SDSTSHKETHDKFVQDALGLK---T-V----GDAEIQFIKQWLVNHIKGSDMKYKGVL
---------W------------D--HK-LV-------------------------------L------HF--E--LF-----------------------HK--H--FI-----------------------------------------WLV-HI---D--Y---L
>Naegleria gruberi jgi|Naegr1|44819|fgeneshHS_pm.scaffold_69000005 119aa
SYPIPTPYEWSTTLDIGNAELNE-QHKKLFTLINAL-------------DAKRSCGTTLKELLDYVVFHFKTEEDLFAKAG-W-SESASHKETHDKFVQDALAL----TSV----GDAEMQFIKQWLITHICASDMKYKGVI
>Naegleria gruberi jgi|Naegr1|36233|estExt_gwp_gw1.C_160163 119aa
SFPIPSPYQYDDSFCVGNAEINE-QHKKLFELINAL-------------DANRTCGTTLKTLLDYVVMHFKTEEDLFAKHA-W-SESAAHKEIHDKFVQDALGL----TSV----GDAEMQFIRQWLVDHIMGSDMKYKGVI
---------W------------D--HK-LV-------------------------------L------HF--E--LF-----------------------HK--H--FI-----------------------------------------WLV-HI---D--Y---L

SIPUNCULA
>Golfingia vulgaris Hr1 (Sipuncula) 114aa CAG14945.1  GI:57282926
GFPIPDPYVWDPSFRT-FYTAIDDEHKTLFDGIFHLARD--------------DNKDNLGELRRCTGKHFLNEQVMMQASQ--Y-----------AGYDEHKKAHDEFIHQLDHWK-----------------------GDSNWAKTWLVNHIKTIDFKYKGKI
---------W------------D--HK-LV-------------------------------L------HF--E--LF-----------------------HK--H--FI-----------------------------------------WLV-HI---D--Y---L
>Golfingia vulgaris Hr2 (Sipuncula) 113aa CAG14946.1  GI:57282928
GFPIPDPYVWDPSFRI-FYSIIDDEHKTLFNGIFHLGHD--------------DSADNLAELRRCTGKHFLNEQVLMQDSQ--Y-----------AGYAEHVRAHDGFIHQLDNWH-----------------------GDVKWAKNWLVNHIKTIDFKYKGKI
>Phascolopsis gouldii Hr (Sipuncula) 114aa P02244  GI:21264441
GFPIPDPYVWDPSFRT-FYSIIDDEHKTLFNGIFHLAID--------------DNADNLGELRRCTGKHFLNEQVLMQASQ--Y-----------QFYDEHKKEHETFIHALDNWK-----------------------GDVKWAKSWLVNHIKTIDFKYKGKI
>Phascolopsis gouldii MHr (Sipuncula) 119aa P27686  GI:232242
PFDIPEPYVWDESFRV-FYDNLDDEHKGLFKGVFNCAAD-------------MSSAGNLKHLIDVTTTHFRNEEAMMDAAK--Y-----------ENVVPHKQMHKDFLAKLGGLK---APL---------------DQGTIDYAKDWLVQHIKTTDFKYKGKL
>Siphonosoma cumanense Hr (Sipuncula) 113aa P22766  GI:123040
GFPVPDPFIWDASFKT-FYDDLDNQHKQLFQAILTQGNV--------------GGATAGDNAYACLVAHFLFEEAAMQVAK--Y-----------GGYGAHKAAHEEFLGKVKGGS---ADA--------------------AYCKDWLTQHIKTIDFKYKGKL
>Sipunculus nudus HrB (Sipuncula) 119aa CAG14943.1  GI:57282922
PFPVPDPFVWDTSFQV-FYFKLDDQHRAIFETLFNSTND--------------NTPGNLQLFYIVTANHFEEEEGWMVSAS--Y-----------GGYDAHKKLHEEFLAKVRSFS---APV---------------SKENLFYAKDWLVQHIKTIDFKYKQLL
>Sipunculus nudus HrA (Sipuncula) 119aa CAF74912.1  GI:57282893
PFPVPDPFVWDTSFQV-FYFELDEKHRGIFQALFALGND--------------NNPSNLKLLYRVTANHFAEEEGFMQTAN--Y-----------GGYRSHKVLHEDFLAKLRSFK---APV---------------PDKDVYFAKDWLVQHIKTIDFKYKNLL
>Sipunculus nudus MHr (Sipuncula) 119aa CAG14944.1  GI:57282924
GFPIPDPYVWDESFKV-FYQLLDDKHKQIFQGVFDCAKD-------------PSSAAKLQKLIEVTAKHFSDEENMMQQSK--Y-----------SGYPPHKKAHEEFLGKLRGLR---APL---------------DTAGLDYCKDWLVQHIKTIDFKYKGKL
>Themiste dyscritum (Sipuncula) 113aa P02246  GI:123041
GFPIPDPYCWDISFRT-FYTIVDDEHKTLFNGILLLSQA--------------DNADHLNELRRCTGKHFLNEQQLMQASQ--Y-----------AGYAEHKKAHDDFIHKLDTWD-----------------------GDVTYAKNWLVNHIKTIDFKYRGKI
>Themiste zostericola Hr (Sipuncula) 113aa P02245  GI:123042
GFPIPDPYGWDPSFRT-FYSIIDDEHKTLFNGIFHLAID--------------DNADNLGELRRCTGKHFLNQEVLMQASQ--Y-----------QFYDEHKKAHEEFIRALDNWK-----------------------GDVKWAKSWLVNHIKTIDFKYKGKI
>Themiste zostericola MHr (Sipuncula) 118aa P02247  GI:417114
GWEIPEPYVWDESFRV-FYEQLDEEHKKFKGIFDCIR-D--------------NSAPNLATLVKVTTNHFTHEEAMMDAAK--Y-----------SEVVPHKKMHKDFLEKIGGLS---APV---------------DAKNVDYCKEWLVNHIKGTDFKYKGKL
---------W------------D--HK-LV-------------------------------L------HF--E--LF-----------------------HK--H--FI-----------------------------------------WLV-HI---D--Y---L


ARCHAEA
>Haloarcula marismortui (Euryarchaeota; Halobacteria) 617aa (9-146) YP_134671.1  GI:55376820
TDSTGEFAEWDDERYSTNIERFDEQHKRLFGLLNDLHTA------MNEGHSEEEVGDILRELERYTEYHFGDEEEFMQNCG--Y------AMDCSDCFYNHREMHEEFADKVQELREKHENGE------------YVTMEVLTVARDWLDAHIAAGDEDQNYAD
---------W------------D--HK-LV-------------------------------L------HF--E--LF-----------------------HK--H--FI-----------------------------------------WLV-HI---D--Y---L
>Methanocaldococcus jannaschii (Euryarchaeota; Methanococci) 169aa (21-153)  NP_247732.1  GI:15668928
EIMKKEIIKWSKDFET-GIKAFDDEHKILVKTLNDIYNL------LNEGKRDEAKELLKRRVVNYAAKHFKHEEEVMEKYG--Y-----------PDLERHRKTHEIFVKTVIEKLLPKIEEG----------SENDFRSALSFLVGWLTMHIAKPDKKYGEW-
>Methanocaldococcus jannaschii (Euryarchaeota; Methanococci) 147aa (1-132) Q58157  GI:21263658
--MKKEIIKWSKDFET-GIKAFDDEHKILVKTLNDIYNL------LNEGKRDEAKELLKRRVVNYAAKHFKHEEEVMEKYG--Y-----------PDLERHRKTHEIFVKTVIEKLLPKIEEG----------SENDFRSALSFLVGWLTMHIAKPDKKYGEWF
Methanoregula boonei (Euryarchaeota; Methanomicrobia) gi|154149997|ref|YP_001403615.1 135aa
----MALISWSDELSVRVNEIDD-QHKKLISLMNNLHDA------MRAKQGKQALEATLQELAAYTVYHFQTEEKYMQKFH—Y-----------PQFATHKAQHDAFVKKVSDFQKEFEANR-----------LGLSIELMNFLRDWVSNHIKSTDKQYSATF
>Methanospirillum hungatei JF-1 (Euryarchaeota; Methanomicrobia) 135aa YP_504166.1  GI:88603988
>Methanospirillum hungatei JF-1 (Euryarchaeota; Methanomicrobia) 135aa YP_502435.1  GI:88602257
----MAFLKWSESLSV-HVTEIDEQHQKLIQLINTLHDA------MLEKKGKEVLGKILDELAAYTVYHFSTEEKYMEQCG--Y-----------PGLAFHKKEHDTFVGKVESLIQDYQANK-----------LGITIELMTFLKDWVSNHIQITDKKYSDTF
---------W------------D--HK-LV-------------------------------L------HF--E--LF-----------------------HK--H--FI-----------------------------------------WLV-HI---D--Y---L

BACTERIA
>Acidobacteria bacterium Ellin345 (Acidobacteria; Acidobacteriales) 132aa YP_592725.1  GI:94970677
----MPFLTWETRFSV--GAAMDAEHKTWFGILNRLHDAM------AQGKGKDVQQKILIEMVAYTHTHFAHEESFLQSRN--Y-----------PDLPAHRSLHVTFTKKVRDLESKVKAGL-----------PVLSVELMDFLRDWLQNHILTHDVKYGTWM
---------W------------D--HK-LV-------------------------------L------HF--E--LF-----------------------HK--H--FI-----------------------------------------WLV-HI---D--Y---L
>Acidobacteria bacterium Ellin345 (Acidobacteria; Acidobacteriales) 134aa YP_593517.1  GI:94971469
-----MTIRWKSSYSV-GVSVMDNEHRSLFNVMHELDNAL------AAGSGQLVIDTVLAKVTNWAEQHFTHEEELMLENG--F-----------PGYEAHRAEHDQARRQLETYEHDRKAG-----------KQGVPVSLLLFLDSWLRQHILKIDLQYAGYL
>Acidobacteria bacterium Ellin345 (Acidobacteria; Acidobacteriales) 142aa YP_591227.1  GI:94969179
---MWAAFAKPEDFPNLGHEAIDDEHENIARLMNELHDA----------------IHHRVALSEQRRLHHELETYVRVNNRS---EEQMMETDSYPNRESHHKAHEAMYRKLYEYEKVLQAGR-----------EDASLQGLHALRELLIRHIAQEDSRVASWH
>Acidobacteria bacterium Ellin345 (Acidobacteria; Acidobacteriales) 152aa YP_592726.1  GI:94970678
----MPDLQWKDSDSV-GIASLDAQHQGLFATAHRLRDA------MRSGRAATIQQEILAELGAYTRSHFEREERVMQICA--F-------PALCGHQHLHRSFLEQLQAIQAELRSDRSGIT---------------PDRMEFMRVWMRKHVAIEDAQYATWI
>Acidobacteria bacterium Ellin345 (Acidobacteria; Acidobacteriales) 155aa YP_592724.1  GI:94970676
----MDEVGVRDRLTF-GIPRLDSQHEQLLQLVRQMREA------DAGGKSLCELRLLAATLTLHTRMHFMDEEMFMEGAG--Y-----------PGLEAHRRRHAEFTEGLLRLQSGLLSTS-----------RSQFARIVEYELGWIHQHLEDEDNELGRWL
>Acidovorax sp. JS42 (Betaproteobacteria; Burkholderiales) 168aa YP_984586.1  GI:121592690
----MAALQWSEELAL-DLPQMDHTHQEFVALLQAVESADDVA-----------LIGAWEDLVAHTDAHFAQEDRWMQATR--F-----------AAGNCHSTQHQVVLKIMREGTDRARQGE-----------LHVLREMAAELAVWFPQHTDAMDASLAQHL
>Acidovorax sp. JS42 (Betaproteobacteria; Burkholderiales) 173aa (28-156) YP_984955.1  GI:121593059
RRPCMPFMPWTAELEL-GLPEIDDQHRQLVALTNALHDELSAA-----SPRRSAVGEIWEGLVDYTHNHFIVEEVLFQQHD--Y-----------AETPAHKAEHDGFTRKAMDLLTRFDGGQ------------EVTLEALEFLKQWLVHHICKVDRAYLPFM
>Acidovorax avenae (Betaproteobacteria; Burkholderiales) 171aa YP_968695.1  GI:120609017
----MAALQWSEGLAL-DLPLMDETHEEFVALLAAVEQAGDE-----------ALLPAWRALVAHTTEHFAREDAWMAATR--F-------ASGNCHSLQHKVVLQVMGEGTARAEAGESAVL---------------RHMAGELAVWFPQHTQTMDAALALHL
>Aeromonas hydrophila (Gammaproteobacteria; Aeromonadales) 678aa (539-672) YP_854665.1  GI:117619147
ATQDQELLHWNEAFMV-QVSSMDNQHLGLFEAMNRLYQA------VLDKSPAQLRKQRLDELLKLATQHFADEERVMEQAG--Y-----------PELRRHKQEHARLLAELDTLMQRNGTDD-----------EEFNMELLVFLKNWLLNHISKVDKQYSGTL
>Aeromonas hydrophila (Gammaproteobacteria; Aeromonadales) 809aa (673-806) YP_857472.1  GI:117618414
LDPEQGSLVWQSSHEC-GHPRIDAEHRQLFVLTNRLLEH-----MKDHQEHPALFLECMESLFDVARHHFAFEEQMLAELG--Y-----------PELAGHQQDHQLLLQKASQLMTAARAGT-----------LGNDELLNFIIQELVVGHMSQADRRYFPLM
---------W------------D--HK-LV-------------------------------L------HF--E--LF-----------------------HK--H--FI-----------------------------------------WLV-HI---D--Y---L
>Alkalilimnicola ehrlichei (Gammaproteobacteria; Chromatiales) 161aa (1-127) YP_741251.1  GI:114319568
----MSYLSWSSDLDT-GIDAIDKQHRRIVEYINQLEDARSQG-------DRALIGEVLEGLVDYTQSHFAFEEQMMEDAG--Y-----------PLLRGHRRVHQLFIKQVGEYCRRFDAGE------------DVAEELLNLLQRWLFNHIRHDDAAYVDDV
>Alkaliphilus metalliredigenes(Firmicutes; Clostridia) 137aa ZP_00800966.1  GI:77685572
------MFKWKESFNC-EVGSVDQQHQKLFQIGMQLHEIVKAK---DQLDHYDEMMEVLQELKHYTIDHFQYEEALMEKHH--Y-----------PGIADHKEAHQAFINKVIEIENQDIDEK----------QKKIGMDIIIFIANWIESHIMKVDMEYKEFF
>Alcanivorax borkumensis SK2 (Gammaproteobacteria; Oceanospirillales) 154aa (1-127)YP_691784.1  GI:110832925
-----MRLVWGPAYEL-GILVIDQQHKRIVDYINDLDRLVGQP------GAQLGVARVLYDLVDYTESHFSFEEALMERAG--Y-----------DKLDDHHSQHRKFIFHIESLLRRHEEGV------------DVAESLLRVLEKWLFHHILEEDRAYTQQV
>Anaeromyxobacter sp. Fw109-5 (Deltaproteobacteria; Myxococcales) 132aa ZP_01667972.1  GI:121536194
-----MTVSFTRSMQI-GVPEIDQQHRELFARLGGFDDALGR-------GDRLEIEATFGFLREYALVHFECEERLMREAD--Y-----------PRLEAHRAIHRTFVERLEALSREHAERG---------PSAFLRLRARNWIVVWLTDHVGGEDVALGRHL
>Anaeromyxobacter sp. Fw109-5 (Deltaproteobacteria; Myxococcales) 133aa ZP_01668151.1  GI:121536373
------MLKWTPALAV-GIEEIDEQHQELFRRAERFVASLETA-------DRQEIGILLSYLRMYCVTHFGAEEAWMRDVR--Y-----------DGYAAHKAEHDRFVKDVLRLSDEHERRG---------GPGLEAMKVGSYLSRWLREHVTSTDTAFARFL
>Anaeromyxobacter sp. Fw109-5 (Deltaproteobacteria; Myxococcales) 135aa ZP_01669396.1  GI:121537620
---MVETHAWNEKLDV-GHEAMDHDHHLQIGLVSAFVDAVER-------GRPWMARRLADQLLAYSVAHFGSEELLMEGSA--F-----------PERDAHAGEHGAFLEQMRELQRSFSSG-----------DEALATTSALELRGALAAHMNGADRRLAAHV
>Anaeromyxobacter sp. Fw109-5 (Deltaproteobacteria; Myxococcales) 142aa ZP_01670274.1  GI:121538500
-----MPYVDSSQLPDLPLAFMNRDHAEEFRLLEELGQALEAH--GQGSGTTDEILQRLAVLAVKTREHFLHEESVMREAA--F-----------PAYLPHKMEHDRVLAEMDAEAKAFRERG----------DTKRLRRYLIDSVPRWFVAHTTSMDVVTAGF
>Anaeromyxobacter sp. Fw109-5 (Deltaproteobacteria; Myxococcales) 149aa ZP_01669455.1  GI:12153767
-----MALQWTSALSS-GVAELDAQLEELFRRVDRLLDAILAR-------DRSEASRLIAFLQHHVSDHFAAEERLMREVE--Y-----------PDAARHVAEHLAFAAQIDALAGWLAAEG---------ATARLVLRLEREVTAWLQEHVYGTDHALARFV
>Anaeromyxobacter sp. Fw109-5 (Deltaproteobacteria; Myxococcales) 195aa (44-177) ZP_01671347.1  GI:121539576
QEARLMWIQWHSGHAV-GIESVDAKQRDLLHRTNRLVETVRA-------GRTDEAVTRLELLRVAAEAQFRDEEARLRD----A---------GSRSLARHAHEHERFLSDLDELVDGVTRRG---------IVALQEVRFTSWLPRWIDIHVARTHSDLASLG
>Anaeromyxobacter dehalogenans (Deltaproteobacteria; Myxococcales) 133aa YP_467301.1  GI:86160516
------MIAWTPALAV-GIEEIDAQHQELFRRAERFVSSLG-------QTSRQEVGILLSYLRLYCVTHFGAEESWMRQVE--Y-----------PGYAQHKAEHDGFVASLMALSDEHEKRG---------GPGLQATRVSTFVERWLQDHVTSTDVEFAKFV
---------W------------D--HK-LV-------------------------------L------HF--E--LF-----------------------HK--H--FI-----------------------------------------WLV-HI---D--Y---L
>Anaeromyxobacter dehalogenans (Deltaproteobacteria; Myxococcales)    133aa YP_466223.1  GI:86159438
-----MVIEWTPSLSV-GVAEIDAQHQELFRRAARLIVALR-------AGDRTEVQPLLEFLADYVIEHFEAEEREMRAAG--F-----------PEYAAHKAAHDAFRRDFEELAGEVQRSG---------ASPLAALTLHNWISDWLRRHIAGVDQELGRYL
>Anaeromyxobacter dehalogenans (Deltaproteobacteria; Myxococcales) 133aa YP_466224.1  GI:86159439
-----MGARWSPALEL-GHDVIDRQHQELFRRYESLVQALA-------RGDRAEVAPLFEFLGSYVVEHFADEERIMSETA--F-----------PGLTVHKASHDRFVREYHALRELFERAG---------PTAAIAVRAETWIADWLATHIGATDAHLARHL
>Anaeromyxobacter dehalogenans (Deltaproteobacteria; Myxococcales) 138aa YP_463433.1  GI:86156648
-----MAITWDPTLAL-GIEEIDAQHEELFRRVDALLESVQE------RRSAEETRRMLAFLDAYVVEHFGAEEALMQAHR--Y-----------PGLAAQRAEHAGFAADLAALREELERDG---------MNALLVVRVNARVATWLFEHISRSDRAFGRFL
>Anaeromyxobacter dehalogenans (Deltaproteobacteria; Myxococcales) 144aa YP_463788.1  GI:86157003
-----MALHWTSVLSI-GVPELDAEHEEMFTRMDRLHDAIL-------SGERAEVSRMIEYLREHAVRHLAAEETLMLAIG--Y-----------PDRERHLREHEAFLATVRELTRRHDANG---------PTAVLVHDVERAVASWIDGHVGIHDVALGAFA
>Anaeromyxobacter dehalogenans (Deltaproteobacteria; Myxococcales) 144aa YP_463705.1  GI:86156920
---MIEAHAWNEKLDL-GHEAMDHEHHLQIALVTALTEAIEQ-------ARPWMARQLSAQLLSYSGVHFGSEEMLMQASA--F-----------DGLADHASEHRTLLEAMREIQAALERG-----------EDDLALAFAVELRAGLAGHMAGSDRRLADHV
>Anaeromyxobacter dehalogenans (Deltaproteobacteria; Myxococcales) 146aa YP_463306.1  GI:86156521
-----MGMEWTARLAV-GIDDIDAQHQELFRRVNGLVTAMGE------RRGREELAEVVAFLRAYVDVHFAAEARLMARSR--Y-----------PGRAFHAAEHAHFRAQVEELAARIAAEG---------PESHHAVKLAGILCEWLRQHVLLTDRQLADHV
>Anaeromyxobacter dehalogenans (Deltaproteobacteria; Myxococcales) 147aa YP_463596.1  GI:86156811
----MRFVDPTQIPELPLPFMND-DHAEEVRLLEALGEALEA--HRARTGGLDVVLERLSLLAVHTREHFLREEQVMRESG--F-----------PAYPQHKGEHDRVLAEMDGEARAFREGG---------DPVRLHAYLFETVPAWFVQHIRTMDAMTARFV
>Aquifex aeolicus (Aquificae; Aquificales) 131aa NP_214180.1  GI:15606800
-----MLIRPNKVQKV-GNLFMDTVHEEEIRLLNQLYDTLMK-------KDVEKADQLMDELLVDLEDHFTTEEELMREFE--F-----------FAYPMHKAEHDTMRKRFKEVYDKWKKEK---------NPEEVVRFLKEEFVPWLKSHVARWDSTTAQQL
>Azoarcus sp. BH72 (Betaproteobacteria; Rhodocyclales) 136aa YP_932039.1  GI:119896826
----MAELVWDDARHT-LGVAEMDATHREFVELVGRLAAADDA----------SFPKLFAEFHDHTRDHFFAEDAKMKATR--F-----------NAIGEHISEHQRVLIELRSFNRNVQAGR----------MRFARAYVRENLKEWFDLHLATMDSALAAHL
---------W------------D--HK-LV-------------------------------L------HF--E--LF-----------------------HK--H--FI-----------------------------------------WLV-HI---D--Y---L
>Azoarcus sp. BH72 (Betaproteobacteria; Rhodocyclales) 150aa YP_935261.1  GI:119900048
MQKNTTLVTWQDNYAL-GLDEIDDQHKMLFDIMNRLWAAILR------NAEGQELGRILEDLERYTVLHFTEEETFMRSIA--Y-----------PDFDGHIAYHRKFVKQISDEKAAARSGR------------QVSLELIHFLRDWLVNHILVEDKRYAAQF
>Azoarcus sp. BH72 (Betaproteobacteria; Rhodocyclales) 160aa YP_932615.1  GI:119897402
---MAELFEWNDDYNV-GVADIDEQHLELVALINQLHDAIRS------HKASKQVRRTLDELAEYTRTHFAIEERLMRESN--Y-----------PDYESHRSYHEALIDQIRALQAKLDGGE-----------ASITFELLHFLRTWLIRHICDVDKQFGAYF
>Azoarcus sp. BH72 (Betaproteobacteria; Rhodocyclales) 162aa (1-135) YP_932470.1  GI:119897257
----MQQMEWGEQLAL-GLGRMDTTHREFVELYNLLAASEAE-----------DFINRLDAFIVHTEAHFDQENRWMEQVN--F-------------PGCHRAEHDRVLTVMRDVRERAARGD-----------AFLGRRLVEELPPWFENHVNGMDAALAFHL
>Azoarcus sp. BH72 (Betaproteobacteria; Rhodocyclales) 174aa (19-147) YP_935260.1  GI:119900047
-LENTVPITWTSDLDT-GIAVIDQQHMRIVDYINQMEHAQQKQDRGL-------VGRVLDELVDYTLSHFAFEESLQEEAG--Y-----------PYLKPHRKVHELFVRRVNEYRERFSRGE------------EIAEEIHTLLSAWLINHIKRDDADYVGAV
>Azoarcus sp. BH72 (Betaproteobacteria; Rhodocyclales) 196aa (1-128) YP_157941.1  GI:56476352
---MNPLMRKLSPSAT-HMIRTDHSHVLSVFHQYETDTSP------------QTKKALVNSACLSLEIHAQLEEEIFYPA---M----RQAMAESATVDKSVSEHEEMRRLITQLRGMEPTDPQYD-------------ETFMSLMRDVLHHAAEEETIMLPKA
>Azotobacter vinelandii (Gammaproteobacteria; Pseudomonadales) 134aa ZP_00417575.1  GI:67155948
-TPQEMLERFQDSYAL-GDETIDHTHREFLELCLAAANAGGP-----------EFAGAFKALFEHTSRHFADEEAKMQASG--Y-----------PAFGEHRANHQRILGDMDRFCQRAVAG----------RAQMARAWLNDSLPAWFDLHAKTMDSALVVYL
>Bdellovibrio bacteriovorus (Deltaproteobacteria; Bdellovibrionales) 136aa NP_970264.1  GI:42524884
--MAESFFKWDKERLTTHVDAMDHEHQKLIQIMNRLYERNEAK------ASKTELTSIVRELASWTVTHFEHEEKFFDTLA--Y-----------SQAAVHKKIHRDLIERLKGHQAEFEK------------TGVLGPAFFQFLKTWLTAHIMGIDTKYGEIA
>Bordetella avium (Betaproteobacteria; Burkholderiales) 148aa CAJ48206.1  GI:115421695
-----VAMEWHTDLEL-GYAPMDDIHEEFVDIVARLQAAPDDR-----------LAELLAELHLHLASHFGQENAWMEETA--F-----------PPRDCHIGEHDAVLKSVKDVRLLLADQ----------GNTRICRELVAALADWFPGHAAHLDSALAHWL
>Burkholderia mallei (Betaproteobacteria; Burkholderiales) 145aa ZP_00444088.1  GI:67645813
QDLSTELAPELKPELKLDDPFVDAVHTEFVQLLDAAARADDAH-----------FLQAYDVWMDHCRQHFEREERWMASTK--F-----------GPQYCHAADHNEVLKVAADVREKLARD----------AQFELGRRLLVEVAEWFDQHVRTMDSMMVSHL
>Burkholderia mallei (Betaproteobacteria; Burkholderiales) 149aa (8-135) BMA0605 YP_102393.1  GI:53725253
ELAPELKPELKPELKL-DDPFVDAVHTEFVQLLDAAARADDAH-----------FLQAYDVWMDHCRQHFEREERWMASTK--F-----------GPQYCHAADHNEVLKVAADVREKLARD----------AQFELGRRLLVEVAEWFDQHVRTMDSMMVSHL
---------W------------D--HK-LV-------------------------------L------HF--E--LF-----------------------HK--H--FI-----------------------------------------WLV-HI---D--Y---L
>Burkholderia pseudomallei (Betaproteobacteria; Burkholderiales) 141aa ZP_01327209.1  GI:100121156
--MQDLSTELAPELKL-DDPFVDAVHTEFVQLLDAAARADDAH-----------FLQAYDVWMDHCRQHFEREERWMASTK--F-----------GPQYCHAANHNEVLKVAADVREKLARD----------AQFELGRRLLVEVAEWFDQHVRTMDSMMVSHL
```

>Burkholderia pseudomallei (Betaproteobacteria; Burkholderiales) 186aa (44-172) YP_334215.1 GI:76810098
LPMQDLSTELAPELKL-DDPFVDAVHTEFVQLLDAAARADDAH-----------FLQAYDVWMDHCRQHFEREERWMASTK--F----------GPQYCHAADHNEVLKVAADVREKLARD----------AQFELGRRLLVEVAEWFDQHVRTMDSMMVSHL
>Burkholderia phymatum (Betaproteobacteria; Burkholderiales) 147aa ZP_01500286.1 GI:118028824
-MRDLEGFAWHDDYAL-GHQTMDETHHEFVSCVHTLLTAEDA-----------DLGDALAAFAGHAHSHFGDEDASMRETA--Y----------RSAGCHIDEHAAVLRSLDEVRAMLAQG-----------RTDVVRSFARALVDWFPEHVRVMDQGLARWL
>Burkholderia phymatum (Betaproteobacteria; Burkholderiales) 190aa (1-123) ZP_01504458.1 GI:118033012
--------MPYVPTIT-TMIRLDHTHVVAAFHRYGSETAWWR------------KRAIVNSVCRALEIHAQLEEEIFYPA---L----ARIAPTDETLKKSRPEHDELREVIAKLRGMGPEN-------------AAYDSLFMELMRDTLHHVADEETRLLPLA
>Burkholderia thailandensis E264 (Betaproteobacteria; Burkholderiales) 141aa YP_442321.1 GI:83719963
--MQDISNELKPELKL-DDPFVDAVHTEFVQLLDAAARADDAH-----------FLQAYDAWIDHCRQHFEREERWMASTK--F----------GPQYCHAADHNEVLKVAAAVREKVAGD----------AQFELGRRLLAEASEWFDHHVRTMDSMMVSHL
>Burkholderia vietnamiensis G4 (Betaproteobacteria; Burkholderiales) 158aa (13-142) ZP_00427022.1 GI:67549150
VRESDNSLVWSDARLL-GFTPMDDVHKEFYEVALRLVTCTDAS-----------ALAAIEEFEKHAVSHFEQEDEWMRTTN--F----------PPRDCHIDEHEAVLKSVRDVKAAVEAG---------QAGCGLVHDIGMHLYNWFPGHADYLDSALAAWM
>Burkholderia xenovorans (Betaproteobacteria; Burkholderiales) 159aa (7-135) YP_556187.1 GI:91780980
PMKEQEIFSWNDGFKL-GYSPMDKTHEEFVEIVNTMLTCSDSK-----------LAQHLDSFVAHAEDHFSQELCWMKETD--F----------PNTACHAEQHDAVMHSVREVREHLRTG----------GDPEAARRLAAALAEWFPGHADYLDAALAQWM
>Burkholderia xenovorans (Betaproteobacteria; Burkholderiales) 165aa (12-137) YP_555832.1 GI:91780625
PVAAEETFGWSDRFLL-GFAAMDETHREFVDRVAALKRASDAE-----------LPARLAEFAQHAVAHFAQEERWMTTTA--F----------PAAQCHADEHAAVLRSVREVEQMLRDG----------AALTLARDLTQALIDWFPAHADYMDAALSHWM
>Burkholderia xenovorans (Betaproteobacteria; Burkholderiales) 166aa (21-147) YP_557518.1 GI:91782312
VPSGDNSFAWSDARLL-GFALMDEVHKEFYAVALNLVTCTDAT-----------AATAIDRFERHAVSHFEQEDQWMRSTN--F----------PPRDCHIDEHAAVLKSVSEVKEAVEQG---------RAGAEMVRDLGMHLFEWFPGHADYLDSALAAWM
>Caldicellulosiruptor saccharolyticus(Firmicutes; Clostridia) 138aa ZP_00885869.1 GI:82500456
----MSQIEWKEQYTL-GDEGFDEDIKKIVERMNKILHAYEQ-----PESQKDVLEELRFLGNNLAISFSHQEYRMKKYL---Y----------PPYFSHKREHDSFLEKYKKYREEAGKE---------GELVAAVMKIRGIIEEWLENHVIVEDKKLVAFL
>Campylobacter coli (Epsilonproteobacteria; Campylobacterales) 199aa (1-127) ZP_00367250.1 GI:57168111
-----MLPKWDSSFSV-HNAKIDDQHKKLFELAAKVEIVSDRS------VSKNEVKELLAEFFNYMKDHFNDEEKYMQLIA--Y----------PAFEEHKKIHKEIIQTMINLIKDIKST------------NDLKEKLYIIAKKWLLEHILYEDMKVEKWR
---------W------------D--HK-LV-------------------------------L------HF--E--LF-----------------------HK--H--FI-------------------------------------WLV-HI---D--Y---L
>Campylobacter coli (Epsilonproteobacteria; Campylobacterales) 203aa (1-127) ZP_00367884.1 GI:57168753
-----MFPKWDDNYSV-HNAKIDEQHKKLFELAGKVEFMFDRP------VYKDDIKALLADFFNYMKDHFNDEEKYMKLIG--Y----------PGLEEHKKIHKEIIQSMIDLIKNIKST------------NDLKEKLYTVSKKWLLEHILYEDMKVEQYR
---------W------------D--HK-LV-------------------------------L------HF--E--LF-----------------------HK--H--FI-------------------------------------WLV-HI---D--Y---L
>Campylobacter coli (Epsilonproteobacteria; Campylobacterales) 229aa (1-130) ZP_00367885.1 GI:57168754
--MDKLIPAWDEKYSI-NDEKIDAQHQKLFELAAKIENAVYKF------VQRDELKEILTELFNYMKDHFSNEEDYMQEIS--Y----------PYLNEHKIMHKNIIHDMSHLIQNIKTT------------NDLKEKLYTIMSDWLLEHILHHDVMIGNWL
---------W------------D--HK-LV-------------------------------L------HF--E--LF-----------------------HK--H--FI-------------------------------------WLV-HI---D--Y---L
>Campylobacter coli (Epsilonproteobacteria; Campylobacterales) 268aa (1-132) ZP_00368169.1 GI:57169041
-----MEIKWSKDFSI-KNIQLDKQHELIFEITEIANDLALKI-QDNDVQHKNDLKQILTKLFQYVKIHFKDEEKFMESID--F----------PLIEEHRKSHQILLKKTKELLEHSND------------IVKMSFELSTLTKDWILDHFANEDLWIANFT
---------W------------D--HK-LV-------------------------------L------HF--E--LF-----------------------HK--H--FI-------------------------------------WLV-HI---D--Y---L
>Campylobacter fetus (Epsilonproteobacteria; Campylobacterales) 183aa (1-127) YP_892819.1 GI:118474426
-----MIPVWDDKYSV-GNNGIDKQHKKLFELAKKSYIYANKN------ISREDIRNIITEFFEYMKEHFRDEEIYMELIE--Y----------PYLEQHSSIHKDIINSMSNLIKTAKN------------VNDLKENLVIIAEKWLLEHILHHDTRIEEWR
---------W------------D--HK-LV-------------------------------L------HF--E--LF-----------------------HK--H--FI-------------------------------------WLV-HI---D--Y---L
>Campylobacter fetus (Epsilonproteobacteria; Campylobacterales) 385aa (1-143) YP_891782.1 GI:118474590
MDYNKSIFAWSKKFET-NLDKIDDQHHHLVNLINNLSKKLSNS-----NLSKNELIGLFTELFGYAKYHFHDEEQLMAENK--L---------NSEFVKDHVFNHKLFIEEVGILFAGLQS----DDEDDQENLDKTAKYILNFLINWLAFHILGQDKKMARQI
>Campylobacter jejuni (Epsilonproteobacteria; Campylobacterales) 131aa ZP_01378037.1 GI:109690967
-------MTYSEKIISMNNDLLDHQHKELFEISKKLSLMNQRH------VGTKELKIVLRELLIMINRHFSDEEAFMREIE--Y----------PYINHHTRIHRKIILEIEEIIISEAK-----------FVNIMTEKLNLVVQDFIFKHTAKEDSKIVKVL
>Campylobacter jejuni (Epsilonproteobacteria; Campylobacterales) 133aa YP_999954.1 GI:121612294
-------MTYNEKIISMNNDLLDHQHKELFEISKKLSLMNQRH------VGTKELKIVLRELLIMINRHFSDEEAFMREIG--Y----------PYINHHTRIHRKIILEIEEIIISEAK-----------FVNIMTEKLNLVVQDFIFKHTAKEDSKIVKYY
>Campylobacter jejuni (Epsilonproteobacteria; Campylobacterales) 144aa YP_999771.1 GI:121613643
-----MEVKWSRDFSI-KNMQLDKQHELIFEITNLANDLALNI-QDNNTQHKNDLKQILVKLFQYIKIHFKDEEKFMESID--F----------PLIEEHKKSHQILVEKTKELLEHSDN------------IVKMSQELSILTKDWILDHFANEDLWIANFT
>Campylobacter jejuni (Epsilonproteobacteria; Campylobacterales) 199aa (1-127) ZP_01068960.1 GI:86150744
-----MLPKWDNSYSV-HNAKIDEQHKKLFELAAKVEVVSDRS------VSKNEVKELLAEFFNYMKDHFNDEEKYMQLIG--Y----------PNLEEHRKIHKEIIQTMINLIKDIKS------------TNDLKEKLYIVAKKWLLEHILYEDMKVEKWR
>Campylobacter jejuni (Epsilonproteobacteria; Campylobacterales) 202aa (1-127) ZP_01069655.1 GI:86151450
-----MFPKWDDSYSV-HNAKIDEQHKKLFELAGKVEFMFDKP------VYKDDVKALLAEFFNYMKDHFNDEEKYMKLIG--Y----------PDLEEHKRIHKEIIQSMIDLIKNIKS------------TNDLKEKLYTVSKKWLLEHILYEDMKVEQYR
>Campylobacter jejuni (Epsilonproteobacteria; Campylobacterales) 239aa (1-132) ZP_01072240.1 GI:86154039
-----MKVKWSRDFSI-KNMQLDKQHELIFEITNLANDLALNI-QDNNTQHKNDLKQILVKLFQYIKIHFKDEEKFMESID--F----------PLIEEHKKSHQILVEKTKELLEHSND------------IVKMSQELSILTKDWILDHFANEDLWIANFT
>Campylobacter lari (Epsilonproteobacteria; Campylobacterales) 187aa (1-127) ZP_00368466.1 GI:57240517
-----MLPKWCDKYSI-HNKEIDEQHKKLFELAANVEMISDKP------IHKGQIKFLLADFFNYMKEHFAEEEKYMEKIG--Y----------PELVNHQKIHKSIIQSMIDLIQNIKS------------TNDLKEKLNIIASKWLLEHILREDMKIEKWH
>Campylobacter upsaliensis (Epsilonproteobacteria; Campylobacterales) 199aa (1-127) ZP_00370672.1 GI:57242735
-----MLPKWDDSFSV-HNAKIDKQHKKLFELAAKVEMVSDKA------VSKNDVKDLLAEFFNYMKDHFNDEEKYMHLIN--Y----------PELEAHRKIHKEIIQTMINLIKDIKS------------TNDLKEKLYIVAKKWLLEHILYEDMKVQKWR
>Campylobacter upsaliensis (Epsilonproteobacteria; Campylobacterales) 233aa (1-136) ZP_00371184.1 GI:57505255
---MNKKIDWTKEFSI-NNYALDKQHELIFDIAIRANELAKEVLEHYDDSLQEELKKTIVKLFDYIKIHFKDEENYMKEID--F----------PLLEEHKASHKFLVERTKEILNYSKD------------PQNFAKELAILTKNWIVKHFCVDDKWIDTYK
>Clostridium sp. OhILAs (Firmicutes; Clostridia; Clostridiales) 131aa ZP_01361119.1 GI:106893996
------MFWWSSELET-GITSIDEQHRTIFNRAGDFLSLDSS-------TDLQEIHRAFVFLMDYCINHFTEEEQIMMDAD--Y----------ESLEGHRAQHNHLVDRLYELGNEIKD---------TGINEELIDQFKFLVIEWLVNHINESDKHLAKFL
---------W------------D--HK-LV-------------------------------L------HF--E--LF-----------------------HK--H--FI-------------------------------------WLV-HI---D--Y---L
>Clostridium sp. OhILAs (Firmicutes; Clostridia; Clostridiales) 137aa ZP_01360922.1 GI:106893798
-----MIFKWKERFET-GIEEIDKQHKRLFEIGTQIYNLASK---DDGTDHYDAIVGLIHELKDYTVYHFRYEEEQLEKAN--Y----------SDIEAHKMEHQRFIDKLNETEAEDID----------VNQKQVLMDMIEFIINWVSGHIVGTDFKYRELL
>Clostridium sp. OhILAs (Firmicutes; Clostridia; Clostridiales) 142aa ZP_01360653.1 GI:106893528
-------MLWKDKYEL-GVSIVDEQHKELFERVQAFMQVLRS--PASWEDKVQRVNETLEFMKIYVVEHFRDEEAYQKRIE--Y----------PGYEAHKKIHGDMVNYVVQFSDEYEK---------SGCNEQLMQQFAGKLLAWLINHVASEDQRIADYA
>Clostridium acetobutylicum (Firmicutes; Clostridia) 129aa NP_346715.1 GI:15893366
------MFVWKDEFEL-GIDKIDNEHRKLFEIANKGYELLKN---EFYVDKYDKIMDIIVELKEYAEFHFSEEEDYLASIG--Y----------KKLFTHKLEHDSFIKKVESFNIKEID----------YDQDKYIQEMLDFVVTWIKEHILEKDREYID--
>Clostridium beijerincki (Firmicutes; Clostridia) 129aa ZP_00910041.1 GI:82747545
------MYEMKEEYKI-GIDQIDEQHKKLFELADQAYTLLKD---EFAIDKYDKIIHIMDELKDYTIFHFKSEEEYMKSIN--Y----------KRLFTQKIEHDKFIKSLEEIDYKNID----------ENQDESLIKLLNFLNDWLTEHILKTDKLIGE--
>Clostridium beijerincki (Firmicutes; Clostridia) 129aa ZP_00911352.1 GI:82748877
------MYEFKEEFKT-GIDFIDEQHKVLFEIADKAYNLLKN---DFTTDKYDNILELVDELRDYTSFHFKAEEEYMNKIN--Y----------KRMFTQKIEHDAFIKRINEVDFRSID----------ENQNESISTLLQLLNDWLIEHIFENDKLIGQ--
>Clostridium beijerincki (Firmicutes; Clostridia) 130aa ZP_00910329.1 GI:82747836
------MVKWKADYEV-GVKLIDEQHEKLFEIADRAYKLLTN---DFILDKYDRITEILGELKEYTIFHFKSEEEYMLSVG--Y----------KKFLSHKVIHEDFIKSIDNLDLHEID----------LNQDESVKKILEFVVDWIDKHILNEDKFIVEN-
>Clostridium beijerincki (Firmicutes; Clostridia) 133aa ZP_00908279.1 GI:82745769
-----MDLIWDKMWSV-GVDKIDRQHKELFNRINLLVCSMKS------AKGKEEMITTLNFLEEYVIRHFNDEEEIQKKNN--Y----------PNYEIQHKEHEIFKNNLKELRKVFET---------TGVSTLFVISEQQKMSYWWRKHVIELDKDIGKFL
>Clostridium beijerincki (Firmicutes; Clostridia) 135aa ZP_00909310.1 GI:82746807
-----MNLNWDNNLAT-GIVNIDNQHKELFDRINKLLIAMKE------GKGKDEVIGTLNFLEDYVIKHFTEEEEIQKKNN--Y----------PKYSIQHKQHEEFKEELKELRRTFET---------TGTSALFVIKVQNRMSTWWKSHIRDLDKDLGKFL
>Clostridium cellulolyticum (Firmicutes; Clostridia) 130aa ZP_01574112.1 GI:118725472
------MIEWRDEFIL-GIEKIDEQHKKLFQIASEIYEATKN---QLITDKYDEIVRLITELKDYTVFHFTYEEEYMHSIG--Y----------RKLLSHKVEHHDFIEKISNTDYSRID----------KDQDQYLMELLDFTVEWIANHIMKTDKAYTIK-
---------W------------D--HK-LV-------------------------------L------HF--E--LF-----------------------HK--H--FI-------------------------------------WLV-HI---D--Y---L
>Clostridium cellulolyticum (Firmicutes; Clostridia) 132aa ZP_01576604.1 GI:118728026
-----MAIKWRDSLAI-GITEIDDQHKKLFEAIDKLFTACVQ------GKGKEEVVNTIKFLEDYTIVHFNDEQEMHKKYN--Y----------PERDAHRKIHDNFLNSFSKLKEQFEQ---------EGAGPLFISTINKQVVDWLIQHIGKADKAFAAYV
>Clostridium cellulolyticum (Firmicutes; Clostridia) 185aa (44-181) ZP_00909308.1 GI:82746805
VLMHFMNSNWDAVLST-GVDNIDRQHQELFRLINNLVSAMN------EEKSKVEIIKALDSLEEHAIRHINEEEAIQKENN--Y----------PGLEIQQMQHEEFKECLRDIRSFLET---------REISTLFIIHMQQKIFKWCRKHMLNADKDLGEFL
>Clostridium phytofermentans (Firmicutes; Clostridia) 129aa ZP_01353421.1 GI:106886073
------MFEMKPEYMT-GITMIDEEHQKLFELADQLYDLLN---NDFIHDKFDYIVEVINGLKEYAKKHFKDEEEYMMSIN--Y----------KRLFSHKIEHQAFLEKMEGLDLESID----------EDQKQTCLELLGFVSDWLVNHILGNDILIGK--
>Clostridium phytofermentans (Firmicutes; Clostridia) 143aa ZP_01354048.1 GI:106886715
--MFDFAFDWIKELET-GDELIDQQHKEFFRIGRDIEQLLV---RKCIGVQEQELLDIVCELREYVSYHFYTEETIMRQKG--Y----------SNYELHVKEHLGFRKYVMEIDCPALA----------KNPYKELKAIKTMIQSWVFNHMMSEDVRMINEI
>Clostridium tetani (Firmicutes; Clostridia) 137aa NP_782896.1 GI:28211952
------MFNWKEDFSC-NIASIDAQHKKLFELANELYNLLS---LKDEYDRYDDILNTLSELQKYTVYHFGYEEDLMKSVN--Y----------PNFECHKKQHDNFVKKVTNIDKAYID----------ENQKEVLRDLLLFIVDWIEGHILKIDMQYKELF
>Clostridium thermocellum (Firmicutes; Clostridia) 129aa ZP_00504934.1 GI:67875718
------MYEMKPEYFT-GIDFIDKEHARLFAIADEAYQLLK---NEFIPDKYDHIMNLVNELKDYTQYHFKHEEEYMESIG--Y----------KRLLSQKVAHEDFIQKINEYTPDVID----------ENQKETLLELLDFLATWLVEHIYKQDKLIAE--
>Colwellia psychrerythraea (Gammaproteobacteria; Alteromonadales) 131aa YP_268476.1 GI:71281331
----MTQVIWQDSYSI-GIDTIDTQHKQLFDIMNKIYVAS------EEDSDIDVIMPLFDELQDYTKYHFDEEEQYFTT----L---------SQGGIEQHKSEHQFFIDELDKIKQQSLR------------IGTISLELLYFLNDWLVNHIQVEDPKYLDHD
>Colwellia psychrerythraea (Gammaproteobacteria; Alteromonadales) 192aa (59-182) YP_270886.1 GI:71279092
KQKHIKQIKWSKEYSIG-IEYIDQDHKKLLHLLNQFSIAFD------YAQCEEFEREALEELVSYTKYHFKREEKLMEDYG--Y----------PGLAEHQEEHQAMIDKVEEYVAIYNI-----------EGHDSLKQVTNLLTYWLINHIQESDTQYRDYL
>Colwellia psychrerythraea (Gammaproteobacteria; Alteromonadales) 188aa (50-182) YP_270871.1 GI:71282489
KKSNPKQVQWREEYSV-GIAHIDDDHKKLISLLNNFSIAYD------YAMSESFEKEALNELISYTKYHFEREEKMMEDND--Y----------PDLIAHKAQHKVMIEQVEKFVQLYND-----------KGHDALEEIASFLTDWLINHINGTDKQYSAHL
>Colwellia psychrerythraea 34H (Gammaproteobacteria; Alteromonadales) 368aa (1-132) YP_268475.1 GI:71280761
----MKTFIWNSNYET-GVAKVDDQHQCLVEIINEYSHLLA-----NNTTTETDIELTLNKLIDYTKFHFKDEEQLMYSVG--L---------DTRHITHHEKLHRDILDEISTLLSDQVT-----------DKLSIARYILDLIIHWLVYHILGTDMNMAKQM
---------W------------D--HK-LV-------------------------------L------HF--E--LF-----------------------HK--H--FI-------------------------------------WLV-HI---D--Y---L
>Colwellia psychrerythraea (Gammaproteobacteria; Alteromonadales) 519aa (382-510) YP_271339.1 GI:71280063
KVADKVGIQWIAEYSI-GNIEIDEQHKVLFTMINDFFHQDN----------KEAAIMLFQSLSSYIDLHFESEENLLRQIN--Y----------PDTEEHIKKHGELREKFHLLRGKLDD-----------YDLDVHHKIAMFLYNWLAKHILKADMEYKSYA
>Colwellia psychrerythraea 34H (Gammaproteobacteria; Alteromonadales) 722aa (1-123) YP_270299.1 GI:71279208
-------------MSVGIDSLDNDHKKLIAILAQLMSAKN------EKLGQDDIDVIFERLEGYCLSHFAREEALLAKID--Y----------KQLEAHKLSHQAFIKKIPQLKEQWFA--------KVTESEAVKDKIILFLQQWLIKHILEEDLDYVPAI
>Colwellia psychrerythraea (Gammaproteobacteria; Alteromonadales) 1328aa (1175-1306) YP_268669.1 GI:71280946
KSSPIKRIIWTNEMSIDDGGELDEDHKFMFSLLNRCQELLE------EQDDHQSAQAIITEIVQYTQDHFVNEELLMNNIN--Y----------PYAKNHCDVHRMIAKQLSKKVLVCSD-------------KEILRWLITDMSTWLVEHIMEMDKPLHKYI
>Comamonas testosteroni (Betaproteobacteria; Burkholderiales) 165aa (1-124) ZP_01520527.1 GI:118051979
----MASLEWNAALIL-DFPVMDAVHEEFVALLAQVEAAAD-----------EQLCALWDELIAHTQQHFDQEDRWMQTTG--F----------ATGNCHSQQHKVVLAVLREGAVKGEQ-----------GDLATIRHMAAELGPWFSHHAQNMDAALALHM
>Comamonas testosteroni KF-1 (Betaproteobacteria; Burkholderiales) 179aa (37-127) ZP_01521540.1 GI:118052994
PKSTEQPPLWSEALLL-GQVPIDDEHERLAWLIERMQRASA-----------SDMAQALEAVIAHAASHFAAENELMVGTQ--F----------PPRDCHIREHDAVLSTLRGVLRRLDC----------SDEISVAYRLADELAAWFPAHVQHLDSALSHWL
>Comamonas testosteroni (Betaproteobacteria; Burkholderiales) 211aa (37-167) ZP_01518696.1 GI:118050146
LENIMALLVWVPELDT-GIAEIDRQHRRIVDYINRLYELRS-------SPDREGLGDVIGEMIDYTVSHFVFEESLIESAG--Y----------MFAGPHKKVHELFTRRVIEMQTRFDA------------GEDVAAELHGMLSRWLFNHIRNEDHGYVDSA
>Dechloromonas aromatica(Betaproteobacteria; Rhodocyclales) 125aa YP_284639.1 GI:71907052
-------MVWDETRHALGVPEMDGTHREFVELAYALLVASD-----------EEFPALFVRLHEHTRQHFEHEGKLMKACR--F----------PAIGEHNSEHLRVLGELAHFSRGVAA-----------GRLGMARNYVRALPEWFATHLATMDSALAGCL
>Dechloromonas aromatica (Betaproteobacteria; Rhodocyclales) 127aa YP_285883.1 GI:71908296
----------MNKNLLLGVPNIDHQHRELFRSFQHLLSINS---------GDESFSEALSRLTIQIHQHFKTEEHYMAGLH--M---------PAAELAEHVLAHTQIIEDLTEIHFETMH-----------GLGVPFEEIIKRVASYVNHHVVEFDLQLKPYI
>Dechloromonas aromatica (Betaproteobacteria; Rhodocyclales) 128aa YP_284878.1 GI:71907291
---------MTISILKTGHAEIDRQHAELLICLAGVKEFVG------GQYAFSAGFTAIQALIGYTEDHFAFEEGLLRQWD--Y----------PDLDAHVEQHKSLVAEVRRQWGRVEQ-----------EGADIAREVVETIEGWITDHINVEDRKFSGVV
>Dechloromonas aromatica (Betaproteobacteria; Rhodocyclales) 133aa YP_284887.1 GI:71907300
---MTHSPTWSDDLSV-GNYLIDGQHKRLVSLCARAADCAS----ATSPEALREFHNILNDLAKLTDEDFKDEEVLLIKNG--C----------PNVEAHIAEHNVYREMVVNLLFDGTA------------GLLDRAKLYHVANDYLTKHIIEMDLADKAYL
---------W------------D--HK-LV-------------------------------L------HF--E--LF-----------------------HK--H--FI-------------------------------------WLV-HI---D--Y---L
>Dechloromonas aromatica (Betaproteobacteria; Rhodocyclales) 134aa YP_284625.1 GI:71907038
---MTQPREWSGALSV-CNRWIDEQHRQLIDLCNKAEACAA----LSSHEAHREFHLLLNDLATLVGNHFRDEETLLA-----I--------HESPNLDAHKAAHVSFLERLTQLLYDGAR------------GLIDSRTLHRLIADYLIEHMLEMDMQEKHYL
>Dechloromonas aromatica (Betaproteobacteria; Rhodocyclales) 142aa YP_286895.1 GI:71909308
-----MRIRWSSDLET-GIRAIDLQHEELIGMLNELDAAYS------GGCEQFVLDSVLQRLGAYAEFHFGTEESLMAT----L-------PRSEQHAEEHLRQHRGFIEQLSRMRAASSQ-----------DAPQTMGILIDFLNEWLYEHILKSDRNLGALL
>Dechloromonas aromatica (Betaproteobacteria; Rhodocyclales) 146aa YP_285962.1 GI:71908375
-MDEQQGFAWDDRYLL-GHSAMDDTHREFVALVDALLTVEA-----------DGLEAALADFARHAEAHFDQENRWMELDG--F----------PARDCHVDEHNKVLASVREVQLELAE-----------GNVQLVRELAVALMDWFPAHADYMDSALSTWL
>Dechloromonas aromatica (Betaproteobacteria; Rhodocyclales) 160aa (1-126) YP_284835.1 GI:71907248
-----MPITWDSRLDT-GIDVIDAQHKRIVDYINDLEIAKI-------KGDKVLTMEVIEQLIDYTQSHFGFEESMLEEAG--Y----------KFLKPHKKVHELFIKRVTEFTMRAAK------------GEDIADELHSMLAKWLLNHIANEDRDYSDTV
>Dechloromonas aromatica (Betaproteobacteria; Rhodocyclales) 161aa (10-140) YP_284552.1 GI:71906965
GEHQKSKIQWSDNLLV-GHSGIDETHREFVTLANALSESCE-----------ESAPTRLHQLEEHLMSHFDNEKLLMEKTE--F----------PATDCHIEEHAKVFDAVVLVRERYSD---------GTASLNDIKRLAQALIDWFPGHIFYMDSALSTWV
>Dechloromonas aromatica (Betaproteobacteria; Rhodocyclales) 667aa (529-661) YP_287152.1 GI:71909565
SEKKNQSDWIVFESGHLVGVKIDEQHQELVRMVNKINRAIH------SRCEDASLKLMFNELVAFTEHHFSTELRFMIDFH--Y----------PETDVHDQQHQALVAQLSNLITRFEQ--------------GDELQSLQMIKDWLLRHIEHADKPLGAYL
>Dechloromonas aromatica (Betaproteobacteria; Rhodocyclales) 681aa (1-129) YP_284191.1 GI:71906604
----MDAFVWNERYMT-GEAQVDEEHQELVRIINWVIEHQS------VPAQDDDIQRVLASLANYAVMHFGHEEELMEQMH--C---------DPRFIQAHVAVHQEFARQVGKMLEFQGG-------------KADVEYLLRFLSSWLAHHILGIDQSMARQI
>Delftia acidovorans (Betaproteobacteria; Burkholderiales) 166aa (1-124) ZP_01580041.1 GI:118731526
----MAVLEWSGALAL-ELPAMDEVHQEFVLLLAEVEAADD-----------AQLCARWDELIAHTQVHFDQEDRWMQSTR--F----------TSTNCHSLQHKVVMQVMREGAAKAVAG------------DLAVIRSMAGELAAWFVHHAQTMDAALALHL
>Delftia acidovorans (Betaproteobacteria; Burkholderiales) 213aa (40-170) ZP_01578319.1 GI:118729802
LENVMALLVWVPELDT-GIAEIDRQHRRIVDYINKLYELRS-------SPDREALGDVIGEMIDYTVSHFVFEESLIESAG--Y----------MFAGPHKKVHELFTRRVIEMQTRFDA------------GEDVAAELHGMLSRWLFNHIRNEDTGYVDSA
>delta proteobacterium MLMS-1 (Deltaproteobacteria) 135aa ZP_01288141.1 GI:94264349

```
----MALFTWKEEYSV-NIKEIDDQHKQLVAMVNELHEAMM------QQRAKEVLSSILNKLVTYCGTHFALEERLMQTHG--Y-----------PEYASHKAKHDKMTAKVVSLQNDLKS-----------GKANLTIEVSQFLKDWLDKHILGTDKKYSAHL
---------W------------D--HK-LV------------------------------L------HF--E--LF----------------------HK--H--FI---------------------------------------WLV-HI---D--Y---L
>delta proteobacterium MLMS-1 (Deltaproteobacteria) 148aa(10-142) ZP_01288112.1  GI:94264319
LNTKMAIYSWQDEYSI-GIKEIDEQHKELVKMIDELYTAMV------KRQDQQVLGDILARLVDYCNVHFAAEEALMQSHG--Y-----------PYYRQHKEIHEKMSARVQALQKCFKA-----------GKTDLTIPTSLFIKEWLDKHILGTDQKFATFL
>delta proteobacterium MLMS-1 (Deltaproteobacteria) 296aa (150-284) ZP_01291413.1  GI:94269245
GGAIEPAVVWNEEFEV-GVEVIDRQHRELISRINRMLAAIR------QGKGHDETISLVRFLQDYVVEHFQSEEIYMRRHH--Y-----------PDYDAHKEIHDTFVAELAGLVEELEK---------HGIQPEQVVQIHRRVINWVVDHIFLTDKALRHIK
>delta proteobacterium MLMS-1 (Deltaproteobacteria) 457aa (310-434) ZP_01290755.1  GI:94267120
GGAIEPAVVWNEEFEV-GVEVIDRQHRELISRINRMLAAIR------QGKGHGETLSLVRFLQDYVVEHFQSEEIYMRRHH--Y-----------PDYDAHKEIHDTFVAELAGLVEELEK---------HGIQPEQVVQIHRRVINWVVDHIFLTDKALRHIK
>Desulfitobacterium hafniense (Firmicutes; Clostridia) 135aa ZP_01371607.1  GI:109647704
------MLKWKDEYSI-GVEEIDEQHKHLFEIGNEIYDLLE---NYLLPDKYDKIIQIIAELKEYTVHHFQTEEELMLKIK--Y-----------PGYFKQKVAHDDFIKEVESVDFSAID----------QDQDGHTRKLLEFIFGWVLDHILKSDLGIKSFM
>Desulfitobacterium hafniense (Firmicutes; Clostridia) 139aa YP_517407.1  GI:89893920
-------MIWKEKYKV-GDPLIDSQHEELFSRVGSFVETLR--SDKPWEQKVEKVNETLEFMKDYVVTHFADEEAYQLEG---Y-----------PHFEEHQMIHKNMVAYVVAVSREYAK---------EGFKEELMQQFGGKLLAWLINHVVADDQKIAEYA
>Desulfitobacterium hafniense (Firmicutes; Clostridia) 144aa YP_516471.1  GI:89892984
---MIVMLKWKDEYSI-GVEEIDEQHKHLFEIGNEIYDLLE---NYLLPDKYDKIIQIIAELKEYTVHHFQTEEELMLKIK--Y-----------PGYFKQKVAHDDFIKEVESVDFSAID----------QDQDGHTRKLLEFIFGWVLDHILKSDAILALRV
>Desulfotalea psychrophila (Deltaproteobacteria; Desulfobacterales) 156aa YP_065523.1  GI:51245639
----MAVIKWQDTYNT-GVEHFDGEHQQIVELIDLMFFTLR------DRSGKDVTEEACKEIISYTEQHFIREEQAMRAVA--Y-----------PYLEEHIAEHSRLKAEMLKLQRGISN-----------NFPKGAAKFYRFIHKWFIIHIQEMDKKYAPYC
>Desulfotalea psychrophila (Deltaproteobacteria; Desulfobacterales) 370aa (1-140) YP_064708.1  GI:51244824
----MKTFIWDENFTT-GISDIDKQHHHIIDIINEFAFAFS-----QNSINKDFILRVSKELTTYGVDHFKTEEEIMLAAK--L---------DPRYTSKHIAAHNDFSQEIEAITANLGL-----------TDKENFRQILNFLIHWLSYHILNDDQSMARQI
>Desulfotalea psychrophila LSv54 (Deltaproteobacteria; Desulfobacterales) 374aa (1-134) YP_065775.1  GI:51245891
--MFMKTFIWDKTFTT-GISKIDEQHHHIVEVINKFVFAFS-----ENTIDKDFILHVSKELMDYAQNHFKTEEKIMLAEK--L---------DPLFISQHIDTHNAFSQQIKIFTTNPDF-----------TNHNFFRQILAFLIHWLDDHILNIDQSMARQI
---------W------------D--HK-LV------------------------------L------HF--E--LF----------------------HK--H--FI---------------------------------------WLV-HI---D--Y---L
>Desulfovibrio desulfuricans (Deltaproteobacteria; Desulfovibrionales) 150aa (9-142) YP_386749.1  GI:78355300
AEKHMRQLELKDSMKI-GIKELDDQHQQLTEIINELYYAYMQ------GNHRAVLCGLITRLNDYAHEHFALERQYMERFV--F---------EMPDYEQHMQEHREFFTDAIGFLLRYIE-----------EGSEITPEMLDYLQGWWKDHVMVRDKELGRFL
>Desulfovibrio desulfuricans (Deltaproteobacteria; Desulfovibrionales) 579aa (441-573) YP_386930.1  GI:78355481
STGDGQFLNWTDDLSV-NIGHLDSQHKNLVALINELAAAMK------AGKAKSVMLDTLGRLKEYTLTHFKEEEDLFDRYD--V-----------PGARDHKKIHQSLREKVLSVEQDIVS-----------GRITVSLELLEFLKEWILNHIQVVDKQYSAFL
>Desulfovibrio desulfuricans (Deltaproteobacteria; Desulfovibrionales) 722aa (584-716) YP_389304.1  GI:78357855
TADTGQLFRWTDDLSV-KISSIDEQHKGLVNLINELHEAMR------RRKSASHLIAIVGRLKDYTVYHFDTEEKLFHKHG--Y-----------PATAEHIQEHRKFVENVLAFEKDLQQ-----------GKVTVTMDVMRFLKNWLTGHIKGTDKKYSGYL
>Desulfovibrio vulgaris (Deltaproteobacteria; Desulfovibrionales) 136aa AAF23777.1  GI:6685084
-GDADVLVKWSEDLA--NLPSIDTQHKRLVDYINDLYRAAR-------RRDMDKAREVFDALKNYAVEHFGYEERLFADYA--Y-----------PEATRHKEIHRRFVETVLKWEKQLAA-----------GDPEVVMTTLRGLVDWLVNHIMKEDKKYEAYL
>Desulfovibrio vulgaris (Deltaproteobacteria; Desulfovibrionales) 137aa YP_389304.1  GI:78357855
----MATFTWDASLDV-GIPEIDAQHREMTDIIATLHEAYR------AGRERDVLAELINRINEYAQMHFATERRYMEPVAD-A----------IPNYEKHMQEHREFFSDAIGFLLRYLD-----------NGATITPELLDYLAEWWFRHIRGTDQVMGRVL
>Desulfovibrio vulgaris (Deltaproteobacteria; Desulfovibrionales) 655aa (513-635) YP_012317.1  GI:46581509
ALSGVQRLVWSEDYAC-GEPRIDASHRALLAKADSIMSSLG------GADGLNARRACILALVDAVRMHFREEERVLAAIG--Y-----------PKAGEHAVIHEALLQRGASLVAAWER-----------GGMSLDDVVITLVHGMVMRHMLDDDSAFFEHV
>Desulfovibrio vulgaris (Deltaproteobacteria; Desulfovibrionales) 689aa (550-682) YP_009395.1  GI:46578587
AGGGDKLFEWDDALNI-GVADVDPQHKVLVDLINEVYAAMK------AGADRSVLQDIVRRLREYTVKHFTYEEGVLHTSMR-Y-----------PDMQAHLKQHRAFVERIAQFEEALGS-----------GRVTLDMEMMRFLKNWLKQHIMGTDKKYVPYF
>Desulfovibrio vulgaris (Deltaproteobacteria; Desulfovibrionales) 959aa (823-953) AAB50497.1  GI:887858
TGDADVLVKWSEDLAN--LPSIDTQHKRLVDYINDLYRAAR-------RRDMDKGTEVFDALKNYAVEHFGYEERLFADYA--Y-----------PEATRHKEIHRRFVETVLKWEKQLAA-----------GDPEVVMTTLRGLVDWLVNHIMKEDKKYEAYL
>Desulfovibrio vulgaris (Deltaproteobacteria; Desulfovibrionales) 963aa (827-957) YP_012365.1  GI:46581557
TGDADVLVKWSEDLAN--LPSIDTQHKRLVDYINDLYRAAR-------RRDMDKAREVFDALKNYAVEHFGYEERLFADYA--Y-----------PEATRHKEIHRRFVETVLKWEKQLAA-----------GDPEVVMTTLRGLVDWLVNHIMKEDKKYEAYL
>Desulfuromonas acetoxidans (Deltaproteobacteria; Desulfuromonadales) 132aa ZP_01311722.1  GI:95928977
----MTFVTWNDDFLL-HVDQFDEHHKHLVDLLNETYEKFV------NKTVDLSLEKLFDELIDYATYHFSAEEYWMQAKQ--Y-----------PGYEEHKKEHAYFTKRVVEMHRDFVA-----------GNTSLGLEVLTFIKNWVANHILNSDAEYGRFI
---------W------------D--HK-LV------------------------------L------HF--E--LF----------------------HK--H--FI---------------------------------------WLV-HI---D--Y---L
>Desulfuromonas acetoxidans (Deltaproteobacteria; Desulfuromonadales) 133aa ZP_01311940.1  GI:95929196
----MAFIEWNQNFAV-NVDQFDDHHRHLIDLLNKTHDQIT------RRLEKEKLGLVLVELIEYAKYHFRAEEVWMKNLD--Y-----------PKLKQHCAEHHYFINRVQEMCEDYNS-----------GNTALAQELLSFMKNWLSSHILGTDREYSRFI
>Desulfuromonas acetoxidans (Deltaproteobacteria; Desulfuromonadales) 142aa ZP_01313281.1  GI:95930546
----MATITWKSCYET-GNPTFDAEHRALVNALNDLYEAIR------KKQGDEVLASLLSTLNLYIRKHFQHEEQAMLKHH--Y-----------PGMEAHIKAHRAFKKTVNDYQEQVLT-----------GYTGLASDLYKYLRDWLLNHIVKTDGQFGEFL
>Desulfuromonas acetoxidans (Deltaproteobacteria; Desulfuromonadales) 199aa (35-159) ZP_01313282.1  GI:95930547
KGRRVYVVEWKDAYSN-GIDEVDDQHKHLFFLVKCLRLETI--------------EKTLDDLADYVFSHFSTEQGLMDESE--Y-----------PDRDRHRQIHDQFVSTVAECIADDAP-----------WDDERLHDLRKFLNKWLIGHIMTDDQQFGRWY
>Desulfuromonas acetoxidans (Deltaproteobacteria; Desulfuromonadales) 368aa (1-131) ZP_01313679.1  GI:95930950
----MDVIVWNKNFET-GLAEVDKQHRHLVEITNRFGQILS-----DKNALCKEMEEIFTELVSYTQYHFSEEEHLMESTG--I-------------DSRHIAYHKNEHASFLQSVLHLYG--------ESKLGRDTGKPLFEFLMNWLVYHILGSDRTFGEQV
>Desulfuromonas acetoxidans (Deltaproteobacteria; Desulfuromonadales) 371aa (7-134) ZP_01312822.1  GI:95930083
-MIYMDVIVWNENFET-GLDDVDQQHRHLVEITNRFGRILF-----DKNALCKEMEEIFAELVSYTQYHFSEEENLMETIG--I-------------DERHVDYHKNEHASFLQSVLHLYG--------ESKLGRDTGKPLFEFLMNWLVYHILGSDRSFGEQC
>Desulfuromonas acetoxidans (Deltaproteobacteria; Desulfuromonadales) 591aa (2-127)ZP_01313525.1  GI:95930793
HLDKIEIFPWDKNFDT-GILEIDRQHQRLVELLNLLVSHLA------FQSQAPTLNTIFDELKSYALIHFKTEEEIWHKA---L--------DDDPWVIDHQETHAQFVVEVKRLKGEKSD----------RCFDDVVADIVAFLTNWLALHIIDTDKRLAKAV
>Desulfuromonas acetoxidans (Deltaproteobacteria; Desulfuromonadales) 1174aa (1032-1164) ZP_01313825.1  GI:95931099
SINNVLQLSWSDKFCC-GHETIDEQHMGLFALGNELIEAFL------MRQDKRLVDDKLEELYKNTCDHFSFEEQLLEEVH--Y-----------PNVVEHRQEHRHMEEKLVSLIEIYQQ-----------NRADYSELLHYISIDLISGHLLCTDNDYFAYL
>Endoriftia persephone (Gammaproteobacteria) ref|NZ_AASF01001681.1
--------VWKESYSV-GIAQIDDEHRRLIELLNKFQAA------YKYHMGEEFEQQALKALVDYTKYHFKNEESLMQKYD--Y-----------PDFNGHVEQHRAMIAEVDSFVRQYEEK-----------GHEALEGVVLYLQGWLIKHINGTDKQYSSFL
>Geobacter sp. FRC-32 (Deltaproteobacteria; Desulfuromonadales) 132aa ZP_01390530.1  GI:110602439
----MQLVKWDNTLTL-GVEPFDDHHKHLVCLLNETYDLFI------AGEKPSSLEEVIDELIDYATYHFSSEELWMVKLN--Y-----------PKQEEHKKQHEEFSSRVVNFQRDLIQ-----------KGSTSTLEVLSFLQRWLVEHIKHSDGEYSRFI
>Geobacter sp. FRC-32 (Deltaproteobacteria; Desulfuromonadales) 135aa ZP_01387533.1  GI:110599293
----MSLITWNQSLSV-NVKQFDEQHKKLIDLINKLHDAMK------AGKGKDVLGEILQSLVDYTKQHFAAEEALLQKHG--Y-----------GDYEKHKKEHNLLVMQVADIQKKLKE-----------GSPVLTQTVMTFLKDWLSNHIQREDQKYGPFL
>Geobacter lovleyi (Deltaproteobacteria; Desulfuromonadales) 135aa ZP_01593656.1  GI:118745688
----MALLLWGPMLEV-GVKEIDTQHRKLVDLANELSDAQM------AGKAKDVLGKTLSELVRYTVTHFSTEERLMDQHK--Y------PAAQKGVKVDHKQQHKDLVKTVSDFKAKFDK-----------GDAALSQEIMHFLRDWLTKHIMNTDKAFARDL
---------W------------D--HK-LV------------------------------L------HF--E--LF----------------------HK--H--FI---------------------------------------WLV-HI---D--Y---L
>Geobacter lovleyi (Deltaproteobacteria; Desulfuromonadales) 137aa ZP_01594022.1  GI:118746066
-----MGITWREDLAI-GVDQIDDQHKELLARFDLLLSSCK------EGKGREEVLHLLTFLDDYVISHFSDEEKLQKQSG--F-----------PEYESHHAEHVSFIARLAELKERMRS--------EGGVQIDHVLDTNKLLLDWLLRHISVRDRALGRHL
>Geobacter lovleyi (Deltaproteobacteria; Desulfuromonadales) 147aa (1-130) ZP_01593713.1  GI:118745747
MSSYSPLNTFGYVATQ-HLMPYDSRQARLISVLDAIIATAD-------QVPSAELEQQIEDLLGNVLGQTASENVHMFTID--Y-----------LAYEDHKLDHFRICTTIAGIRCKAAR-------------ALCIDDDLQTLRKLLSLHIDKHDQMLEQYL
---------W------------D--HK-LV------------------------------L------HF--E--LF----------------------HK--H--FI---------------------------------------WLV-HI---D--Y---L
>Geobacter lovleyi (Deltaproteobacteria; Desulfuromonadales) 147aa ZP_01592161.1  GI:118744164
-----MGIYWRKELEI-GVIEIDKQHQSLVESFNAFLLACR------NGKGSSELLELLKFLDEYALHHFHTEEAIQKAAA--Y-----------PEYESHRLQHLGFTKKIKDLKEQLTL--------NEPPQINHLIATNTVLLDWFIKHISGSDSHFGRHL
>Geobacter lovleyi (Deltaproteobacteria; Desulfuromonadales) 518aa (379-512) ZP_01593682.1  GI:118745715
HFKLANALVWDDSFAT-GVHRYDEQHKVLFRMVNDLHDAMQ------QKRTKEAIGEILRGLADYTVNHFADEEKVFAQTR--Y-----------PDEANHKLLHKALVDQVVALIGKFQS-----------GETVLSQDVITFLQKWLIDHIKGQDKKYGPHL
>Geobacter lovleyi (Deltaproteobacteria; Desulfuromonadales) 768aa (620-753) ZP_01594207.1  GI:118746259
ASRSIIRLAWKAEYLS-GEPGIDAEHQQLFRMANQLFEQAA-----SQKSDPAGFNAALDDLLQHVTSHFDNEEALLRRIG--Y-----------SELEEHVRQHSQLKEQAVSLRQVADK-----------REISTGELVDFLAIRVVHEHLLEADRNYFPLL
>Geobacter metallireducens (Deltaproteobacteria; Desulfuromonadales) 135aa YP_386060.1  GI:78224313
----MALITWNDSLSV-KVKQFDDQHKKLVEMLNQLFDAMK------AGKGSLVVGDILKQLIAYTQTHFAAEERLMTQYG--Y-----------ADLEIHKKEHNALVVQVLDLQKQFQD-----------GKAVLTQNVMTFLRDWLSKHIQGDDKKYGVFF
>Geobacter metallireducens (Deltaproteobacteria; Desulfuromonadales) 155aa (12-146) YP_383801.1  GI:78222054
TFQFRESCMWTDDLAV-GIDEIDSQHKALFEQLDKLLDACV------AGGEREEVVTMLCFLDQYVVAHFSAEEKLQQEYG--Y-----------PGYEKHRAEHEEFMRRINRFVEEVAA---------AAPTRDLVLRVNQTLIDWLKFHILAVDKEMSEFL
>Geobacter sulfurreducens (Deltaproteobacteria; Desulfuromonadales) 131aa NP_953971.1  GI:39998020
-----MAIGWRDDLLT-GVVEVDDQHKELFRRFGELLTACN------QGKGGEEVLRLFNFLDDYVVKHFAAEERIMRQHG--Y-----------PDYLEHKQQHQGFTRRLEELKRQFRD---------EGAGLSLVITTNHVMIEWLSRHIEKMDKEIAKYV
>Geobacter sulfurreducens (Deltaproteobacteria; Desulfuromonadales) 134aa NP_952095.1  GI:39996144
------MVVWKDEYLI-GVEQVDRQHRELFARFHRLMDALT------GGTARHELLETIAFLESYAENHFREEESLQQECH--Y-----------PDAGFHRMAHETFRRQLGDITASLAE---------QGVTNLLVIETGRTLFQWLVDHVCGMDREFASYL
>Geobacter sulfurreducens (Deltaproteobacteria; Desulfuromonadales) 135aa NP_951461.1  GI:39995510
----MALISWSDSLSV-KVKQFDDQHKKLVDMVNQLFDAMKA------GKGNQVMGDILKSLIQYTQTHFAAEERIMKQYG--Y-----------PDFEAHKKEHNALVMQVLDLQKQFQE-----------GKAVLTQNVMTFLRDWLSKHIQGDDKKYGVYL
>Geobacter sulfurreducens (Deltaproteobacteria; Desulfuromonadales) 139aa NP_953680.1  GI:39997729
--------MWTDDLTV-GIWEIDTQHRALFAQLEKLLDACMV------GRELEEVMEMIVFLDGYVDTHFEAEERLQREGG--Y-----------PEYEKHRAEHLIFRTNLERLKAELVE---------SGATRDLVLRVNQVLIDWLKTHIRNVDAAMSEFL
>Geobacter sulfurreducens (Deltaproteobacteria; Desulfuromonadales) 145aa (11-142) NP_951317.1  GI:39995366
NKASQKFIVWQDSYSV-GVPKLDGQHKKIIDLINEVYDYLQG------RTGLLDLGRILDDLSTYTQNHFADEEQLLRLAK--Y-----------PEYEEHKKHHDWMMQRTRSIRSEYRE-----------KREDLSWELMDFLKKWWLSHIQSNDARYVPYV
---------W------------D--HK-LV------------------------------L------HF--E--LF----------------------HK--H--FI---------------------------------------WLV-HI---D--Y---L
>Geobacter uraniumreducens (Deltaproteobacteria; Desulfuromonadales) 135aa ZP_01143645.1  GI:88938143
----MALITWNDSYSV-KVKQMDEQHKKLVEMINQLHDAMKV------GQGKQVVGDVLNALVSYTKTHFANEESLMKTYG--Y-----------PGYEDQKKAHANLVGQVGDIQKKFLD-----------GNAPLSQDVMTFLKEWLVKHIQGADQKYGLYF
>Geobacter uraniumreducens (Deltaproteobacteria; Desulfuromonadales) 138aa ZP_01140038.1  GI:88934395
-----MSIEWNNNLAT-GVAEIDNQHKELFSRFDSLLSACNE------GKGRDEVLRLLLFLDDYIKSHFAAEERLQLRHN--Y-----------PGYDAHKAQHTRFTEDVDRLESQFRR---------EGATLPLVIQTNQTLVAWLIQHISRIDMEFAAFL
>Helicobacter hepaticus (Epsilonproteobacteria; Campylobacterales) 184aa (1-128) NP_859535.1  GI:32265503
-----MLPEWSDDFSV-HHQIIDEQHQKLFALAHRAYNIANN-----NKSLASDVKNILIEFFDYMKTHFKDEEEYMKAIG--F-----------PQLEEHKKIHRQIVNDMAGMVKNVHS------------VDVLKEMIATIAKDWLLTHILQEDMCYEKYR
>Helicobacter hepaticus (Epsilonproteobacteria; Campylobacterales) 187aa (1-127) NP_859536.1  GI:32265504
-----MLPRWSDDFSV-HHQIIDEQHQKLFALAHRAYKAANG------HVAVNDIKNILIEFFDYMKTHFKDEEEYMKAIG--F-----------PQLEEHKKIHRQIVNDMAGMVKNVHS------------VDVLKEMIATIAKDWLLTHILQEDMRIEKYR
>Hydrogenobacter thermophilus (Aquificae; Aquificales) 131aa BAF34936.1  GI:116234997
------MVIKRADLPQVANSIMNALHEDEIEIINELYEACQK-------GNMERIDELMELLLYDIEEHFLTEEELMREAEF-F------------AYPMHKAEHDGMRKEVKALMESWKK---------NREAQEISNFIKDRLVPWLILHIARWDSTTAIHL
>Idiomarina baltica (Gammaproteobacteria; Alteromonadales) 566aa (429-560) ZP_01041958.1  GI:85710897
LAQQEKLIEWSSQFDV-GIEEINRQHQRLIYIANELYRIQQR------GGDRQAVERLLSSLINYTATHFSYEELLMERHD--Y-----------PDLDAHKHKHQQLVQQVVDFRNRIER------------GDQVVGELLEFVKRWLLQHIQHSDKAYQAHL
>Idiomarina loihiensis (Gammaproteobacteria; Alteromonadales) 832aa (695-826) YP_154567.1  GI:56459286
LSEQDVLIEWSPKLDV-GIEEINRQHQRLIYIANELYRLKLR------DGDQHALQRLVDSLINYTATHFNYEELLMERNG--Y-----------PDLENHKLKHRDLVQDVMRFKHRVDS------------HEDVIDELLEFVKAWLMNHIMKSDMAYKGHL
>Janthinobacterium sp. J3 (Betaproteobacteria; Burkholderiales)  149aa BAC56750.1  GI:28201215
-MDEKTEFAWDDSYLL-GHHAMDTVHREFVDLLQAALTAPNA-----------ELPHALADIARHAEGHFAQENEWMVSQE--F----------P GGDCHIDEHNKVLASIYDVQEKVIQ-----------GDIAIGRKLAQALSEWFPDHASFMDSALAAWL
>Janthinobacterium lividum (Betaproteobacteria; Burkholderiales) 132aa ABK64032.1  GI:118161348
----MGKSHWKDEMAL-GVPVIDEAHEALFQELARLGQLSDQ-----------RFSVAFRELIAAVERDFREEEDLMEQIA--F-----------PSLANHREQHARVLSGLHHAWAAVDE-----------GDLEQGRHALSLLPQWLIFHQATMDLALAAAL
>Kuenenia stuttgartiensis (Planctomycetes; Planctomycetacia) 129aa CAJ72144.1  GI:91202505
-----------MFDYSVKIAEFDGHHKQLIGMINDLDDAMRQ------GKGKIVVGDILKKLINYTGFHFAAEEKIFKQHD--Y-----------PEYVEHKDKHDKMTAKVLALQNDYNA-----------GKVSLTVEVSKFLTDWLNKHIKETDQRYSEHL
---------W------------D--HK-LV------------------------------L------HF--E--LF----------------------HK--H--FI---------------------------------------WLV-HI---D--Y---L
>Kuenenia stuttgartiensis (Planctomycetes; Planctomycetacia) 872aa (734-866) CAJ71928.1  GI:91204275
TLNSLPIFYWRNEYCI-NIQKIDYHHKQLLAKANKLCEAIDS------KKETPILDASISFLIDFVLYHFAEEENLLREHY--Y-----------NDYESHRMKHQRLIEDIMKIKKQISN-----------EDMEMNMEALDLFKDWIIDHILTEDRKYGPYL
>Magnetococcus sp. MC-1 (Proteobacteria; Magnetococcus) 124aa YP_865096.1  GI:117924479
-----------MRMIELGVDFMDAQHQSFVTELSQLAPQPDS-----------ALLLAFPGLVEALTDHFSQEELLMQRCG--F-----------GASQEHRGEHQRLLGQCAMLARFAKR----------GGAPDILAFMRSDLPKWFETHLLTMDAALARHA
>Magnetococcus sp. MC-1 (Proteobacteria; Magnetococcus) 134aa YP_866138.1  GI:117925521
---MPPFLDLPENCRL-NQPDIDLQHSILFALINAVETNNSI-----------ELEVVFKSLVEYVDVHFSYEEKTMLPES--Y-----------GFYHQHLAQHRMFEKKVSEMRSRLTS--------GDQAQEVLRQELRLFLHDWLVDHINGIDRQLFATA
>Magnetococcus sp. MC-1 (Proteobacteria; Magnetococcus) 160aa (1-130) YP_867250.1  GI:117926633
----MKTLSINPAFVT-GLDEIDLQHAFFVDLLARLTPALRN------SKSLPRQVRLLQEIQLFAAFHFHSEENLYREHG--L---------SDHQLTHHQKRHGELLGALNHYIFQLEQ------------EKETVESILAVLGDWFAFHAVEEDGYAVTTL
>Magnetococcus sp. MC-1 (Proteobacteria; Magnetococcus) 249aa (5-142) YP_866733.1  GI:117926116
IIPCKSGTLNFEQDLKLNVPSIDKQHEQLFLLAIGVSHAIQA------GADEESIRVSALELEAYVQEHLSFEERLLASNN--Y-----------PQLNSHKLAHSRFVKTNSELQKSLES-------CSAGTCIKQAPKLFDMLTEWLSDHILRVDRMAVDFM
>Magnetococcus sp. MC-1 (Proteobacteria; Magnetococcus) 253aa (6-142) YP_867005.1  GI:117926388
VPCKAGPLTFEQNLKL-NIPAIDQQHEQLFRLAIGINETLQS------GATEDAVRMNALELEAYVQEHLVYEEKLLSSNH--Y-----------PQQKSHRLAHARFVATSHKIQDELAR-------CSGPACIAKAPQLFDLLTTWLCDHIMNVDRLAVDFM
>Magnetococcus sp. MC-1 (Proteobacteria; Magnetococcus) 320aa (182-316) YP_864332.1  GI:117923715
KIGAAGPCEFPLLGIS----LVDQQHRQLFQLMYALQAQIEN--------RDNRVDLMIMALNNYANKHLADEEALLQRYA--Y-----------PRLADHQKLHIKLREDLVQRAHAVKQ------AADDNEKFKRAEELRDFIQEWLVSHIGSEDRKHCDYL
>Magnetococcus sp. MC-1 (Proteobacteria; Magnetococcus) 779aa (647-777) YP_864533.1  GI:117923916
KDTQPNLMPWNSHLVI-GVAQMDKEHSQLVALINQLYHTILE------GGSSKQGQPVLEALMRYTQDHFKHEEALLKQMH--Y-----------PDLQAHMAEHRALIEQVQHFQQDFER-----------GQASINLRLVGFLKDWLSNHILGSDKPYGTYV
>Magnetococcus sp. MC-1 (Proteobacteria; Magnetococcus) 871aa (733-865) YP_866360.1  GI:117925743
GSENEQVFVWRDAFAI-GVHEMDTHHKKLVEIANSIIEIIGS------NIGKESLMKSMDALIAYTEYHFQAEEKMMEAYG--Y-----------ADLEHHHHLHVKLVSQVVEFQNMVEE-----------KDDYLGLDFEGFFSDWLIQHILNEDRRYAMHL
>Magnetococcus sp. MC-1 (Proteobacteria; Magnetococcus) 926aa (788-921) YP_867064.1  GI:117926447
ANAIEPKIRWLSSYEV-GIETIDSDHKRLVGMMNQLYAAMKT------GSSSQVMEELFDGLINYSVVHFQREERLFEQHR--Y-----------PDAAGHSKRHEAFKTYVLGKRDEFVA----------GSNIHLSQDIFTYLEEWLINHIIHDDMAYVPYL
---------W------------D--HK-LV------------------------------L------HF--E--LF----------------------HK--H--FI---------------------------------------WLV-HI---D--Y---L
>Magnetococcus sp. MC-1 (Proteobacteria; Magnetococcus) 927aa (789-921) YP_867464.1  GI:117926847
VGAYAPAIAWRNEYSV-GVQLLDADHKQLINLINELSIAVKD-------DRENFLQHVYERLVNYATTHFAREERMMAEIG--Y-----------SALPSQKAEHERFLSTVGSKRDILLN----------GADPKVATEIIDFLKDWLVGHIMKSDMAYKPAF
>Magnetococcus sp. MC-1 (Proteobacteria; Magnetococcus) 941aa (802-935) YP_866832.1  GI:117926215
```

```
CGTGEEFFPWNDILLT-NVKEVDIQHKELVRMVNALHQEITS-----GQGGKQKLRTMVDGLIAYTVNHFKDEEAFMRRNH--F-----------PGLQAHLPEHIKFTDAVVELVAQVDK-----------GEAYIDMDLTGLLRDWLVNHIMGTDRKYAEHF
>Magnetococcus sp. MC-1 (Proteobacteria; Magnetococcus) 994aa (856-988) YP_864243.1  GI:117923626
TINDQKMIDWEDALDV-GVKVLNADHLKLMNYINDLHAAMRD------GKGKDKLATILAGLIEFTHTHFAREEGLMKKHG--Y-----------DRYAQQKEEHVNLIAQLGEKKKQFDE-----------GSATVALDLLAFLQNWLIDHIKGEDMRYKTFF
>Magnetococcus sp. MC-1 (Proteobacteria; Magnetococcus) 997aa (863-995) YP_865500.1  GI:117924883
SIDRSTFLKWNDGYST-GNAQIDGEHQQLMNIINELYIKLWS------GEKNPDYNPILLRLDGYTRDHLRREEELLASKS--A-----------PGLDGHKSIHREFIKKLEAFKLAFDQ-----------GNVGLSMEMMAFLKNWLSQHIVKVDKVIFKKI
>Magnetococcus sp. MC-1 (Proteobacteria; Magnetococcus) 1032aa (894-1031) YP_864127.1  GI:117923510
VDAEPIAVVWKPNMSV-GVQIFDQDHRRLVSYINRLELAHRA------GQSKTALERVLRALTDYTLIHFRREEKMMTESN--Y-----------PGVQAHKAQHTKLEDQVADYNRRMLQ-----------EESHITEEVLTFLKGEFLDHIMRTDHEYSDHF
>Magnetospirillum gryphiswaldense (Alphaproteobacteria; Rhodospirillales) 149aa (1-127) CAE12026.1  GI:33945221
------MFDFTDDLSV-GNEAIDADHRAFFEVARLIHESVHG------GKHDLIILSALLMLDEYVEGHFYREEKAMKAVG--Y-----------PRLAGHRHRHRVFRGRVRAIVEAYRE-----------GTMSVADNLSTLVVQWLRSHITNDDLQYKTWI
>Magnetospirillum gryphiswaldense (Alphaproteobacteria; Rhodospirillales) 159aa (1-136) CAJ30087.1  GI:78033471
------MFDFTDDLSV-GNEAIDADHRAFFEVARLIHESVHG------GKHDLIILSALLMLDEYVEGHFYREEKAMKAVG--Y-----------PRLAGHRHRHRVFRGRVRAIVEAYRE-----------GTMSVADNLSTLVVQWLRSHITNDDLQYKTWI
>Magnetospirillum gryphiswaldense (Alphaproteobacteria; Rhodospirillales) 524aa (197-329) CAJ30107.1  GI:78033490
SVPVKPDFGWSKAIET-GRNDIDSQHRGIIDIMNDLNEVPDT-----EDGDGTKVERALIALKDYVKIHFSHEEQLMDSFD--Y-----------DDRKRHKKLHSAFVAKIETLKAGSLT------------SPDVRRKLLGIVYNWLMSHITCVDRIMIAQL
>Magnetospirillum magneticum (Alphaproteobacteria; Rhodospirillales) 122aa YP_419883.1  GI:83309619
---MGMIAIWDNRYSV-GHARVDAEHRQVISLLNEIDVARSV-----GAPPVVVEMALETLLRTIVAHFARDEVPQGDHMA-I------------IASARRLLDGWRGGKPEALERRALV-----------------------NLARRWIGHMGRREASLPSQR
---------W------------D--HK-LV-------------------------------L------HF--E--LF-----------------------HK--H--FI--------------------------------------WLV-HI---D--Y---L
>Magnetospirillum magneticum (Alphaproteobacteria; Rhodospirillales) 127aa YP_421315.1  GI:83311051
------MATWLDEQWKSGDPAIDAEHQKLHQMISSMSAVIRN------DPGLGLAIEAVDVLTERIRIHFRMEGLAAR-----L---------NAAAAEQLKLDHQRLLRLLTPLYDALRR-----------GSAEQAKLLLSDFLSQLDAHDREMDIPLFQK-
>Magnetospirillum magneticum (Alphaproteobacteria; Rhodospirillales) 129aa YP_419932.1  GI:83309668
----MSIIAWTEAMSV-DNPALDKDHQKLIRMINELDQSAPD------------FLELFNAVLDYTTGHFDREETHLDAIG--F-----------PGLDAHRVQHDDFADQVAAMLKEYRE----------RPFDAGDTRLKDFLWSWLKGHILIEDQRYAAWT
>Magnetospirillum magneticum (Alphaproteobacteria; Rhodospirillales) 132aa YP_420778.1  GI:83310514
------MPAWTESLSV-GVPVLDEHHRHLFHLLATLDGMAGG------SVGQDAVRSVFDDLNGYIAYHFAEEEAMMARAD--F-----------PFLELHRHSHQTIAMRVADMSAELSM----------ANVERVIRELHDFLVGWLVHHIEIEDFEYRPFL
>Magnetospirillum magneticum (Alphaproteobacteria; Rhodospirillales) 134aa YP_420912.1  GI:83310648
----MSLIEWDPSFSV-GSARMDADHQKLISLLNRLYDAWHG------GESIAELGWLFDELLAYADTHFAAEEMALKSRN--Y-----------PRLEKQQEDHLRLKESVMAFRDRHLA---------GEMPDALTTEMTAFLKAWLLEHILGEDMQYRSYF
>Magnetospirillum magneticum(Alphaproteobacteria; Rhodospirillales) 135aa YP_419589.1  GI:83309325
----MAYLQWTENLSV-GIPRMDEHHKKLVELINQVFDAMSG-------DAASAVDSVLADLLDYTRYHFSEEEKLLAACA--Y-----------PDLAEHQEVHRSMVKEVLDMRSRHLA----------DPASVTASETLDFLSKWLMRHIIGKDLRYRPYA
>Magnetospirillum magneticum (Alphaproteobacteria; Rhodospirillales) 136aa YP_423659.1  GI:83313395
---MTAQISWSDDLLI-NVAAIDSDHKKLFDLMSEIFATSAH--------GADAINRAIGALAKYTKEHFAREEASMAKTN--Y-----------PGLSKQMYEHEHLVFTLESMMDRLMI----------SGPEAIDADLAKFLMSWLGDHIMDFDLKYAEYL
>Magnetospirillum magneticum(Alphaproteobacteria; Rhodospirillales) 138aa YP_423534.1  GI:83313270
-----MSIAWNEALAI-GDPIIDADHRRMIELIARLEVAAGL------EIDCAGIGRTLRDLTDLCREHFAREEALQHAVG--F-----------PETESHRTAHEMLMKRLDAVLAHFTD-------GCDEVRSGIVRTLGDSLATWLVSHIVNSDMEFKPYV
>Magnetospirillum magneticum (Alphaproteobacteria; Rhodospirillales)138aa YP_420035.1  GI:83309771
----MSTDFWVDALSV-GVDEFDRAHRQHLNTIGEIEVALSE-------NRRSDAVGLLTHLLQILTVHQAEELDLLVRTS--Y-----------PGVDQIREVQAANLARLHRLKDQVEG---------QTSQLADARRVATEMRMAFVDYLLKGDINFKSHL
>Magnetospirillum magneticum (Alphaproteobacteria; Rhodospirillales) 140aa YP_422017.1  GI:83311753
-----MAIAWEETLKV-GDIEIDADHKELIGLINDFEAKAKA----PEGVDKHVIQVTLERLQLYAYDHFAREEYIQAVAK--Y-----------DGLEENKRQHAALRKTLGDYIAKFNA-------GEYADLKVAAGEMSGFLNHWLMNHILETDLKMKGKM
>Magnetospirillum magneticum (Alphaproteobacteria; Rhodospirillales) 140aa YP_423499.1  GI:83313235
SGNALWQYRWSEEFAL-GDPLVDAQHRAFFEEAAALRAALAS------EEPKDEIVAYCSAFITNLRTHFADEERLMARIG--F-----------PALPIHQQEHERLLARTESVVAEIHA----------ADCLLDCLLGAHALMEALVEHVTQQDMRIKTFV
>Magnetospirillum magneticum (Alphaproteobacteria; Rhodospirillales) 142aa YP_421602.1  GI:83311338
------MANWQEHMRT-GIDVVDDDHQELFALVGQFETASKT---ASGQVDGAEIGRILERLQAYVNEHFEREETAQLESG--Y-----------EGYAENKRQHDELRRTLETFLGKHRR-------GEWGDLKVATESMRSFLAIWLSEHIMKTDRKMRGRI
---------W------------D--HK-LV-------------------------------L------HF--E--LF-----------------------HK--H--FI--------------------------------------WLV-HI---D--Y---L
>Magnetospirillum magneticum (Alphaproteobacteria; Rhodospirillales) 143aa YP_421612.1  GI:83311348
----MTVIEWSNELKL-GVPAMDAEHRHLLKLTNDFLTAARE------NAAFARLTLILGELIARTRIHFQAEETLLDRAG--Y-----------PGLAGHRAEHGRLLVEAQRLYERFQT-LDAAPEHDDLASRTLTLEAAKFLQRWLVDHILADDRPYRPYV
>Magnetospirillum magneticum (Alphaproteobacteria; Rhodospirillales) 144aa YP_423628.1  GI:83313364
------MLIWRDAMAI-GQPEIDRARKHVIGRINDFERALGA------HGPHTALGLFLTGLYEETSTAFGREEKIQRECS--F-----------PFTEMHHREHAAILEKVELLKEQYDE------LDARGNFTPLLRELAGLAKEWITAHIVQSDLKLRPYW
>Magnetospirillum magneticum (Alphaproteobacteria; Rhodospirillales) 146aa YP_420100.1  GI:83309836
MIPRHAVIRWGEPLLL-GVGFVDHDHCEAVEMINRLAAAAPT-----------ERLDLARAFAHHCVEHFAREEEMMVKTGF-F------------ALDPHRDEHIRVVAQLEEVIRALEA------------GDPCDDYFTVELPQWFLEHRATMDYVTSGFA
>Magnetospirillum magneticum (Alphaproteobacteria; Rhodospirillales) 147aa YP_422454.1  GI:83312190
----------MATSYTIGHKPLDDDHDRMIAAWRELEGSRTL----------EAAKAAAAVLMAEAGEHFAREEMFMLQCG--Y-----------PDRVRHMALHAEMASGLRRVLLSPLM--------GGSPHDDFVLAVRSLMDKWVMVHILGEDAKLAPYA
>Magnetospirillum magneticum (Alphaproteobacteria; Rhodospirillales) 148aa (7-148) YP_423329.1  GI:83313065
VSWGNEKIEWSDDLLL-QAEQDDEDHREMFALMNRVFSAARL--------GADVVTSAVADLCLFTREHFTREQHSMESAG--Y-----------PDCEAHRYDHEYLIFQLDVLIDRLML----------SGPELVAEELVDLLKRWLCDHILTYDSAFVEFL
>Magnetospirillum magneticum (Alphaproteobacteria; Rhodospirillales) 156aa( 8-142) YP_423198.1  GI:83312934
AGDPMTSIAWSEAFSV-GNSLLDSDHRILFDLLTQLFDATDT------GQSRDVVGSVLSALSEYAEHHFRREEALLAASG--Y-----------PDIEIHRAEHRRLEDHVRDISGRYRA----------GERGVLDEQVVELLKKWLTQHIQVTDKSYKPWV
>Magnetospirillum magneticum(Alphaproteobacteria; Rhodospirillales) 157aa (5-138) YP_420298.1  GI:83310034
GAARGAIPEWTPEMSV-GNDTLDSDHKAFFDIARLLYDSLAD-----KRDQGLIIASTLLILEEYVDGHFLREEKALKAVG--Y-----------TQLSEHHHKHRKFQARVRAISETYQS-----------GTKSAADDLPDLVVKWLTHHILNEDMQYKRWI
>Magnetospirillum magneticum(Alphaproteobacteria; Rhodospirillales) 161aa (1-132) YP_423764.1  GI:83313500
-----MTIAWREAMSV-GDETIDADHRHLVDMINVFEAAVVG------KIDHKRVARVLLGLVEYTGAHFKREEEIQVLIR--Y-----------PYHDSHRHGHRDVLKQLTGIAQQYTQ-------ATGTARDEMIRGLGGFLREWLVDHIIQSDLRMKPYV
>Magnetospirillum magneticum (Alphaproteobacteria; Rhodospirillales) 169aa (31-162) YP_420741.1  GI:83310477
-----MTIAWREAMSV-GDETIDADHRHLVDMINVFEAAVVG------KIDHKRVARVLLGLVEYTGAHFKREEEIQVLIR--Y-----------PYHDSHRHGHRDVLKQLTGIAQQYTQ-------ATGTARDEMIRGLGGFLREWLVDHIIQSDLRMKPYV
>Magnetospirillum magneticum (Alphaproteobacteria; Rhodospirillales) 171aa (13-148) YP_420348.1  GI:83310084
MNQTNPLPEWTDDLSV-NVEIIDEDHQAFFRLAALMSDVTST----PDNDQPYLIETSINILEEYIEGHFLREQKAMAKAD--Y-----------AYLVEHIAAHDAFADRVTRIIQDYRQ----------TGSIEKIGTLAHLVTDWLNNHIRVVDAQYKGIL
>Magnetospirillum magneticum (Alphaproteobacteria; Rhodospirillales) 171aa (21-158) YP_422163.1  GI:83311899
TRSEGIMLVWREAMST-GHSEIDKAQRQLIAHINEFETALRK------SGPDVAMGLFLSGLYDQVAINFGREEQIQRESS--F-----------PFLDAHHREHGNLLGRLASIQNHYQS------ANHRADQGALLRDLTALIRDWVSHHMVQSDVMLRRYL
---------W------------D--HK-LV-------------------------------L------HF--E--LF-----------------------HK--H--FI--------------------------------------WLV-HI---D--Y---L
>Magnetospirillum magneticum (Alphaproteobacteria; Rhodospirillales) 179aa (31-160) YP_422102.1  GI:83311838
KAERLHWDFWSEELAI-GHAAIDDDHKRILAGIERLAKAFQL------GDGDAALSDGLALIVDYSRHHFEREERMLAAAG--Y-----------AHLEHHRARHQSFCDYVAHASKADGG--------------IDHGALLSYLVDWWVGHIASEDKLYRPAL
>Magnetospirillum magneticum (Alphaproteobacteria; Rhodospirillales) 275aa (1-134) YP_420086.1  GI:83309822
----MAMLELTDDLRI-GHPVIDADHQRLIEIINEFLEHSKA------ADNSQVLHETLKSLLAYGRDHFSREERIQRQCL--Y-----------PLQPGHFHEHKALLDQVREIARAYFI------AKTKPLNESSVLEVNELLKAWLIGHVKKFDTGMRDWV
>Magnetospirillum magneticum (Alphaproteobacteria; Rhodospirillales) 282aa (1-129) YP_421898.1  GI:83311634
-----MGVTWSERMSV-GVPLLDSDHKTLIGLINHLQRSIGD------DEEHASVGSVLQALEEYAAHHFAREEAMMDVCR--Y-----------PLLSRHHGTHAEFTKKVTDLKGRYDLD----------RTGVRARECLAFLNSWLIDHICTTDMNYRSWV
>Magnetospirillum magneticum (Alphaproteobacteria; Rhodospirillales) 499aa (1-131) YP_422973.1  GI:83312709
------MIVWRDAMSV-GAPALDADHKRLIDLINLTEQWIGQ-------DNWRQVATITEELLRYVDEHFRREEAVMAAIK--Y-----------SELEQHKKAHETLAHKAKLLHDKFKA------ATLEEDLKTCSQVLIRVLTDWLVTHILKEDMKYKGSV
>Magnetospirillum magneticum (Alphaproteobacteria; Rhodospirillales) 502aa (14-150) YP_420234.1  GI:83309970
CWGCAMAVQWQEAMSI-GVADLDAEHRAMLELLNSLEAGLEG-------GDDDGVADAMRDLVGLMSEHFSREEDLLSGIG--C-----------PSFYAHRDGHDAIADRIHLLRRRYLV------AVDSGARRDVASALLNFTRISLLDHILTEDHAMGRRM
>Magnetospirillum magneticum (Alphaproteobacteria; Rhodospirillales) 665aa (527-659) YP_422630.1  GI:83312366
DQSDVKLLNWDNALNM-GIGNIDRHHREMFDEVNRLYAKMSQ------GNGAEAGQGMITLIDTVMGSHFREEEEVMSRNS--Y-----------PHLDEHRRHHRDFLDRAKSHKGAVQS-----------GDPTAAAKMFEYVATWLTNHIQMEDGKLANYL
>Magnetospirillum magneticum (Alphaproteobacteria; Rhodospirillales) 795aa (656-788)) YP_423519.1  GI:83313255
EKKDMVLLHWDRDLEA-GVTSIDRYHQEKFEHINEFYRQMMS------EDGGKAAIALLSEIDRSIQGHFGEEEALMAKCG--Y-----------GDGEAHRRSHKAFQERIRALRTGIET-----------NHPEAVSQLFDYVSDWFPDHIRNEDMALALFL
>Magnetospirillum magneticum (Alphaproteobacteria; Rhodospirillales) 807aa (668-800) YP_419583.1  GI:83309319
DKADMRILEWENALNT-GESSIDRHHREMFDQVNRFYGEMMSG------NGDAAIQGILGLVPAAFVPHFKEEEELMTRHA--Y-----------PGIDEHRRRHREFMDKFEGLKRAVET-----------GKDGANGQLFQFVAGWLKDHIRHEDGKIAAFL
>Magnetospirillum magneticum (Alphaproteobacteria; Rhodospirillales) 810aa (673-803) YP_423156.1  GI:83312892
DKKDMKLLVWDGALNT-GVASVDRHHQAMYDQVNEFYRQMMS------GDGSRAAAAMLAELNRSIQDHFTEEEALMDKTR--Y-----------PAAEDHRRNHQAFLDRLAGMKTGLET-----------NRAEAPAEMFEYVSTWLRGHIGNDDRALGLHL
>Magnetospirillum magneticum (Alphaproteobacteria; Rhodospirillales) 870aa (733-864) YP_422458.1  GI:83312194
QVVSSTDLVWRDEYSV-NIQRIDNHHRRLFEMSNRLIEAVRL------SKGKAEIGEALDFLMGYAMYHFSEEEALLERYG--Y-----------PEGAGHASRHRRLMAQARELEEHLAS------------AAISADEVLTFLQDWIVNHILMEDRRYAPFI
---------W------------D--HK-LV-------------------------------L------HF--E--LF-----------------------HK--H--FI--------------------------------------WLV-HI---D--Y---L
>Magnetospirillum magneticum (Alphaproteobacteria; Rhodospirillales) 906aa (778-900) YP_421045.1  GI:83310781
GEHARVLIKWHDEYSV-GVQRIDSHHRRLFEIGNSVLDMMER------KSPDAEVAETMGLLREYARFHFSEEEGLLIRYK--Y-----------PDLSSHRARHNRLLEQLTELQAAVAE-----------GRGYNEAEVLTFLQEWIVGHTLAEDRKSAIYL
>Magnetospirillum magnetotacticum (Alphaproteobacteria; Rhodospirillales) 120aa ZP_00053268.2  GI:46202398
---------------------MDAEHRVQVGLISALERAVEA------RAPQAEIQALVEQLIDYTDVHFMSEQLLMRLAA--Y-----------PDIHGHEAEHDGLMEQLRRIQSGFDL-----------GDSDVVMAETQVLRNLLIEHIRTHDLVFAQYV
>Magnetospirillum magnetotacticum (Alphaproteobacteria; Rhodospirillales) 120aa    GI:23014047
-----MIAIWDNRYTV-GNALADAEHRQVISLLNEIDVARSV------GAPPEVIGKALETLVRSIESHFAGDQPPQREHAA-I------------IMSARRLLTSWQSRAPGAIERRALI----------------------NLARRWFDHMGRNERVMTSQR
>Magnetospirillum magnetotacticum (Alphaproteobacteria; Rhodospirillales) 122aa ZP_00054990.2  GI:46201470
--------------------MIDEQHRRLFALAGRLTLDASA-----NDKDEAGGEAVLADLLDYTHSHFRDEEEFMRLIQ--Y-----------PGLRVHQQAHASIFAGVDQLLARMRT----------VDRQIMRREIGYFISEWLVRHVLHDDLQYTRYI
>Magnetospirillum magnetotacticum (Alphaproteobacteria; Rhodospirillales) 127aa ZP_00053732.1  GI:23013881
------MATWLEDQWKSGDSTIDTEHLKLHEMIRSMTAVMRN------DPGTGLAQEAVDVLTERLRIHFRMEESLAAKAN-------------PEAIDTLKQDHQRLLRLLTPVRDAVQS-----------GSAEKAKSLMTDFAEQLDKHDREIDIPLFRK-
>Magnetospirillum magnetotacticum (Alphaproteobacteria; Rhodospirillales) 132aa ZP_00053735.1  GI:23013885
------MAEWTESLSV-GVPKLDEHHRHLFHLLDRIDGLAEA------GLAQDDVRSVFDELNNYIAYHFSEEEAMMERAG--F-----------PFLDLHRHSHQTIAMRVAEMSATLSV----------ANVERVVRELHDFLAGWLVHHIEIEDFEYRPFM
>Magnetospirillum magnetotacticum (Alphaproteobacteria; Rhodospirillales) 134aa ZP_00054355.1  GI:23014545
MIARHAVIRWGEPLLL-GVGFVDRDHCEAVEMINRLAAASGA-----------ERLELARAFAHHCAEHFAREEEMMVKTG--F-----------FALEPHRDEHMRVLGQLDEVIRSLEG------------GDSCAEYFAVDLPQWFLEHRATMDYVTSGFA
>Magnetospirillum magnetotacticum (Alphaproteobacteria; Rhodospirillales) 134aa ZP_00051444.1  GI:23010917
----MSLIEWDPSFSV-GSARMDADHQKLISLLNRLYDAWHG------GEDIAELGWLFDELLAYADTHFAAEEMALKSRN--Y-----------PRLEKQEQDHVRLKDSVIAFRDRHLA---------GETPDALTTEMTAFLKAWLLEHILGEDMQYRSYF
>Magnetospirillum magnetotacticum (Alphaproteobacteria; Rhodospirillales) 135aa ZP_00207881.1  GI:46200880
----MAYLQWTENLSV-GIARMDEHHKKLVDLINQVFDAMSG-------DASAAVDSVLADLLDYTRYHFAEEEKLLAACA--F-----------PDLEEHQAVHRSMVKEVMEMRSRHLA----------DPASVTASETLDFLSKWLMRHIIGKDLRYRPFA
---------W------------D--HK-LV-------------------------------L------HF--E--LF-----------------------HK--H--FI--------------------------------------WLV-HI---D--Y---L
>Magnetospirillum magnetotacticum (Alphaproteobacteria; Rhodospirillales) 135aa ZP_00052929.2  GI:46202606
--------------MRTGIDVVDDDHQELFALVGQFETASKT---ASGLVDGAEIGRILERLQTYVIEHFEREETAQLETG--Y-----------EGYEENKRQHDELRRTLEKFLDKHNK-------GEWGDLKVATESMRSFLSIWLSEHIMKTDRKMRGRI
>Magnetospirillum magnetotacticum (Alphaproteobacteria; Rhodospirillales) 136aa ZP_00055246.1  GI:23015471
-----MSIAWSEALAV-GDPSIDADHRRMIELIAQLEAAVCS------EIDCAAVGRTLQDLADLCRLHFAREEELQASVG--F-----------PETESHRTAHEMLLKRLDAILAHFAD-------GCDEVRTGIIRTLGDSLATWLVSHIVNNDMEFKPYV
>Magnetospirillum magnetotacticum (Alphaproteobacteria; Rhodospirillales) 139aa (1-122) ZP_00054325.1  GI:23014513
---------MAATYSI-GQPALDDDHDRMIAAWRELEASRTL----------DAAKTAAAVLMSEAAEHFAREEQFMLQCG--Y-----------PDRVRHTAMHAEMASGLRRVLLSPLM--------GGGKHEDFVGAVRNLMDRWVRVHILGEDTKLAPYA
>Magnetospirillum magnetotacticum (Alphaproteobacteria; Rhodospirillales) 140aa ZP_00052752.1  GI:23012741
-----MAIAWDEALKV-GDIEIDADHKELIGLINDFEAKAKA----PEGVDKHVIQVTLERLQLYAYDHFAREEYIQAVAK--Y-----------EGLEENKRQHAALRTTLGTYIEKFNA-------GQYADLKVAAGEMSAFLNHWLMNHILETDLKMKGKM
>Magnetospirillum magnetotacticum (Alphaproteobacteria; Rhodospirillales) 142aa ZP_00055707.1  GI:23015944
------MFLWNMSMET-GIETVDQGRKRVLAAMADFFQGMDD-----------PALNQQILAARTGAIFNAMKTAFAAENA-Y-----LAEREAPDQEKHQANHAALSAAFIDLCRKLIP------KIKNHKQAQQVCLEIYQLVDDAIFHHITKEVGNYRDL
>Magnetospirillum magnetotacticum (Alphaproteobacteria; Rhodospirillales) 144aa ZP_00055499.1  GI:23015731
---------MLIWRDAMAIGQADIDHARKHVIGRINDFERA----LGAHGAHTAMGLFLTGLYEETSTAFGREEKIQRECS--F-----------PFTEPHHREHAAILEKLELLKERYDET------DARGDFTPLLRDLAGLAKEWITVHIVQSDLKLRPYW
>Magnetospirillum magnetotacticum (Alphaproteobacteria; Rhodospirillales) 146aa (7-138) ZP_00055962.2  GI:46201047
VSWGNHKIEWSNDLLL-HMEQEDGDHREMFVLMNRIFSAAQ--------IGADMVTQAVADLCLFTREHFLREQHGMERAG--Y-----------PDREAHRYDHEYLIFQLDVLIDRLMLS----------GPECVAEELVDLLKRWLCDHILTYDQAFAAYL
>Magnetospirillum magnetotacticum Alphaproteobacteria; Rhodospirillales) 150aa ZP_00052625.1  GI:23012573
KAERLHWDFWSEELAI-GHAVIDDDHKRILAGIERLAKAFQ------LGDGDEALRDGLALIVDYSKSHFEREERMLAAAG--Y-----------PHLEHHRARHQSFSDYVAHASEADGGI--------------DHGALLSYLVDWWVGHIASEDKLYRPAL
>Magnetospirillum magnetotacticum (Alphaproteobacteria; Rhodospirillales) 158aa (1-140) ZP_00056082.1  GI:23016325
----MRTMVWSEHFAL-GHDRIDREHRRLLELSAQIEARAE------TTTDLDDILPLCRDFFRLLVAHCFYEEQLLRK----L-----PRDRFGSHVDEHCRGHARLIKQAQTVLTGQVPS-------------GFESLQGFFGAYFELMHDLLVDDTELVGS
>Magnetospirillum magnetotacticum (Alphaproteobacteria; Rhodospirillales) 159aa (1-136) ZP_00053162.2  GI:46202468
MNQTNPLPEWTDDLSV-NVEIIDEDHQAFFRLAALMNDVTA----TPDNDQLYLIETSINILEEYIEGHFLREQKAMAKAD--Y-----------AYLVEHIAAHDAFADRVTRIVQDYRQT----------GNIEHIGALAHLVTDWLNIHIRGVDAQYKGIL
---------W------------D--HK-LV-------------------------------L------HF--E--LF-----------------------HK--H--FI--------------------------------------WLV-HI---D--Y---L
>Magnetospirillum magnetotacticum (Alphaproteobacteria; Rhodospirillales) 179aa (31-160) YP_422102.1  GI:83311838
KAERLHWDFWSEELAI-GHAAIDDDHKRILAGIERLAKAFQ------LGDGDAALSDGLALIVDYSRHHFEREERMLAAAG--Y-----------AHLEHHRARHQSFCDYVAHASKADGGI--------------DHGALLSYLVDWWVGHIASEDKLYRPAL
>Magnetospirillum magnetotacticum (Alphaproteobacteria; Rhodospirillales) 272aa (1-131) ZP_00053965.2  GI:46202007
------MLELTDDLRI-GHPVIDADHQRLIEIINEFLEHSN-------EVNPQILHETLKSLLAYGREHFAREEGIQRQCL--Y-----------PYQQGHSNEHKSLLDQVREIARAYFVA------KTKPLNATSVQDVNELLKAWLIGHVKKFDTSMRDWV
>Magnetospirillum magnetotacticum Alphaproteobacteria; Rhodospirillales) 274aa (1-121) ZP_00054151.2  GI:46201886
--------------MSVGVPLLDSDHKTLLGLINHLQRSIG------DDEEHAAVGSVLQALEEYAAHHFAREEAMMDACR--Y-----------PLLTRHHGTHAQFTEKVLGLKSRYDQD----------QTGVRARECLAFLNSWLIDHICTTDMNYRSWM
>Magnetospirillum magnetotacticum (Alphaproteobacteria; Rhodospirillales) 500aa (1-131) ZP_00054349.2  GI:46201789
------MIVWRDAMSV-GAPALDADHKRLIDLINLTEQWIG-------QDNWRQVATVTDELLRYVDEHFRREESVMAAIK--Y-----------PEIEQHKKAHETLAQKARLLHEKFKAA------TQDEDLKTCSQVLIRVLTDWLVTHILKEDMKYKASI
>Magnetospirillum magnetotacticum (Alphaproteobacteria; Rhodospirillales) 699aa (560-692) ZP_00056481.1  GI:23016728
DKKDMKILVWEGALNT-GVASVDRHHQKMYDQVNEFYRRMM------SGDGFAAAIAMLDELNRSIRDHFNEEEALMSKAN--Y-----------PGADEHRRNHRAFLERLASLETSLDAN-----------HPEAASQMFDYVSTWLREHISKDDKALGNHL
>Magnetospirillum magnetotacticum (Alphaproteobacteria; Rhodospirillales) 807aa (668-800) ZP_00056272.1  GI:23016517
DKNDMKILVWDNALNT-GESSIDRHHREMFDQVNRYYGEMM------SGNGDVAVQGILSLVPTTFVPHFKEEEDLMSRRG--Y-----------PGIDEHRGRHREFLDAFESHKRAVEAG-----------KDGASGALFQFAATWLKDHIHYEDGKIAAFL
>Magnetospirillum magnetotacticum (Alphaproteobacteria; Rhodospirillales) 808aa (670-808) ZP_00055928.1  GI:23016169
```

```
DKKDMTLLHWDATAET-GEATVDKHHRDLFELVNKAYRQMM------SGEGSQMAVPLLAELDRTMRSHFTEEESLMSRSA--Y----------AEIETHRRSHHAFFERVDPLRAAIAAG----------SAEAGGELFDYVATWLSQHIRKDDKALAAFL
>Magnetospirillum magnetotacticum (Alphaproteobacteria; Rhodospirillales) 870aa (733-864) ZP_00054329.1  GI:23014517
QVVSGGDLIWREEYSV-NIQRIDNHHRRLFEMSNRLIAAVR------LGKGTAEMSEALDFLKGYATYHFSEEETLLERYG--Y----------PEEAGHAAKHRRLLAQLHELTERLATT-----------TVTAGDVATFLQDWIVNHILIEDRRYAPFI
>Magnetospirillum magnetotacticum (Alphaproteobacteria; Rhodospirillales) 889aa (751-883) ZP_00055643.1  GI:23015878
GEHARVLIKWHEEYSV-GVQRIDSHHRRLFEIGNSVLDMME------RKTDDAEVAETMGLLREYARFHFSEEEGLLIRYK--Y----------PELSAHRVRHARLLEQLTELQAAVAEG----------RGYNEAEVLTFLQEWIVGHTLAEDRKSAIYL
>Marine gamma proteobacterium HTCC2207 (Gammaproteobacteria) 174aa (29-166) ZP_01224113.1  GI:90416181
ALKRLLDKALALKLDSALKSETKSPEPEALGEFETEEEAKT------SQSDSTKIELLMQTLLLDTVTHFKLEEQCMEQ----Y---------EFPAREAHSQEHRLVLQWMDREHSLWSRD------CNIETINHLSIYAADRFPKWLLNHIVTMDTVMASYI
---------W-----------D--HK-LV------------------------------L------HF--E--LF----------------------HK--H--FI-------------------------------------WLV-HI---D--Y---L
>Marinobacter aquaeolei (Gammaproteobacteria; Alteromonadales) 662aa (5-138) YP_960352.1  GI:120556001
VPNAFEIFPWNRFET-GIDDIDQQHRVLVNILNRLAWHFA------SDTSELTPDQILDELLTYAAFHFYEEEAIWNQT----L-------GEAAMARNHHDAHQMFFAQVEELRNSREPQ-----------EEVLTRLFDFLTRWLAFHILESDRRMALTA
>Mariprofundus ferrooxydans (Proteobacteria; Mariprofundus) 143aa ZP_01451258.1  GI:114775690
MQDQVWQLEWNDKLSV-GIDDIDVEHRHFIELINGLNRAIL------DRMDISEIRRRMQLILDDAAQHFAHEEALFAAWH---Y----------PDADEHAKYHRQATAAFQEIMARLSDK---------HTESEWIEAGLKVKQLLVEHLITEDMKYRDYF
>Mariprofundus ferrooxydans (Proteobacteria; Mariprofundus) 373aa (3-132) ZP_01451244.1  GI:114775676
----MVAFQWDSNFIT-GVADVDQQHMHLVDLINRLGEHLA-----GNDIALDYIEEIYQELAAYAHYHFQCEEELMQEVG---L--------DQRHQDFHRKEHLDFLHEVTSMHDGISAD----------SPDTSSYLMEFLTHWLAYHILGADQNMARQM
>Mesorhizobium sp. BNC1 (Alphaproteobacteria; Rhizobiales) 134aa YP_665917.1  GI:110347099
----MALIEWKSHYSV-GVDAVDHEHRELIDLINQLHENLL------AGSPDLMVTAFLGEIFRAISAHFALEERFMREHG---Y----------DQFAEHKQAHEQLLDEIRDIMDGYEAD----------PEGTSQQLSDRLDAWFTLHFRTHDARLHHRL
>Methylococcus capsulatus (Gammaproteobacteria; Methylococcales) 131aa YP_113225.1  GI:53805087
----MALMTWTAAEFGTNVGFADDQHKTIFDMVNKLHDTAA------TGNRSEIGKQLDALIDYVVMHFKSEETEMQKKG---Y----------ADFAAHKAEHDKLVGVCADLQKKFHAG----------EAEVNQDTTRFVRDWLVNHIPKVDKLYGPCL
>Myxococcus xanthus (Deltaproteobacteria; Myxococcales) 185aa (7-139) YP_635509.1  GI:108758665
TQVVCFLVHAECSGMGSPFDILIDQHRELEERLERLASGVE---------PDELRGLTAELLALLRLHSRLEERCLYPVMAG-V-------EGRESCREQTEDHLTMRELMAELEELPSGH------------PEWQARLLTLEDLAVAHFQEEENARLPQL
>Oceanospirillum sp. MED92 (Gammaproteobacteria; Oceanospirillales) 121aa ZP_01167458.1  GI:89094519
------------MPLTSELIWQDQHQVLFDLIDEIKADHV-------------SKSTFVRLHDYAEHHFCIEEAYMKALE---Y----------PEYENHLLAHNKFRTELKTMLDEHHSY----------DAMLRDALSDFLSEWLKHRIFGVDKKLETFI
>Oceanospirillum sp. MED92 (Gammaproteobacteria; Oceanospirillales) 129aa ZP_01165824.1  GI:89092872
----MRNIVWDKKFEL-GHEKIDFEHRIFLDLIRSCSLLDE---------DRLRSSRLLREVEKYADFHFLSEENLMLDIN---Y----------PDYEEHRAEHEYLLAQLKEKCFEYKHH-----------DIELSELTGFLFEWFALHTTQVEAKLTRYI
>Oceanospirillum sp. MED92 (Gammaproteobacteria; Oceanospirillales) 135aa ZP_01166597.1  GI:89093650
-MANQLNPSVIPQVAL--DFMNE-DHGEAVELVNQLLIAIE-------SDSSTKVSELLKAFFEHNQEHFAREEEQMIRVN---F----------PPYSCHKGEHERVLEEMKTVISEWEAS---------EDRSALQSYLTDTVANWLVNHISTMDTVTAMFV
>Pelobacter carbinolicus (Deltaproteobacteria; Desulfuromonadales) 135aa YP_355938.1  GI:77918123
----MALIQWNTSLSV-NIAEIDTQHQRLISMINELNDAML------QGRGKDVLGKIIAGLVSYTATHFATEEKYFDRHG---Y----------PASNAHKQEHRDFTAKVSEFKQGFDEG----------RLGLSVSVMNFLGDWLRNHIKGSDMDYSPFL
---------W-----------D--HK-LV------------------------------L------HF--E--LF----------------------HK--H--FI-------------------------------------WLV-HI---D--Y---L
>Pelobacter propionicus (Deltaproteobacteria; Desulfuromonadales) 138aa YP_902721.1  GI:118581471
-----MGIRWRESLAI-GIEEIDSQHRELVEQFARLLAACE------QGRGEDELKSMLDFLDRYVRRHFSDEEILQQRYR---Y----------PAYQGHRNEHQNFIARIGSLQQQIAAR---------GVEVAHLVETNQLLYAWFVKHISSADRVLGSFL
>Pelobacter propionicus (Deltaproteobacteria; Desulfuromonadales) 143aa YP_901724.1  GI:118580474 FUGUE Z=22.4
----MSLMEWDGTYSI-GIKELDDHHQHLFMLINRAFDGYN------RGMGDAAVKKLLDELLEYATYHFSAEEMRMGEDA---Y----------PGIEEHYRQHKLFVNKVAELMELYKAV----------GSSILTSTLMFMNTWLTRHILQHDREYARYC
>Pelobacter propionicus (Deltaproteobacteria; Desulfuromonadales) 518aa (380-512) YP_902711.1  GI:118581461
HFRLSNALEWDASFAT-GIGTFDDEHKTLFRMVNDLHDAMQ------QKRSKEAIGQILDGLADYTVNHFAGEERAFSRTG---Y----------PEEAQHRELHRKLVDQVVELQGRFRDG----------QALLTQDVITFLQNWLVNHIKGVDKKYGPHL
>Polaromonas naphthalenivorans (Betaproteobacteria; Burkholderiales; Comamonadaceae) 152aa (6-133) YP_982345.1  GI:121605016
PLDASGSLQWSDQFLL-GYTPIDEVHQEFVDLMGQMQRAED-----------AALPALLAQFSVHLQHHFEMENEWMVSTE---F----------PPRECHMDEHAAVMQSVEEVQALLAQG----------DVAICRDLVAQLAQWFPQHADQLDSALAHWM
>Polaromonas naphthalenivorans (Betaproteobacteria; Burkholderiales; Comamonadaceae) 155aa (3-YP_973791.1  GI:121583360
KSSAAVAYFWEDKLLI-GHEQMDDEHQAFAHIIQALLKAPD-----------NAIASCLADVIAHATQHFAQEDEWMCNLD---F----------PARQCHMDEHAAVLRSAAGVKLRVHAG----------DHQAARLFSQELAAWLPPHVQHLDSALAAWI
>Polaromonas naphthalenivorans (Betaproteobacteria; Burkholderiales; Comamonadaceae) 168aa (1-125) YP_981479.1  GI:121604150
----MSNLEWSDALAL-NLPVMDDTHREFVELLAVVETASD-----------ETLLSHWRTLVDHTDDHFGREDQWMQATR---F----------SSTNCHSVQHKVVLQVMREGITHALAG----------DLGAIRQMARELAIWFPQHAQTMDAALALHL
>Pseudomonas aeruginosa (Gammaproteobacteria; Pseudomonadales) 153aa NP_250364.1  GI:15596870 YP_791573.1  GI:116049622
----MAHLVWQDDLNT-GIQVIDNQHKRIVEMINHLHDAQQ-------GKEHAAIAEVIEELVDYTLSHFAFEETLMEDAG---Y----------QFSRAHKKIHELFIRRVSEYRVRFQAG-----------EDVGDELKGLLSRWLFNHIRNDDAGYVDAV
>Pseudomonas resinovorans (Gammaproteobacteria; Pseudomonadales) 146aa (1-127) NP_758575.1  GI:27228525
-MDEKTEFAWDDSYLL-GHHAMDMVHREFVDLLQQAALTAPN-----------AELPNVLADIARHAEGHFAQENEWMESQG---F----------PGGDCHIDEHNKVLASIYDVQEKVIQG----------DIAIGRKLAKALSEWFPDHASFMDSALAAWL
>Pseudomonas resinovorans (Gammaproteobacteria; Pseudomonadales) 165aa BAB32774.1  GI:13094185
-MDEKTEFAWDDSYLL-GHHAMDMVHREFVDLLQQAALTAPN-----------AELPNVLADIARHAEGHFAQENEWMESQG---F----------PGGDCHIDEHNKVLASIYDVQEKVIQG----------DIAIGRKPGESPVRVVPRPCVFYGFCSRSVA
>Pseudomonas syringae pv. syringae (Gammaproteobacteria; Pseudomonadales) 160aa (1-133) YP_235578.1  GI:66045737
-MLPAPGSRPGDMTLNIFEALRES-HDRQRSYADALIQTSG---------DSAEREKAYKQLKEELQAHETAEERFFYIP----L---MAHDNGVDLSRHAISEHHEMDEMMEELDETEMSS-------------PAWLATAKKLSEKVHHHLKEEEQKFFQMA
---------W-----------D--HK-LV------------------------------L------HF--E--LF----------------------HK--H--FI-------------------------------------WLV-HI---D--Y---L
>Ralstonia eutropha (Betaproteobacteria; Burkholderiales) 146aa (1-131) YP_727244.1  GI:113868755
---------MDAKDQLSSAGLPDHLHLGEPVTDATHAEFVQ-LLAAVAGADDAGFLAAMDEWIDHTRHHFAQEEQWMEAMG---F----------GPRHCHAGEHQQVLAVAAAVRDKVHTD----------EFQLGRRLVAELSGWFDHHVKSMDAMMVEHM
>Ralstonia eutropha (Betaproteobacteria; Burkholderiales) 157aa (24-140) YP_295043.1  GI:73540523
MPPAAPAPSMDSKSQLSSDGLPADLHLGEPVTDATHAEFVQ-LLAAVAGADDAGFLAAMDEWIDHTRHHFAQEESWMEAMG---F----------GPRHCHAGEHQQVLAVAKAVRDKVASD----------GELELGRRLAAELSGWFDHHVKSMDAMMVEHM
>Ralstonia metallidurans (Betaproteobacteria; Burkholderiales) 160aa (9-147) YP_584783.1  GI:94311573
PYAGLSAEGLPDDLRL-GEPVTDATHAEFLQLLAAMEKADD-----------AGFLAAFEEWIEHTRHHFEQEEQWMEAMQ---F----------GPRFCHSNEHQGVMSVAKAVRDKIVEE-RADPEGPGKGALSLGRRLTGELSGWFDNHVKSMDTMMVEHM
>Ralstonia pickettii (Betaproteobacteria; Burkholderiales) 138aa (1-124) ZP_01663990.1  GI:121531385
----MPVIEWSEALQL-GDAATDANHVEFCALLNAVADATD-----------AEFIGALDAFIDHTEHHFAEENAWMDAAD---F----------PPRHCHRGEHDNVLALCREVHKRAAAG----------DMDLGRRLVAELPAWFAQHVDVMDRMMTTYL
>Ralstonia pickettii (Betaproteobacteria; Burkholderiales) 158aa (1-131) ZP_01661972.1  GI:121529360
----MNKAEIPVAQRAVLSMLLD-DHREVKKLFKTFQQEKD---------EATREQIVREACEKLTVHTQLEEEVFYP-----YVRDNGGEAFKDLVDEAVVEHATAKDLIQQLNGMHAGD-------------DLFAAKFTVLAEYVSHHVEEEETELFPKL
>Ralstonia solanacearum (Betaproteobacteria; Burkholderiales) 137aa NP_518898.1  GI:17545496
----MPVIQWSEALHL-GDAATDANHAAFCTLLNAVADASE-----------ADFVSALDAFIAHTEVHFAEENAWMEAAD---F----------PPLHCHRNEHDNVLALCREVRRRAADG----------DMALGRRLVTELPEWFAQHVDVMDRMMTTWL
>Reinekea sp. MED297 (Gammaproteobacteria; Reinekea) 150aa (1-130) ZP_01113653.1  GI:88798066
-----MTIKWTSALEI-GIPVIDAQHRITVEYINQVEHVQK------THSREELLDVLDELVDYTISHFAFEESLMEEAG---Y----------PAANAHSKVHRLFAKRVEAFQKRARAG-----------EDITEELLHVLKAWLVNHIQRDDQDYSADV
>Reinekea sp. MED297 (Gammaproteobacteria; Reinekea) 151aa ZP_01113654.1  GI:88798067
-----MKMQWTDEYEI-GIGVIDGQHQRIVEYINRIDDFEQ-------SGDQEAMNDVLASLVDYTLSHFAFEEALMEDVG---Y----------QELAEHKLTHDSFRRQIERLKARHDQG-----------EKVAVELSEMLLTWLLKHIMTDDQSYAPQV
>Rhodoferax ferrireducens (Betaproteobacteria; Burkholderiales) 277aa (146-273) YP_522715.1  GI:89900244
IHHFSTRIEWRDQYKI-GDQAIDEQHRELFKRAAYVLAATS----------HEGQVISAMRLLQYMRTHLSHEEEMMRRLH---Y----------PKLKVHTQEHLEMLARLNIISLRIAN-----------ENLVKSDLEELLGDWFLTHMETADTELARFA
>Rhodoferax ferrireducens (Betaproteobacteria; Burkholderiales) 498aa (364-496) YP_525259.1  GI:89902788
VAVNLVQLVWHPAYES-GQAELDAQHRALFGHVNNILAALL------LERPVDEVAALIDTLIGDVAKHFEDEEKIILAAG---F----------PGAAAHAAMHRELIAHAGYLVSRFKT-----------RDLDRGEPFQFLAHDLVARHILGEDREFFPHL
---------W-----------D--HK-LV------------------------------L------HF--E--LF----------------------HK--H--FI-------------------------------------WLV-HI---D--Y---L
>Rhodoferax ferrireducens (Betaproteobacteria; Burkholderiales) 840aa (688-822) YP_524860.1  GI:89902389
APKSHVHLRWKAAFAS-GNPVIDQEHETLFRLANTLLDEAA-----LRRQQPAAFEAAFEALLAHVVEHFAHEEAILLAHG---F----------AGMADHARQHQVLLARAHALHLSALA--------ADAGDATEGELVKFLVTELVAGHMLHTDSAFFPLF
>Rhodopseudomonas palustris BisA53 (Alphaproteobacteria; Rhizobiales) 504aa (358-480) NP_946988.1  GI:39934712
PGERRPDILWHETYAC-GEPTIDAQHRELFDLANVILGPEI------EMLEPARLSELVELLLTRVAEHFQYEQAVLEASG---Y----------DECGLHRMEHDHLLIRARQVSEAVVS-----------GHQSLGELRAFLVNSVILGHMLKDDRRFFPLF
>Rhodopseudomonas palustris BisA53 (Alphaproteobacteria; Rhizobiales) 507aa (354-487) YP_780986.1  GI:115524075
VDRSEPGLHWSRAYES-GDALIDSQHRELFVLANALFDPSL-----KPEAAPRELTLALDRLLSHVAKHFADEEAELSARH---F----------RGLSGHQAAHATLLRDADDLKNEVRS-----------GLRTLGDLREFIVNEVVAQHMLTADLEFFPLF
>Rhodopseudomonas palustris BisB18 (Alphaproteobacteria; Rhizobiales) 470aa (1-131) YP_532029.1  GI:90423659
----MVPFQWNALFET-GNARIDRQHRTLFELTNDLARAVQ------NGEKLPEISALVLKLRDYAATHFCDEEQLMRCSALA-I---------QEKTRHACAHRSFIKKVEDLAARRDL-----------SNVEAVSEFLQFLVTWLVTHILKLDHRMTKAL
>Rhodospirillum rubrum (Alphaproteobacteria; Rhodospirillales) 148aa (1-130) YP_426742.1  GI:83592990
----MVEITWSEKMSV-GNSALDWDHKTLLDLLNRFHGVAA------GQVNPSEVPALFTELVRYTEYHFQIEERLMRLAG---Y----------AELDKHEMHAALVSRLLAFQSQYLE----------APERFNFTAMADFLSDWLVRHILREDMKMGEVL
---------W-----------D--HK-LV------------------------------L------HF--E--LF----------------------HK--H--FI-------------------------------------WLV-HI---D--Y---L
>Rhodospirillum rubrum (Alphaproteobacteria; Rhodospirillales) 176aa (23-157) YP_428293.1  GI:83594541
GAAPWRPLIWLDVYSV-GDPLLDDDHRRLMEEINHLGAALI----NQIAPVSEALIAPLKRLAHQEAEHNLREEAILAQLG---Y----------PGLEDHRAEHRQLESGLGALVESLIP-----------QGPIEPEILADLLKDWFVRHVLGQDMRYKTYV
>Rhodospirillum rubrum (Alphaproteobacteria; Rhodospirillales) 209aa (1-130) YP_427169.1  GI:83593417
----MAPHSWSNRYAT-GLRAIDDDHRGLFEVLVDALEEHHE------KASGSSLVAATIAALQVYAAEHFAREERFMRKAH---F----------PDFERHHRIHEDFRALVDALARFQGQ----------SPDSVDLGRVTRFLNRWIGEHILVKDMEYLPYV
>Rhodospirillum rubrum (Alphaproteobacteria; Rhodospirillales) 262aa (133-260)YP_426610.1  GI:83592858
DLPHPAPRPWDRSLDT-GNARIDSHHRALSRLATRFHRLDG-------RADTGHLANLLHGLNTLMRLHFRTEETLWP-----------------ESDPHHALAHSHFLETLDHAGRRLLE-----------GEMSPSAFARDVLDHWLVDHIRAADQPRFRVL
>Rhodospirillum rubrum (Alphaproteobacteria; Rhodospirillales) 316aa (9-126) YP_427592.1  GI:83593840
GQGDLSLFNWSEERRL-GVRLMDRDHQVVCHQAGLLAAGVD-------RGDGAAMAAGARFLQDYIPRMFEREDRLMANT-------------NHPERGAHQDAHRQLLVGLMTRTSGPIE---------------TAASVLAAVETWIRGHIDAHDRPLARHV
---------W-----------D--HK-LV------------------------------L------HF--E--LF----------------------HK--H--FI-------------------------------------WLV-HI---D--Y---L
>Rhodospirillum rubrum (Alphaproteobacteria; Rhodospirillales) 736aa (591-723) YP_425723.1  GI:83591971
DGEDHALVTWSDDLAT-GDAHVDEDHRKLMALVNSVYGHVK------QGEAGAALEASFGELKRYSIEHFAEEEAFMATVN---F----------PQAETHRRQHQHFLGRSDALFEAYRR-----------GADTAAVELMGLLGSWWETHIRIYDTELAHFV
>Shewanella sp. ANA-3 (Gammaproteobacteria; Alteromonadales) 529aa (392-523) YP_868383.1  GI:117919191
QAQSKQFVKWGPELDI-GMPEINRQHQRLVSLINELHRTLS------EAYGLEAIKRIVQGLVDYTANHFSYEEELFARFG---Y----------PQTVAHKEKHAQLVGQVLDFQKRVAR-----------GEDVADELMAFLKAWLVNHIQKSDKEYTQFL
>Shewanella sp. MR-4 (Gammaproteobacteria; Alteromonadales) 529aa (392-523) YP_735348.1  GI:113971555
EAQSKLFVKWGPELDI-GMPEINRQHQRLVSLINELHRTLS------EAYGLEAIKRIVQGLVDYTANHFSYEEELFARFG---Y----------PQTVAHKEKHAQLVDQVLDFQKRVGR-----------GEDVADELMAFLKAWLVNHIQKSDKEYTQFL
>Shewanella sp. MR-7 (Gammaproteobacteria; Alteromonadales) 529aa (392-523) YP_735348.1  GI:113971555
EAQSKLFVKWGPELDI-GMPEINRQHQRLVSLINELHRTLS------EAYGLEAIKRIVQGLVDYTANHFSYEEELFARFG---Y----------PQTVAHKEKHAQLVSQVLDFQKRVAR-----------GEDVADELMAFLKAWLVNHIQKSDKEYTQFL
>Shewanella sp. PV-4 (Gammaproteobacteria; Alteromonadales) 530aa (393-518) ZP_00835633.1  GI:78365347
DTRRDKLIEWSDALDL-NLAEINRQHKTLVHLINELYYLLN------NNYGLASIKRVVQGLIDYTANHFKYEETLFEQFG---Y----------PQDKQHTDSHHQLVGKVLAFQRRVEQ-----------GEDIGDELMAFLKSWLSQHIQKEDKAYVDCF
>Shewanella sp. W3-18-1 (Gammaproteobacteria; Alteromonadales) 526aa (389-520) YP_964765.1  GI:120600191
QALSKLFVTWGPELDI-GMPEINRQHQRLVSLINELHRTLS------EDYGLEAIKRIVQGLVDYTANHFSYEEELFERFG---Y----------PQSESHKEKHGQLVGQVLDFQTRVGK-----------GEDVADELMSFLRSWLVNHIQKSDKEYTTFL
>Shewanella amazonensis (Gammaproteobacteria; Alteromonadales) 518aa (381-512) YP_926430.1  GI:119773690
IRHENAFVSWGPSLDI-GMAEVNRQHQRLITLINELHRAIN------EGYGLAAIKRIVQGLVDYTANHFSYEEELFARFD---Y----------PQSQDHEDEHKKLVTQVLDFQRRVGR-----------GENVSDELMAFLKAWLINHIQGSDKQYAPYL
>Shewanella baltica (Gammaproteobacteria; Alteromonadales) 529aa (392- ZP_00582180.1  GI:68542450
QAQNKLFVKWGPELDI-GMPEINRQHQRLVSLINELHRTLN------EEYGLEAIKRIVQGLVDYTANHFAYEEELFARFG---Y----------PQTDSHKEKHGKLVGQVLDFQKRVVK-----------GEDVADELMTFLKAWLVNHIQKSDKEYTAFL
>Shewanella oneidensis (Gammaproteobacteria; Alteromonadales) 529aa(392- NP_719421.1  GI:24375378
QAQNKLFVKWGPELDI-GMSEINRQHQRLVSVLNELHRTLS------EAYGLEAIKRIVQGLVDYTANHFSYEEELFARFG---Y----------PQTNGHKEKHAQLVGQVLDFQKRVAR-----------GEDVADELMAFLKSWLVNHIQKSDKEYAQFL
---------W-----------D--HK-LV------------------------------L------HF--E--LF----------------------HK--H--FI-------------------------------------WLV-HI---D--Y---L
>Shewanella putrefaciens (Gammaproteobacteria; Alteromonadales) 526aa (389-520) ZP_00815671.1  GI:77816487
QALSKLFVTWGPELDI-GMPEINRQHQRLVSLINELHRTLS------EDYGLEAIKRIVQGLVDYTANHFSYEEELFERFG---Y----------PQSESHKEKHGQLVGQVLDFQKRVGK-----------GEDVADELMSFLRSWLVNHIQKSDKEYTTFL
>Shewanella woodyi (Gammaproteobacteria; Alteromonadales) 159aa (1-132) ZP_01539832.1  GI:118071638
----MDPFQWDKNFAT-GIDNVDFQHKKLVDLINRFGHQLA-----CDELIMEDINQVLQELWLYTQEHFQDEERLMHEKG---I---------DPRHLTMQKQAHRYFLHEVQLMQASISI----------DQPEVLKYLLDFLTQWLAYHILGDDKNMARQI
>Solibacter usitatus (Acidobacteria; Solibacteres) 133aa YP_827140.1  GI:116624984
------MFDWKPQFAV-GIPSIDAQHQKLFAIARELYEAMS------AGRGKASLALILDRLVRYTATHFAHEERLMRI-----Y---------EYPDFVRHKAQHDALTKQVLGFQKEFDA-----------GMVSMTVQLLQFLKNWLENHIQVSDFEYGPCL
>Solibacter usitatus (Acidobacteria; Solibacteres) 141aa YP_823672.1  GI:116621516
----MSLFKWSKGHSV-YLPEIDAEHRNLCQMAAELHKIAL------AGGKSEQLKPILLNLLEAADEHFRHEERLMKVVH---Y----------SAFEWHKRQHDTVRKRLRQTIKRVEA-----------GETEACTDFLKFLGTWLVDHMTVTDRMMGAFI
>Solibacter usitatus (Acidobacteria; Solibacteres) 166aa (1-130) YP_826389.1  GI:116624233
---MTSSLVWSASTAV-FVTEIDDEHREIFDAISQVREALE------SRGSMPAIHKLAERLMGCLAEHFAHEERLMRAAR---Y----------GSTHWHQRSHRAARRRVEQFVGRLKE-----------GDTAAGHELVEYMTCWLTDHASVADRMMGSAL
>Sphingomonas sp. SKA58 (Alphaproteobacteria; Sphingomonadales) 154aa (1-123) ZP_01303096.1  GI:94496520
--------MADATIFHRLKQDHD-SHRQLFKKMDEAQGDDD------------RLKKLFDQFKVEATAHAAAEEETLYATM---L-----ARPDLREDAQHSVSEHKEIDDYLEEMEQDF------------NGKEWRETFAQLKKRYLHHIDEEEEEMFPDA
>Sphingomonas wittichii (Alphaproteobacteria; Sphingomonadales) 183aa (25-159)  ZP_01607194.1  GI:118759500
NAVRKIFVQAPTMMAGEWDDALAAEHRAVLELFDQIQATEE--------GHATRRSLLLTQIGHALAKHAMEEENAVYATLR--I------HGQVADADRLNGDHGYVKQYLFELGQMAPE-------------HPLFLSKIGDFRSMLEEHMRSEEETVFPKL
>Stenotrophomonas maltophilia (Gammaproteobacteria; Xanthomonadales) 153aa (1-127) ZP_01642673.1  GI:119875611
----MALLVWQDDLNI-GIDVIDQHRIIEMLNHLHVAQT-------SMQRAAVGEVIDEVVDYTMSHFAFEEELMEEAG---Y----------PFCAAHKRVHEVFIKRVAEYRLRFQA-----------EDISDELRTMLSRWLFNHIRGDDQAYADQV
>Symbiobacterium thermophilum (Actinobacteria; Symbiobacterium) 147aa (9-145) YP_075222.1  GI:51892531
AGGKEMALRWYSALETG-IEEIDGQHRQLFEQIDRLLESAK------SGAGQDACLRILDFLGGYVVEHFATEERYMERYG---Y----------PKKAEHKQHAELVEYYRKMRARVEA---------EGAKPTLIIQLQHSLVEWLNNHILKSDRDLGDFL
>Syntrophomonas wolfei (Firmicutes; Clostridia) 130aa YP_754830.1  GI:114567676
------MIRWKDEYSVG-VEHIDEQHRKLFEIAGRAYDLLK---DELVVDKYDRIVAILGELKDYSVFHFNSEEEYMKSIG---Y----------RKFLSHKVIHDDFIEKINGIDLDKVD----------ENQDKYLLDILDFIVRWIDGHILGQDKQYMQS-
---------W-----------D--HK-LV------------------------------L------HF--E--LF----------------------HK--H--FI-------------------------------------WLV-HI---D--Y---L
>Thermoanaerobacter tengcongensis (Firmicutes; Clostridia) 133aa NP_621951.1  GI:20806780
------MIKWTESLSVG-NETIDSQHKELIQKINDVLEACN------RQHGKEKVVEVMQFLKDYTVKHFKDEENLMKKYQ---Y----------PMYEEHKIHEDLVKKVEDLDERIKR---------EGVSLSVVMTVNKTLVDWFVNHISKEDKKVGEYI
>Thermosynechococcus elongatus (Cyanobacteria; Chroococcales) 136aa NP_682783.1  GI:22299536
---MLQRFEWTDRLSTG-VPMVDIQHKELVTAINDLADAIE------QGRGASRIKQLIVFMKYYAEWHFENEESCANK----Y-------RCPLAEVNHR-AHQQFIQLFEELQQQYRH---------SQADESVAVMIHGKLADWLVNHIMKIDKEIGQHI
>Thermosynechococcus elongatus (Cyanobacteria; Chroococcales) 149aa NP_682162.1  GI:22298915
```

```
----MDGLAWQAAFVSG-LAEVDEEHQALLEMLQQLRSAIA------AGATFSQVKPQLLACARETERHLQHEETLMATAN-------------HPLYLSHRAAHQNLLRDLSGLLEEVHG-----------HPENLTPETIEAIGTLVVRHICEEDLPMLYLL
>Thiobacillus denitrificans (Betaproteobacteria; Hydrogenophilales) 195aa (10-146) YP_314491.1  GI:74316751
TTGRQTARRSVASLRESVLDMLKDDHKRVKKAFRDFEKIDP-------HEDPERAAELVEQTCSELEVHAQLEEEIFYPAVRENI---SEADLVTEAEVEHKSAKQLIADIRSLSPEDEKY-----------------AASFTVLGEYVKHHIREEEQEMFKQL
>Thiomicrospira denitrificans (Epsilonproteobacteria; Campylobacterales) 1004aa (1-125) YP_393480.1  GI:78777165
-MKTIDIFPWSNHFNTE-IKIIDEQHKKLVIILNRLATHIA------YSDTDDDLNDIFDELIDYTSYHFETEEAIWHK-----Y------LPNDELDASHKVVHKTFVDTVLRLKNEQNR----------ESLSELAQEALGFLASWLASHILETDRHMAYIV
>Treponema denticola (Spirochaetes; Spirochaetales) 137aa NP_971622.1  GI:42526524
---MDDFVSWDASYDLG-IEYVDKQHRRLVELINELYHACL----GEKGELEEKFKHVMKELVEYVMIHFKDEEKIMEEIN---Y----------PNIKEHKQHHENFVKKILQSVNDYRN-----------GKQFVPNTFVRFLRDWLFNHILISDKEWAKFY
>Treponema denticola (Spirochaetes; Spirochaetales) 143aa NP_971908 GI:42526810
-MSEIKGFEWEDSLSVG-YNTIDLHHKKLLTIMGQFKDLFDL----PQEEYKLKIGKILKNLSDYTYYHFEEEEKVMKEYG---Y----------PYLKEHAEIHKSFIKKIREAIVPLAS-----------GNIETGLQFYDFLGRWLVEHIAGADHDWAEYI
>Verminephrobacter eiseniae (Betaproteobacteria; Burkholderiales) 168aa (15-143) YP_997683.1  GI:121609876
PRRGHDGPHLDHDGWLLGYAPMDRLHEEFLALVHALAQAPE-----------HAVAAALDALALNARAHFGAEDQWMAASA---F----------PARACHIDEHAAVMRSIEGVQRRVAR-----------ADFGAARPLADALLQWFPGHADYLDSALAHWL
>Vibrio cholerae (Gammaproteobacteria; Vibrionales) 372aa (1-132) NP_230861.1  GI:15641229
----MQSFKWDQYFETG-LEEVDEQHQSLVNIVNRYSSLLA-----ENHVSLDEIRLALFELSRYSEYHFKEEEKLMREVG---I--------SALHLEEHIQVHRTFMSEVFSMQAFIHD-----------VDDRSAVQLLEFLIHWLAYHILGIDQNMARQV
>Vibrio parahaemolyticus (Gammaproteobacteria; Vibrionales) 381aa NP_797668.1  GI:28898063
----MNSFRWDIYFETG-IEDVDDQHQYLVEFINKYGKLLT-----RNTISIADIQSALFELSRYAEFYFKEEENLMREVG---I--------HHEHLQKHIQVHRTFMGDIVSMQSFINE-----------DNRQSAEQLLDFLIHWLAYHILGIDQNMAKQI
---------W------------D--HK-LV-------------------------------L------HF--E--LF-----------------------HK--H--FI--------------------------------------WLV-HI---D--Y---L
>Vibrio splendidus (Gammaproteobacteria; Vibrionales) 383aa (21-146) ZP_00990126.1  GI:84387104
PKGSMNLFNWDKNFETG-IGVVDEQHQYLVGFINHYGNLLS---------------ENTISIDEINEFHFKEEESLMRDCGV--Y---------ELHIEEHIKVHRVFMQDIYSMQAFILE-----------EDQVSARQLLDFLIHWLTYHILGIDQNMARQM
>Vibrio vulnificus (Gammaproteobacteria; Vibrionales) 371aa (1-132) NP_761216.1  GI:27365688
----MNSFKWDKNFETG-IADVDEQHQYLVGFINRYGELIS-----ENTISIQDIQMALFELSRYAEFHFKEEENLMRNAGV--Y---------HEHLQYHIKIHRAFMSDIMGMQSFISE-----------ENKKSAEQLLHFLIHWLAYHILGIDQNMARQV
>Wolinella succinogenes (Epsilonproteobacteria; Campylobacterales) 168aa NP_907673.1  GI:34557858
--MPERILQWKEEYSIG-EEEIDSQHKRLFEIAAGAFSIVS------PSERAAKVKETIRRLVEYTRFHFDEEERFMKEEL---Y----------PHLDTHKELHEEIIISMNAFMRRIPE----------MKIQELEKELAFSIQSWFIDHITQKDKQIQAWL
>Xanthomonas axonopodis (Gammaproteobacteria; Xanthomonadales) 153aa (1-127) NP_642798.1  GI:21243216
----MALLVWQDDLNTG-IEVIDHQHRRIVDMINQLHVAQT-------SLQQLAVAEVIDELIDYTLSHFAFEEELMQEAG---Y----------PFSQAHQRVHAVFGKRVGEYRLRFQA------------GEDVSDELRNMLSRWLFNHIRGDDQAYAPQV
>Xanthomonas campestris (Gammaproteobacteria; Xanthomonadales) 153aa (1-127) NP_637705.1  GI:21231788
----MALLIWQDDLNTG-IEVIDHQHRRIVEMINHLHVAQT-------SLQRLVVSDVIDELIDYTLSHFAFEEELMEEAG---Y----------PFSHAHQRVHAVFAKRVGDYRLRFQA------------GEDISDELRNMLSRWLFNHIRGDDQAYAPQV
---------W------------D--HK-LV-------------------------------L------HF--E--LF-----------------------HK--H--FI--------------------------------------WLV-HI---D--Y---L

>Dechloromonas aromatica (Betaproteobacteria; Rhodocyclales) 187aa (18-176) YP_286170.1  GI:71908583
SALSDITAHWLTTGALPPELVTGHKLIDFEHRFLVSAIANLRKVCIDHETFADCSGCGHESQLRCENQLIGLLGDIFSFILDHFKTEESIMRDSLMLMVDRDMCQAHMEDHAEIAAKVQEIVSSLDQLHVVSRIRELEKLLARWITNHIALHDLLLARWI
---------W-----------------D--HK-LV---------------------------------------L------HF--E--LF--------------HK--H--FI----------------------------WLV-HI---D--Y---L
>Magnetococcus sp. MC-1 (Proteobacteria; Magnetococcus) 174aa (14-149) YP_867131.1  GI:117926514
-------MSQPTGLVRTGIGPIDRDHLHLWDLFQTLLEKDLRED---------EALAVLKELLAYTRYHFGREERLMQEIG---L--------TGEPRQAHIHEHAIFVKRVENFLELLQNRAAPKTTGLQAMVEEIQKMHQLPTLPQLDPAKRVVAFLVDWILNHTSGMDVELANHT
---------W------------D--HK-LV-------------------------------L------HF--E--LF-----------------------HK--H--FI----------------------------------------------------WLV-HI---D--Y---L
```

Fig. 3. A Bayesian phylogenetic tree of 3 protozoan and 42 metazoan sequences. The Hr sequences are identified by the fist three letter of the genus name and the first three letters of the species name (see Fig. 2 in Supplemental Material). The cnidarian *N. vectensis* Hr is marked with a dot.

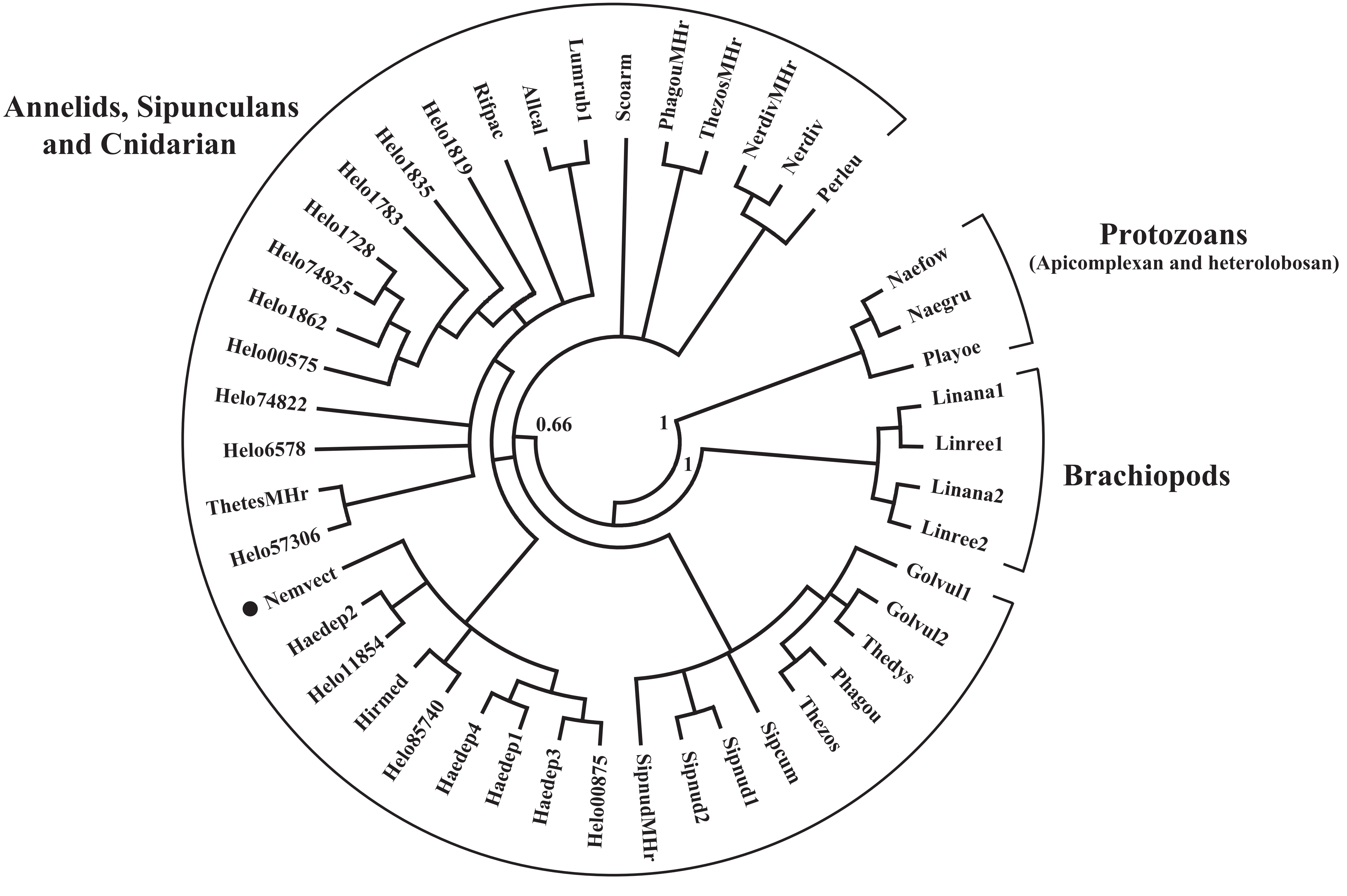

Annelids, Sipunculans and Cnidarian
Protozoans
(Apicomplexan and heterolobosan)
Brachiopods
Lumrub1
Scoarm
PhagouMHr
ThezosMHr
NerdivMHr
Nerdiv
Perleu
Allcal
Rifpac
Helo1819
Helo1835
Helo1783
Helo1728
Helo74825
Helo1862
Helo00575
Helo74822
Helo6578
ThetesMHr
Helo57306
Nemvect
Haedep2
Helo11854
Hirmed
Helo85740
Haedep4
Haedep1
Haedep3
Helo00875
SipnudMHr
Sipnud2
Sipnud1
Sipcum
Thezos
Phagou
Thedys
Golvul2
Golvul1
Linree2
Linana2
Linree1
Linana1
Playoe
Naegru
Naefow
0.66
1
1

Fig. 4. A Bayesian phylogenetic tree of 16 fungal, 3 protozoan and 42 metazoan sequences. The Hr sequences are identified by the fist three letter of the genus name and the first three letters of the species name (see Fig. 2 in Supplemental Material). The cnidarian *N. vectensis* Hr is marked with a dot.

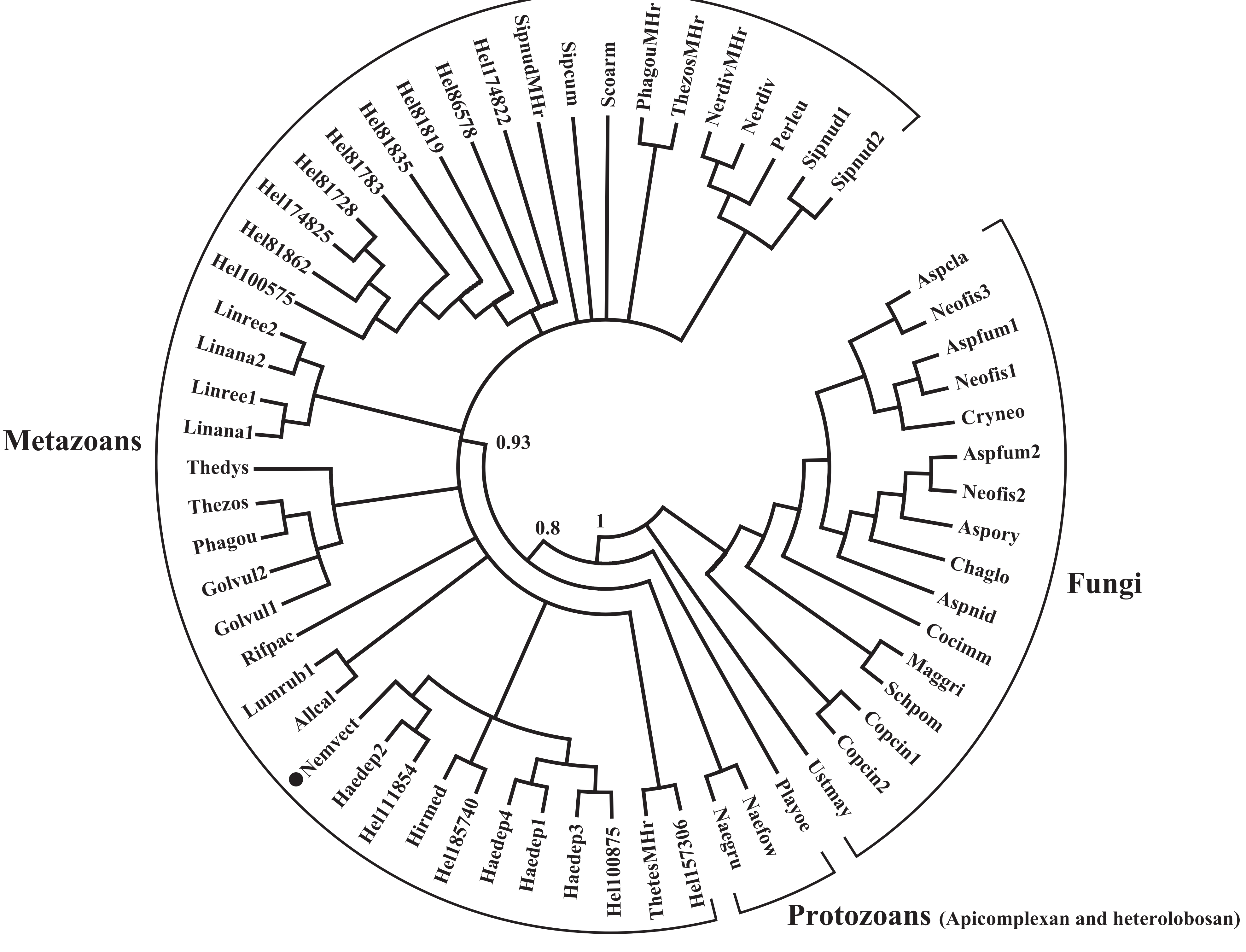
Metazoans
Fungi
Protozoans (Apicomplexan and heterolobosan)
0.93
0.8
1
Hel100575
Hel81862
Hel174825
Hel81728
Hel81783
Hel81835
Hel81819
Hel86578
Hel174822
SipnudMHr
Sipcum
Scoarm
PhagouMHr
ThezosMHr
NerdivMHr
Nerdiv
Perleu
Sipnud1
Sipnud2
Aspcla
Neofis3
Aspfum1
Neofis1
Cryneo
Aspfum2
Neofis2
Aspory
Chaglo
Aspnid
Cocimm
Maggri
Schpom
Copcin1
Copcin2
Ustmay
Playoe
Naefow
Naegru
Hel157306
ThetesMHr
Hel100875
Haedep3
Haedep1
Haedep4
Hel185740
Hirmed
Hel111854
Haedep2
Nemvect
Allcal
Lumrub1
Rifpac
Golvul1
Golvul2
Phagou
Thezos
Thedys
Linana1
Linree1
Linana2
Linree2

Table 1. Putative hemerythrins in eukaryote genomes.

Taxon	Identifier	Size and Hr domain (aa)
Alveolata		
Plasmodium yoelii yoelii	gb|AABL01004457.1	132
Annelida		
Hirudinae		
Haementeria depressa	gi|47833904|gb|CN807658.1	119
Helobdella robusta	jgi|Helro1|157306	120
	jgi|Helro1|100875	120
	jgi|Helro1|81783	121
	jgi|Helro1|185740	123
	jgi|Helro1|111854	132
	jgi|Helro1|100575	134
	jgi|Helro1|81819	134
	jgi|Helro1|174825	134
	jgi|Helro1|174822	134
	jgi|Helro1|81862	134
	jgi|Helro1|81835	136
	jgi|Helro1|175368	136
	jgi|Helro1|86578	165
Hirudo medicinalis	AAS49167.1 GI:44921619	120
Theromyzon tessulatum	Q9GYZ9 GI:21263690	120
Oligochaetea		
Allolobophora caliginosa	Ref 10	119
Polychaeta		
Neanthes (Nereis) diversicolor	P80255 GI:417311	119
	P22761 GI:123030	120
Periserrula leucophryna	AAQ63634.1 GI:34327785	119
Riftia pachyptila	This work	110?
Scoloplos armiger	This work	110?
Brachiopoda		
Lingula anatina	P22764 GI:123038	117
	P22765 GI:123044	117
Lingula reevii	P23543 GI:46397821	117
	P23544 GI:46397822	117
Cnidaria		
Nematostella vectensis	XP_001634535.1 GI:156388091	119
	XP_001622541.1 GI:156351502	156, 2-117
Fungi		
Ascomycota		
Ajellomyces capsulatus	XP_001539786.1 GI:154277908	205, 12-146
Aspergillus clavatus	XP_001271296.1 GI:121706066	220, 26-160
	XP_001270510.1 GI:121704493	220, 21-160
Aspergillus fumigatus	XP_754309.1 GI:70999175	221, 26-160
	XP_747053.1 GI:70983051	227, 31-160
Aspergillus nidulans	XP_681907.1 GI:67903302	228, 24-158

Aspergillus oryzae	BAE63202.1 GI:83773074	221, 33-157
Coccidioides immitis	XP_001245291.1 GI:119189369	181, 1-121
Magnaporthe grisea	XP_360008.1 GI:39940942	239, 34-169
	XP_001261781.1 GI:119483756	227, 31-160
Neosartorya fischeri	XP_001263153.1 GI:119490985	221, 25-160
	XP_001267572.1 GI:119501631	233, 25-160
Neurospora crassa	XP_955932.1 GI:85076485	232, 33-167
Schizosaccharomyces pombe	NP_595013.1 GI:19115925	203, 11-144
Basidiomycota		
Coprinopsis cinerea okayama	EAU86662.1 GI:116503767	208, 1-130
	EAU89470.1 GI:116506575	228, 17-153
Cryptococcus neoformans	XP_566714.1 GI:58258603	220, 15-159
Ustilago maydis (zeae)	XP_757573.1 GI:71005814	244, 41-170
Heterolobosa		
Naegleria fowleri	Q9NH76 GI:21263696	119
Naegleria gruberi	jgi|Naegr1|44819|fgeneshHS_pm.scaffold_69000005	119
	jgi|Naegr1|81770|estExt_fgeneshNG_pg.C_680095	119
	jgi|Naegr1|83311|estExt_fgeneshNG_pm.C_680002	119
	jgi|Naegr1|83016|estExt_fgeneshNG_pm.C_100003	119
	jgi|Naegr1|36233|estExt_gwp_gw1.C_160163	119
Sipuncula		
Golfingia vulgaris	CAG14945.1 GI:57282926	114
	CAG14946.1 GI:57282928	113
Phascolopsis gouldii	P02244 GI:21264441	114
	P27686 GI:232242	119
Siphonosoma cumanense	P22766 GI:123040	113
Sipunculus nudus	CAG14943.1 GI:57282922	119
	CAF74912.1 GI:57282893	119
	CAG14944.1 GI:57282924	119
Themiste dyscritum	P02246 GI:123041	113
Themiste zostericola	P02245 GI:123042	113
	P02247 GI:417114	118

Table 2. Putative hemerythrins in archaeal genomes

Taxon	Identifier	Size and Hr domain (aa)
Euryarcheota		
Halobacteriales		
Haloarcula marismortui	YP_134671.1 GI:55376820	617, 12-146
Methanococcales		
Methanocaldococcus jannaschii	Q58157 GI:21263658	147
	NP_247732.1 GI:15668928	169, 21-154
Methanomicrobiales		
Methanoregula boonei	gi|154149997|ref|YP_001403615.1	135
Methanospirillum hungatei	YP_504166.1 GI:88603988	135
	YP_502435.1 GI:88602257	135
No. genomes with Hr/Total	4/43	

Table 3. Identified and putative hemerythrins in bacteria[1].

Taxon	Identifier	Size and Hr domain	Oxygen requirement and habitat [2]
Acidobacteria			
Acidobacteria bacterium Ellin 345	YP_592725|GI:94970677<br>YP_593517|GI:94971469<br>YP_591227|GI:94969179<br>YP_592726|GI:94970678<br>YP_592724|GI:94970676	132, 1-128<br>134. 1-128<br>142, 1-131<br>152, 1-129<br>155, 1-129	Ae, Tr
Solibacter usitatus	YP_827140.1 GI:116624984<br>YP_823672.1 GI:116621516<br>YP_826389.1 GI:116624233	133, 1-127<br>141, 1-129<br>166, 1-130	
No. genomes with Hrs/Total	2/3		
Actinobacteridae (high G+C, Gram+)<br>*Symbiobacterium thermophilum*	YP_075222.1 GI:51892531	147, 9-143	MAe, Tr
No. genomes with Hrs/Total	1/39		
Aquificales			
Aquifex aeolicus	NP_214180.1 GI:15606800	131, 1-129	Ae, Sp
Hydrogenobacter thermophilus	BAF34936.1 GI:116234997	131, 1-129	
No. genomes with Hrs/Total	2/3		
Cyanobacteria (Blue green algae)			
Chrooccocales<br>*Thermosynechoccus elongates*	NP_682783.1 GI:22299536<br>NP_682162.1 GI:22298915	136, 1-132<br>149, 1-129	Sp
No. genomes with Hrs/Total	1/20		
Firmicutes (Low G + C, Gram +)			
Clostridia			

Alkaliphilus metalliredigenes	ZP_00800966.1 GI:77685572	137, 1-131	?
Caldicellulosiruptor saccharolyticus	ZP_00885869.1 GI:82500456	138, 1-131	An, Sp
Clostridium sp. OhILAs	ZP_01361119.1 GI:106893996<br>ZP_01360922.1 GI:106893798<br>ZP_01360653.1 GI:106893528	131, 1-127<br>137, 1-132<br>142, 1-132	An, Aq
Clostridium acetobutylicum	NP_346715.1 GI:15893366	129	An, Aq
Clostridium beijerincki	ZP_00910041.1 GI:82747545<br>ZP_00911352.1 GI:82748877<br>ZP_00910329.1 GI:82747836<br>ZP_00908279.1 GI:82745769<br>ZP_00909310.1 GI:82746807	129<br>129<br>130<br>133<br>135	An, M
Clostridium cellulolyticum	ZP_01574112.1 GI:118725472<br>ZP_01576604.1 GI:118728026<br>[ZP_00909308.1 GI:82746805	130<br>132<br>185, 44-179	
Clostridium phytofermentans	ZP_01353421.1 GI:106886073<br>ZP_01354048.1 GI:106886715	129<br>143, 1-135	An, M
Clostridium tetani	NP_782896.1 GI:28211952	137, 1-131	An, Aq
Clostridium thermocellum	ZP_00504934.1 GI:67875718	129	An, M
Desulfitobacterium hafniense	ZP_01371607.1 GI:109647704<br>YP_517407.1 GI:89893920<br>YP_516471.1 GI:89892984	135, 1-131<br>139, 1-132<br>144, 1-134	An, M
Syntrophomonas wolfei	YP_754830.1 GI:114567676	130	An, Aq
Ruminococcus gnavus	ref|NZ_AAYG01000020.1	120	An, HA
Ruminococcus torques	ref|NZ_AAVP01000029.1	120	An, HA
Thermoanaerobacter tengcongensis	NP_621951.1 GI:20806780	133	An, Aq
No. genomes with Hrs/Total	14/21		
No. genomes with Hrs/Total	14/75		
Planctomycetes			
Kuenenia stuttgartiensis	CAJ72144.1 GI:91202505<br>CAJ71928.1 GI:91204275	129<br>872, 734-866	An
No. genomes with Hrs/Total	1/4		

Proteobacteria (Purple bacteria)			
Alphaproteobacteria			
Rhizobiales			
Mesorhizobium sp. BNC1	YP_665917.1 GI:110347099	134, 1-129	Ae, M
Rhodopseudomonas palustris BisA53	NP_946988.1 GI:39934712<br>YP_780986.1 GI:115524075	504, 358-480<br>507, 354-487	F, M
Rhodopseudomonas palustris BisB18	YP_532029.1 GI:90423659	470, 1-124	F, M
No. genomes with Hrs/Total	3/25		
Rhodospirilalles			
Magnetospirillum gryphiwaldense	CAE12026.1 GI:33945221<br>CAJ30087.1 GI:78033471<br>CAJ30107.1 GI:78033490	149, 1-127<br>159, 1-136<br>524, 197-329	
Magnetospirillum magneticum	YP_419883.1 GI:83309619<br>YP_421315.1 GI:83311051<br>YP_419932.1 GI:83309668<br>YP_420778.1 GI:83310514<br>YP_420912.1 GI:83310648<br>YP_419589.1 GI:83309325<br>YP_423659.1 GI:83313395<br>YP_423534.1 GI:83313270<br>YP_420035.1 GI:83309771<br>YP_422017.1 GI:83311753<br>YP_423499.1 GI:83313235<br>YP_421602.1 GI:83311338<br>YP_421612.1 GI:83311348<br>YP_423628.1 GI:83313364<br>YP_420779.1 GI:83310515<br>YP_420100.1 GI:83309836<br>YP_422781.1 GI:83312517<br>YP_422454.1 GI:83312190<br>YP_423329.1 GI:83313065<br>YP_421350.1 GI:83311086<br>YP_423198.1 GI:83312934<br>YP_420298.1 GI:83310034<br>YP_423764.1 GI:83313500<br>YP_420741.1 GI:83310477<br>YP_420348.1 GI:83310084<br>YP_422163.1 GI:83311899<br>YP_422102.1 GI:83311838<br>YP_420086.1 GI:83309822<br>YP_421898.1 GI:83311634<br>YP_422973.1 GI:83312709	122<br>127<br>129<br>132<br>134<br>135<br>136<br>138<br>138<br>140<br>140<br>142<br>143<br>144<br>144<br>146<br>146, 1-139<br>147, 1-132<br>148, 7-148<br>149, 11-141<br>156, 8-142<br>157, 5-138<br>161, 1-132<br>169, 9-135<br>171, 18-148<br>171, 27-158<br>179, 40-160<br>275, 1-134<br>181, 1-129<br>499, 1-131	MAe, Aq

	YP_420234.1 GI:83309970<br>YP_422630.1 GI:83312366<br>YP_423519.1 GI:83313255<br>YP_419583.1 GI:83309319<br>YP_423156.1 GI:83312892<br>YP_422458.1 GI:83312194<br>YP_421045.1 GI:83310781	501, 19-150<br>665, 529-659<br>795, 658-788<br>807, 672-800<br>810, 673-803<br>870, 740-864<br>906, 774-900	
Magnetospirillum magnetotacticum	ZP_00052849.2 GI:46202650<br>ZP_00053268.2 GI:46202398<br>ZP_00054990.2 GI:46201470<br>ZP_00053732.1 GI:23013881<br>ZP_00055446.1 GI:23015677<br>ZP_00053735.1 GI:23013885<br>ZP_00054355.1 GI:23014545<br>ZP_00051444.1 GI:23010917<br>ZP_00207881.1 GI:46200880<br>ZP_00052929.2 GI:46202606<br>ZP_00055246.1 GI:23015471<br>ZP_00054325.1 GI:23014513<br>ZP_00052752.1 GI:23012741<br>ZP_00055707.1 GI:23015944<br>ZP_00055084.1 GI:23015304<br>ZP_00055499.1 GI:23015731<br>ZP_00055962.2 GI:46201047<br>ZP_00052625.1 GI:23012573`<br>ZP_00056082.1 GI:23016325<br>ZP_00053162.2 GI:46202468<br>YP_422102.1 GI:83311838<br>ZP_00053965.2 GI:46202007<br>ZP_00054151.2 GI:46201886<br>ZP_00054349.2 GI:46201789<br>ZP_00056481.1 GI:23016728<br>ZP_00056272.1 GI:23016517<br>ZP_00055928.1 GI:23016169<br>ZP_00054329.1 GI:23014517<br>ZP_00055643.1 GI:23015878	114<br>120<br>122<br>127<br>131<br>132<br>134<br>134<br>135<br>135<br>136<br>139<br>140<br>142, 1-133<br>144, 1-132<br>144, 1-129<br>146, 7-138<br>150, 1-131<br>158, 1-140<br>159, 1-138<br>179, 31-160<br>272, 1-131<br>274, 1-121<br>500, 1-130<br>699, 563-692<br>807, 668-800<br>808, 672-808<br>870, 736-864<br>889, 754-883	MAe, Aq
Rhodospirillum rubrum	YP_426742.1 GI:83592990<br>YP_428293.1 GI:83594541<br>YP_427169.1 GI:83593417<br>YP_426610.1 GI:83592858<br>YP_427592.1 GI:83593840<br>YP_425723.1 GI:83591971	148, 1-130<br>176, 23-157<br>209, 1-130<br>262, 133-262<br>316, 9-126<br>736, 590-732	F, M
No. genomes with Hrs/Total	4/6		
Sphingomonadales			
Sphingomonas sp. SKA58	ZP_01303096.1 GI:94496520	154, 1-122	Ae, Aq
Sphingomonas wittichii	ZP_01607194.1 GI:118759500	184, 25-159	Ae, Aq

No. genomes with Hrs/Total	2/6		
No. genomes with Hrs/Total	9/75		
Betaproteobacteria			
Burkholderiales			
Acidovorax sp. JS42	YP_984586.1 GI:121592690<br>YP_984955.1 GI:121593059	168, 1-124<br>173, 28-160	Ae, Tr
Acidovorx avenae	YP_968695.1 GI:120609017	171, 1-124	Ae, M
Bordetella avium	CAJ48206.1 GI:115421695	148, 1-124	Ae, HA
Burkholderia mallei	YP_102393.1 GI:53725253	149, 7-135	HA
Burkholderia pseudomalllei	YP_334215.1 GI:76810098	186, 44-172	An, Tr
Burkholderia phymatum	ZP_01500286.1 GI:118028824<br>ZP_01504458.1 GI:118033012	147, 1-127<br>190, 1-123	HA
Burkholderia thailandensis	YP_442321.1 GI:83719963	141, 1-127	Ae, Tr
Burkholderia vietnamiensis	ZP_00427022.1 GI:67549150	158, 13-142	F, M
Burkholderia xenovorans	YP_556187.1 GI:91780980<br>YP_555832.1 GI:91780625	159, 7-135<br>165, 12-140	Ae, M
Comamonas testoreni	ZP_01520527.1 GI:118051979<br>ZP_01518696.1 GI:118050146	165, 1-124<br>211, 37-160	Ae, M
Delftia acidovorans	ZP_01580041.1 GI:118731526<br>ZP_01578319.1 GI:118729802	166, 1-124<br>213, 40-170	Ae, M
Janthinobacterium sp. J3	BAC56750.1 GI:28201215	149, 1-127	?
Janthinobacterium lividum	ABK64032.1 GI:118161348	132	?
Polaromonas naphthalenivorans	YP_982345.1 GI:121605016<br>YP_973791.1 GI:121583360<br>YP_981479.1 GI:121604150	152, 6-133<br>155, 3-130<br>168, 1-125	Ae, Aq
Ralstonia eutropha	YP_727244.1 GI:113868755<br>YP_295043.1 GI:73540523	146, 1-131<br>157, 1-140	F, M
Ralstonia metallidurans	YP_584783.1 GI:94311573	160, 9-147	F, Sp
Ralstonia picketi	ZP_01663990.1 GI:121531385<br>ZP_01661972.1 GI:121529360	131<br>158, 1-130	

Ralstonia solanacearum	NP_518898.1 GI:17545496	137	Ae, M
Rhodoferax ferrireducens	YP_522715.1 GI:89900244<br>YP_525259.1 GI:89902788<br>YP_524860.1 GI:89902389	277, 138-272<br>498, 366-496<br>840, 691-822	F, M
Verminephrobacter eiseniae	YP_997683.1 GI:121609876	168, 22-143	
No. genomes with Hrs/Total	20/26		
Hydrogenophilales			
Thiobacillus denitrificans	YP_314491.1 GI:74316751	195, 10-146	F, M
Rhodocyclales			
Azoarcus sp.EbN1	YP_932039.1 GI:119896826<br>YP_935261.1 GI:119900048<br>YP_932615.1 GI:119897402<br>YP_932470.1 GI:119897257<br>YP_935260.1 GI:119900047	136<br>150, 1-132<br>160, 1-130<br>162, 1-122<br>174, 19-148	F, Tr
Dechloromonas aromatica	YP_284639.1 GI:71907052<br>YP_285883.1 GI:71908296<br>YP_284878.1 GI:71907291<br>YP_284887.1 GI:71907300<br>YP_284625.1 GI:71907038<br>YP_286895.1 GI:71909308<br>YP_285962.1 GI:71908375<br>YP_284835.1 GI:71907248<br>YP_284552.1 GI:71906965<br>YP_286170.1 GI:71908583<br>YP_287152.1 GI:71909565<br>YP_284191.1 GI:71906604	129<br>127<br>128<br>133<br>134<br>142, 1-130<br>146, 1-127<br>160, 1-126<br>161, 10-140<br>187, 24-176<br>667, 534-661<br>681, 1-129	F, M
No. genomes with Hrs/Total	24/33		
Gammaproteobacteria			
Aeromonadales			
Aeromonas hydrophila	YP_854665.1 GI:117619147<br>YP_857472.1 GI:117618414	678, 540-672<br>809, 680-807	F, M
Alteromonadales	9 other genomes - no Hr		
Colwellia psychoerythrae	YP_268476.1 GI:71281331<br>YP_270871.1 GI:71282489<br>YP_270886.1 GI:71279092<br>YP_268475.1 GI:71280761<br>YP_271339.1 GI:71280063<br>YP_270299.1 GI:71279208	131<br>188, 52-182<br>192, 52-182<br>368, 1-132<br>519, 389-509<br>722, 1-123	F, Sp

	YP_268669.1 GI:71280946	1328, 1180-1310	
Idiomarina baltica	ZP_01041958.1 GI:85710897	566, 427-560	Ae, Aq
Idiomarina loihiensis	YP_154567.1 GI:56459286	832, 603-826	Ae, Sp
Marinobacter aquaeole	YP_960352.1 GI:120556001	662, 8-136	F, Aq
Shewanella sp. ANA-3	YP_868383.1 GI:117919191	529, 390-524	F, M
Shewanella sp. MR-4	YP_735348.1 GI:113971555	529, 394-523	F, M
Shewanella sp. MR-7	YP_735348.1 GI:113971555	529, 394-523	F, Aq
Shewanella sp. PV-4	ZP_00835633.1 GI:78365347	528, 395-524	F, M
Shewanella sp. W3-18-1	YP_964765.1 GI:120600191	526, 387-520	F, M
Shewanellla amazonensis	YP_926430.1 GI:119773690	518, 383-513	F, M
Shewanella baltica	ZP_00582180.1 GI:68542450	529, 394-523	F, Aq
Shewanella oneidensis	NP_719421.1 GI:24375378	529, 394-523	F, M
Shewanella putrefaciens	ZP_00815671.1 GI:77816487	526, 391-520	F, M
Shewanella woodyi	ZP_01539832.1 GI:118071638	159, 1-131	F, M
No. genomes with Hrs/Total	14/23		
Chromatiales			
Alkalilimnicola ehrlichei	YP_741251.1 GI:114319568	161, 1-127	F, Aq
Methylococcales			
Methylococcus capsulatus	YP_113225.1 GI:53805087	131	Ae, M
Oceanospirillales	4 other genomes - no Hrs		
Alkanivorax borkumensis	YP_691784.1 GI:110832925	154, 1-127	Ae, Aq

Oceanospirillum sp. MED92	ZP_01167458.1 GI:89094519<br>ZP_01165824.1 GI:89092872<br>ZP_01166597.1 GI:89093650	121<br>129<br>135	Ae, Aq
Pseudomonadales			
Acinetobacter baumannii	ref|NC_009085.1		Ae, M
Azotobacter vinelandii	ZP_00417575.1 GI:67155948	134	Ae, M
Pseudomonas aeruginosa	NP_250364.1 GI:15596870	153, 1-127	Ae, M
Pseudomonas mendocina	ZP_01526847.1 GI:118058386	155, 1-125	
Pseudomonas resinovorans	NP_758575.1 GI:27228525<br>BAB32774.1 GI:13094185	146, 1-127<br>165, 1-127	
Pseudomonas syringae	YP_235578.1 GI:66045737	160, 1-133	Ae, M
No. genomes with Hrs/Total	6/14		
Reinekea			
Reinekea sp. MED29	ZP_01113653.1 GI:88798066<br>ZP_01113654.1 GI:88798067	150, 1-126<br>151, 1-126	Aq
Vibrionales			
Vibrio cholerae	NP_230861.1 GI:15641229	372, 1-132	F, Aq
Vibrio parahaemolyticus	NP_797668.1 GI:28898063	381, 1-132	F, Aq
Vibrio splendidus	ZP_00990126.1 GI:84387104	383, 14-146	F, Aq
Vibrio vulnificus	NP_761216.1 GI:27365688	371, 1-132	F, Aq
No. genomes with Hrs/Total	4/11		
Xanthomonadales			
Stenotrophomonas maltophilia	ZP_01642673.1 GI:119875611	153, 1-127	
Xanthomonas axonopodis	NP_642798.1 GI:21243216	153, 1-127	Ae, HA
Xanthomonas campestri	NP_637705.1 GI:21231788	153, 1-127	Ae, HA

No. genomes with Hrs/Total	3/5		
Unclassified gammaproteobacteria			
Marine gamma proteobacterium	ZP_01224113.1 GI:90416181	174, 29-166	Aq
No. genomes with Hrs/Total	34/104		
Deltaproteobacteria			
Bdellovibrionale			
Bdellovibrio bacteriovorus	NP_970264.1 GI:42524884	136	Ae, M
Desulfobacterales			
Desulfotalea psychrophila	YP_065523.1 GI:51245639<br>YP_064708.1 GI:51244824<br>YP_065775.1 GI:51245891	156, 1-129<br>370,1-132<br>374, 1-130	An, Sp
Desulfovibrionales	One other genome - no Hrs		
Desulfovibrio desulfuricans	YP_386749.1 GI:78355300<br>YP_386930.1 GI:78355481<br>YP_389304.1 GI:78357855	150, 1-143<br>579, 441-573<br>722, 580-722	An, M
Desulfovibrio vulgaris	YP_389304.1 GI:78357855<br>YP_012317.1 GI:46581509<br>YP_009395.1 GI:46578587<br>AAB50497.1 GI:887858<br>YP_012365.1 GI:46581557	137<br>655, 520-646<br>689, 548-682<br>959, 810-955<br>963, 827-957	An, M
Desulfuromonadales			
Desulforomonas acetoxidans	ZP_01311722.1 GI:95928977<br>ZP_01311940.1 GI:95929196<br>ZP_01313281.1 GI:95930546<br>ZP_01313282.1 GI:95930547<br>ZP_01313679.1 GI:95930950<br>ZP_01312822.1 GI:95930083<br>ZP_01313525.1 GI:95930793<br>ZP_01313825.1 GI:95931099	132<br>133<br>142, 1-129<br>199, 35-159<br>368, 1-131<br>371, 7-132<br>591, 8-132<br>1174, 1030-1174	An
Geobacter sp. FRC-32	ZP_01390530.1 GI:110602439<br>ZP_01387533.1 GI:110599293	132<br>135	An, M
Geobacter lovleyi	ZP_01593656.1 GI:118745688<br>ZP_01594022.1 GI:118746066<br>ZP_01592161.1 GI:118744164<br>ZP_01593682.1 GI:118745715<br>ZP_01594207.1 GI:118746259	135<br>137<br>147, 1-131<br>518, 372-512<br>768, 620-740	

Geobacter metallireducens	YP_386060.1 GI:78224313<br>YP_383801.1 GI:78222054	135<br>155, 12-146	An, Aq
Geobacter sulfurreducens	NP_953971.1 GI:39998020<br>NP_952095.1 GI:39996144<br>NP_951461.1 GI:39995510<br>NP_953680.1 GI:39997729<br>NP_951317.1 GI:39995366	131<br>134<br>135<br>139<br>145	An, M
Geobacter uraniumreducens	ZP_01143645.1 GI:88938143<br>ZP_01140038.1 GI:88934395	135<br>138	MAe, M
Pelobacter carbinolicus	YP_355938.1 GI:77918123	135	An, Aq
Pelobacter propionicus	YP_902721.1 GI:118581471<br>YP_901724.1 GI:118580474<br>YP_902711.1 GI:118581461	138<br>143<br>518, 380-508	An, M
No. genomes with Hrs/Total	7/7		
Myxococcales			
Anaeromyxobacter SP.Fw109-5	ZP_01667972.1 GI:121536194<br>ZP_01668151.1 GI:121536373<br>ZP_01669396.1 GI:121537620<br>ZP_01670274.1 GI:121538500<br>ZP_01669455.1 GI:121537679<br>ZP_01671347.1 GI:121539576	132<br>133<br>135<br>142, 1-135<br>149, 1-129<br>195, 44-177	
Anaeromyxobacter dehalogenans	YP_467301.1 GI:86160516<br>YP_466223.1 GI:86159438<br>YP_466224.1 GI:86159439<br>YP_463433.1 GI:86156648<br>YP_463788.1 GI:86157003<br>YP_463705.1 GI:86156920<br>YP_463306.1 GI:86156521<br>YP_463596.1 GI:86156811	133<br>133<br>133<br>138<br>144, 1-129<br>144, 1-129<br>146, 1-130<br>148, 1-135	An, Tr
Myxococcus xanthus	YP_635509.1 GI:108758665	185, 11-139	
Unclassified deltaproteobacteria			
Deltaproteobacterium MLMS-1	ZP_01288141.1 GI:94264349<br>ZP_01288112.1 GI:94264319<br>ZP_01291329.1 GI:94268940<br>ZP_01290755.1 GI:94267120	135<br>148, 10-142<br>296, 150-283<br>457, 320-450	Aq
No. genomes with Hrs/Total	14/18		

Epsilonproteobacteria			
Campylobacter coli	ZP_00367964.1 GI:57168833<br>ZP_00367250.1 GI:57168111<br>ZP_00367884.1 GI:57168753<br>ZP_00367885.1 GI:57168754<br>ZP_00368169.1 GI:57169041	112<br>199. 1-123<br>203, 1-128<br>229, 1-130<br>268, 1-134	Mae, M
Campylobacter fetus	YP_892819.1 GI:118474426<br>YP_891782.1 GI:118474590	183, 1-127<br>385, 1-143	MAe, M
Campylobacter jejuni	YP_999771.1 GI:121613643<br>ZP_01068960.1 GI:86150744<br>ZP_01069655.1 GI:86151450<br>ZP_01072240.1 GI:86154039<br>NP_281267.1 GI:15791444<br>ZP_01067851.1 GI:86149621<br>YP_178071.1 GI:57237059	144, 1-132<br>199, 1-127<br>202, 1-132<br>239. 1-132<br>240, 1-132<br>242, 1-132<br>254, 1-132	MAe, M
Campylobacter lari	ZP_00368466.1 GI:57240517	187, 1-132	MAe, M
Campylobacter upsaliensis	ZP_00370672.1 GI:57242735<br>ZP_00369894.1 GI:57241954<br>ZP_00371184.1 GI:57505255	199, 1-127<br>202, 1-127<br>233, 1-127	MAe, M
Helicobacter hepaticus	NP_859535.1 GI:32265503<br>NP_859536.1 GI:32265504	184, 1-128<br>187, 1-128	
Thiomicrospira denitrificans	YP_393480.1 GI:78777165	1004, 1-128	An, Aq
Wolinella succinogenes	NP_907673.1 GI:34557858	168, 1-132	MAe, HA
No. genomes with Hrs/Total	8/13		
Unclassified proteobacteria			
Magnetococcus sp. MC-1	YP_866138.1 GI:117925521<br>YP_867250.1 GI:117926633<br>YP_867131.1 GI:117926514<br>YP_866733.1 GI:117926116<br>YP_867005.1 GI:117926388<br>YP_864332.1 GI:117923715<br>YP_864533.1 GI:117923916<br>YP_866360.1 GI:117925743<br>YP_867064.1 GI:117926447<br>YP_867464.1 GI:117926847<br>YP_867464.1 GI:117926847<br>YP_864243.1 GI:117923626<br>YP_865500.1 GI:117924883<br>YP_864127.1 GI:11792351	134<br>160, 1-130<br>174, 1-149<br>249, 10-150<br>253, 12-247<br>320, 196-316<br>779, 646-778<br>871, 740-860<br>926, 787-921<br>927, 787-918<br>927, 787-918<br>994, 856-985<br>997, 863-995<br>1032, 890-1026	F, Aq
Mariprofundus ferrooxydans	ZP_01451258.1 GI:114775690<br>ZP_01451244.1 GI:114775676	143<br>373, 3-131	Aq

No. genomes with Hrs/Total	2/2		
Spirochaetes			
Treponema denticola	NP_971622.1 GI:42526524<br>NP_971908 GI:42526810	137<br>143	An, HA
No. genomes with Hrs/Total	1/5		
No. genomes with Hrs/Total	115/440		

[1] Based on NCBI Taxonomy Database (www.ncbi.nlm.nih.gov/Taxonomy/Browser/wwwtax.cgi and www.ncbi.nlm.nih.gov /sutils/genom_table.cgi).

[2] From www.ncbi.nlm.nih.gov/genomes/lproks/cgi. Abbreviations: Ae – aerobe; An – anaerobe; Aq – aquatic; F – facultative; HA – host associated; M – multiple habitats; MAe – microaerophilic; Sp – specialized; Th – thermophilic; Tr - terrestrial.

Table 4. Hemerythrin sequences with alterations at the seven iron-coordinating positions.

Name	Accession No.	Remarks	FUGUE Z score
Anaeromyxobacter sp. Fw109-5	ZP_01669455|GI:121537679<br>ZP_01671347|GI:121539576	Leu/His in helix A<br>Gln/His in helix A and in helix B	18.7<br>18.5
Anaeromyxobacter dehalogenans	YP_463433|GI:86156648	Gln/His in helix C	19.0
Azoarcus sp.EbN1	YP_157941|GI:56476352	Ser/His in helix C	7.2
Burkholderia phymatum	ZP_01504458|GI:118033012	Ser/His in helix C	6.8
Caldicellulosiruptor saccharolyticus	ZP_00885869|GI:82500456	Ile/His in helix A	20.8
Campylobacter coli	ZP_00367964|GI:57168833	No helix D	15.8
Campylobacter upsaliensis	ZP_00371609|GI:57505683	No helix A	11.9
Clostridium beijerincki	ZP_00910041|GI:82747545<br>ZP_00911352|GI:82748877<br>ZP_00908279|GI:82745769<br>ZP_00909310|GI:82746807<br>AALO01000013|EAP60047.1	Gln/His in helix C<br>Gln/His in helix C<br>Gln/His in helix C<br>Gln/His in helix C<br>Gln/His in helix C	17.3<br>19.3<br>21.3<br>17.7<br>21.3
Clostridium cellulolyticum	[ZP_00909308|GI:82746805	Gln/His in helix C	21.3
Clostridium thermocellum	ZP_00504934|GI:67875718	Gln/His in helix C	19.4
Dechloromonas aromatica	YP_284887|GI:71907300	Asp/His in helix B	20.8
Desulfitobacterium hafniense	ZP_01371607|GI:109647704<br>YP_516471|GI:89892984	Gln/His in helix C<br>Gln/His in helix C	17.2<br>21.1
Geobacter lovleyi	ZP_01593713|GI:118745747	Gln/His in helix B	7.9
Geobacter uraniumreducens	ZP_01143645|GI:88938143	Gln/His in helix C	26.7
Janthinobacterium lividum	ABK64032|GI:118161348	Asp/His in helix B	24.4
Kineococcus radiotolerans	AAEF02000092|EAM73238.1	Ile/His in helix C; Glu/Asp in helix D	8.1
Lumbricus rubellus	CF426790	Asn/His in helix C	20.9
Magnetococcus sp. MC-1	YP_867464|GI:117926847<br>YP_864243|GI:117923626	Gln/His in helix C[1]<br>Gln/His in helix C[1]	19.8<br>23.4
Magnetospirillum magneticum	YP_419883|GI:83309619<br>YP_421315|GI:83311051<br>YP_420912|GI:83310648<br>YP_423659|GI:83313395<br>YP_420035|GI:83309771<br>YP_422017|GI:83311753<br>YP_421602|GI:83311338<br>YP_420779|GI:83310515<br>YP_422781|GI:83312517	Arg/His and Asp/His in helix C<br>Leu/His in helix C<br>Gln/His in helix C<br>Gln/His in helix C<br>Ile/His and Gln/His in helix C; Tyr/His in helix D<br>Asn/His in helix C<br>Asn/His in helix C<br>Asn/His in helix A; Asn/His in helix B<br>Arg/His in helix A; Ala/His in helix B	14.8<br>16.1<br>18.1<br>19.2<br>17.6<br>17.0<br>20.5<br>19.6<br>6.8

	YP_421350|GI:83311086<br>YP_422163|GI:83311899	Val/His and Gln/His in helix C; Tyr/His in helix D<br>Gln/His in helix A; Asn/His and Gln/Glu in helix B	21.0<br>20.6
Magnetospirillum magnetotacticum	ZP_00052849|GI:46202650<br>ZP_00053886|GI:23014047<br>ZP_00053732|GI:23013881<br>ZP_00055446|GI:23015677<br>ZP_00051444|GI:23010917<br>ZP_00052929|GI:46202606<br>ZP_00052752|GI:23012741<br>ZP_00055707|GI:23015944<br>ZP_00053639|GI:46202172<br>ZP_00055499|GI:23015731	Missing helix A<br>Arg/His and Thr/His in helix C<br>Leu/His in helix C<br>Met/His in helix C<br>Gln/His in helix C<br>Asn/His in helix C<br>Asn/His in helix C<br>Arg/His in helix A; Ile/His in helix B<br>Missing helix D<br>Ala/His in helix B	13.1<br>23.2<br>18.8<br>17.7<br>18.8<br>17.6<br>17.6<br>8.0<br>9.1<br>18.1
Marine γ-proteobacterium	ZP_01224113|GI:90416181	Glu/His in helix A	17.6
Myxococcus xanthus	YP_635509|GI:108758665	Gln/His in helix C	8.2
Novosphingobium aromaticivorans	YP_497813|GI:87200556	Ser/His in helix C; Glu/Asp in helix D	6.5
Pseudomonas mendocina	ZP_01526847|GI:118058386	Glu/His in helix C; Glu/Asp in helix D	6.6
Pseudomonas resinovorans	BAB32774|GI:13094185	No His or Asp in helix D	17.5
Pseudomonas syringae	YP_235578|GI:66045737	Ala/His in helix C	6.9
Ralstonia pickettii	ZP_01661972|GI:121529360	Ala/His in helix C; Glu/Asp in helix D	6.5
Rhodospirillum rubrum	YP_427592|GI:83593840	Met/His in helix B[2]	20.4
Shewanella woodyi	ZP_01539832|GI:118071638	Gln/His in helix C	16.1
Sphingomonas sp. SKA58	ZP_01303096|GI:94496520	Glu/His in helix C[3]	6.9
Sphingomonas wittichii	ZP_01607194|GI:118759500	Leu/His in helix C	8.4
Thiobacillus denitrificans	YP_314491|GI:74316751	Lys/His in helix C	7.9
Vibrio parahaemolyticus	NP_797668|GI:28898063	Tyr/His in helix B[4]	19.1

[1] Methyl-accepting chemotaxis sensory transducer.
[2] Response regulator receiver domain protein (CheY).
[3] But there is a His next to the Glu (see alignment in Fig. 2 Supplemental Information).
.[4] GGDEF family protein.

Table 5. Numbers of alterations to the His resIdues coordinating the two Fe atoms in the four helices of bacterial Hrs.

Alteration	A	B	C	D	Total
Gln / His	2	2	20	0	24
Asn / His	1	2	5	0	8
Lys, Arg / His	2	0	3	0	5
Ala, Val, Leu, Ile, Met, Tyr / His	2	5	9	2	18
Glu, Asp / His	1	2	4	0	7
Ser, Thr / His	0	0	3	0	3
Others	0	0	0	2	2
Helix missing	2	0	0	2	4
Total	10	11	44	6	71
